# The complete functional characterisation of the terpene synthase family in tomato

**DOI:** 10.1111/nph.16431

**Published:** 2020-02-19

**Authors:** Fei Zhou, Eran Pichersky

**Affiliations:** ^1^ Department of Molecular, Cellular, and Developmental Biology University of Michigan Ann Arbor MI 48109 USA

**Keywords:** functional characterisation, prenyltransferase, specialised metabolism, subcellular localisation, terpene synthase, terpenes, tomato

## Abstract

- Analysis of the updated reference tomato genome found 34 full‐length TPS genes and 18 TPS pseudogenes.
- Biochemical analysis has now identified the catalytic activities of all enzymes encoded by the 34 TPS genes: one isoprene synthase, 10 exclusively or predominantly monoterpene synthases, 17 sesquiterpene synthases and six diterpene synthases. Among the monoterpene and sesquiterpene and diterpene synthases, some use *trans*‐prenyl diphosphates, some use *cis*‐prenyl diphosphates and some use both. The isoprene synthase is cytosolic; six monoterpene synthases are plastidic, and four are cytosolic; the sesquiterpene synthases are almost all cytosolic, with the exception of one found in the mitochondria; and three diterpene synthases are found in the plastids, one in the cytosol and two in the mitochondria.
- New *trans*‐prenyltransferases (TPTs) were characterised; together with previously characterised TPTs and *cis*‐prenyltransferases (CPTs), tomato plants can make all *cis* and *trans* C_10_, C_15_ and C_20_ prenyl diphosphates. Every type of plant tissue examined expresses some TPS genes and some TPTs and CPTs.
- Phylogenetic comparison of the TPS genes from tomato and Arabidopsis shows expansions in each clade of the TPS gene family in each lineage (and inferred losses), accompanied by changes in subcellular localisations and substrate specificities.

## Introduction

Terpenoids are a class of compounds, made of isoprene building blocks, that are found in all living organisms (Gershenzon & Dudareva, 2007). In plants, terpenoids play essential roles in myriad general physiological and biochemical processes such as photosynthesis, electron transport, developmental regulation and membrane architecture, with many of these roles shared with other organisms (Pichersky & Raguso, 2018). However, the majority of terpenoids in plants identified by far are those that evolved in different lineages as adaptations for specific ecological niches and are therefore defined as specialised terpenoid metabolites. Such metabolites serve in attracting pollinators and seed dispersers, in defence against pathogens and herbivores, and in attracting useful soil microorganism (Gershenzon & Dudareva, 2007; Pichersky & Raguso, 2018). Because of their functions, biosynthesis of specialised terpenes in plants is usually restricted to specific tissues or cell types, such as flowers (Dudareva *et al*., 1998, 2003; Chen *et al*., 2003; Tholl *et al*., 2004), roots (Chen *et al*., 2004; Vaughan *et al*., 2013) or glandular trichomes found at the surface of leaves, stems and fruits (Iijima *et al*., 2004; Schilmiller *et al*., 2009; Booth *et al*., 2017).

Regular terpenoids are synthesised by condensing one dimethylallyl diphosphate (DMAPP) molecule with one or more molecules of its isomer, isopentenyl diphosphate (IPP) (McGarvey & Croteau, 1995). In plants, these C_5_ precursors are the products of two separate pathways, the cytosolic mevalonate (MVA) pathway and the plastidic 2‐*C*‐methyl‐d‐erythritol 4‐phosphate (MEP) pathway (Vranová *et al*., 2013). IPP and DMAPP are further converted by *trans*/*cis*‐prenyltransferases (TPTs/CPTs) to polyprenyl diphosphates such as geranyl/neryl diphosphate (GPP/NPP, C_10_; neryl is the *cis*‐isomer of geranyl), *trans*/*cis*‐farnesyl diphosphate (*E*,*E*‐FPP/*Z*,*Z*‐FPP, C_15_), geranylgeranyl/nerylneryl diphosphate (GGPP/NNPP, C_20_) or longer‐chain prenyl diphosphates. The C_5_–C_25_ prenyl diphosphate precursors are subsequently utilised by a large family of structurally related enzymes known as terpene synthase/cyclases (TPSs) to form the basic skeletons of isoprene (C_5_), monoterpenes (C_10_), sesquiterpenes (C_15_), diterpenes (C_20_) and sesterterpenes (C_25_) (Bohlmann *et al*., 1998; Chen *et al*., 2011; Sharkey *et al*., 2013; Huang *et al*., 2017). The terpene skeletons can be further modified by various enzymes, such as the cytochrome P450 oxygenases (CYPs), dehydrogenases, methyltransferases, acyltransferases, and glycosyltransferases to form more diverse compounds (Pichersky *et al*., 2006; Boutanaev *et al*., 2015).

TPS enzymes fall into two classes, type I and type II, based on structure and catalytic mechanisms. Type I TPS enzymes contain the aspartate‐rich DDxx(D,E) motif at their C‐terminal domain, called the ‘α domain’, that binds the metal cofactor (Mg^2+^ or Mn^2+^) that interacts with the prenyl diphosphate substrates and facilitates the substrate cation formation (Christianson, 2017). Type II terpene synthases contain a DxDD motif in the ‘β domain’ near the N‐terminus, with the second aspartate essential for the protonation‐initiated cyclisation of GGPP to form copalyl diphosphate (CPP) or other cyclic diterpene diphosphates (Zerbe & Bohlmann, 2015). The single functional TPS gene in *Physcomitrella patens* is bifunctional, encoding an enzyme with both type I and type II active domains. This enzyme first catalyses the conversion of GGPP to CPP with its β domain, then of CPP to *ent*‐kaurene (a precursor of gibberellins) with its α domain (Hayashi *et al*., 2006). However, angiosperms have a separate gene for CPP synthase (CPS) in which the α domain is nonfunctional, and a second gene for kaurene synthase (KS), in which the β domain is nonfunctional (Köksal *et al*., 2011). These two genes appear to have evolved by gene duplication of the original CPS/KS bifunctional gene, followed by divergence (Gao *et al*., 2012). The TPS gene family in plants continued to evolve by gene duplication and divergence, with some diterpene synthases still containing both functional domains, but many diterpene synthases and all monoterpene, sesquiterpene, and sesterterpene synthases containing only the functional α domain and some diterpene synthases containing only the functional β domain (Gao *et al*., 2012). Phylogenetic analysis of plant TPS genes/proteins has divided these into seven subfamilies: type I TPS sequences form clades TPS‐a, TPS‐b, TPS‐d (gymnosperm‐specific), TPS‐e/f, TPS‐g and TPS‐h (specific to *Selaginella* spp.); type II TPSs form clade TPS‐c (Chen *et al*., 2011).

It has generally been observed that monoterpenes and diterpenes are produced in the plastids by the respective terpene synthases, where GPP and GGPP are produced as well, while sesquiterpenes are produced by sesquiterpene synthases in the cytosol, where FPP is synthesised (Vranová *et al*., 2013). However, over the last decade, multiple researchers have reported the TPS‐catalysed formation of diterpenes and monoterpenes in the cytosol (Aharoni *et al*., 2004; Herde *et al*., 2008; Dong *et al*., 2013; Falara *et al*., 2014) and sesquiterpenes in the plastids (Sallaud *et al*., 2009).

The TPS gene family has been studied most extensively in the model plant Arabidopsis (*Arabidopsis thaliana*). In total, 32 TPS genes encode potentially functional TPSs in at least one accession (Aubourg *et al*., 2002), but still the functions of seven of these have not been determined in any accession (Tholl & Lee, 2011; Q. Wang *et al*., 2016; Huang *et al*., 2017). Of the TPS genes characterised so far in Arabidopsis, six encode monoterpene synthases, six encode sesquiterpene synthases, eight encode diterpene synthases, and five encode sesterterpene synthases (Chen *et al*., 2011; Huang *et al*., 2017). In addition to Arabidopsis, genome‐wide analyses of the TPS gene family have been conducted in other plant and algal species, such as red algae (Wei *et al*., 2019), the nonvascular moss *Physcomitrella patens* (Hayashi *et al*., 2006) and liverwort *Marchantia polymorpha* (Kumar *et al*., 2016a), the nonseed vascular plant *Selaginella moellendorffii* (Li *et al*., 2012), gymnosperms *Picea* spp. (Keeling *et al*., 2011), and multiple angiosperm species (Martin *et al*., 2010; Falara *et al*., 2011; Zhuang *et al*., 2012; Alquézar *et al*., 2017; Booth *et al*., 2017). These analyses have revealed that the size of the plant TPS gene family varies from one functional gene in *Physcomitrella patens* (Chen *et al*., 2011) to 69 putatively functional genes in *Vitis vinifera* (Martin *et al*., 2010). However, to date, in no species has the complete set of the TPS genes been functionally characterised.

Previous studies have already indicated that tomato (*Solanum lycopersicum*) plants synthesise an unusual number and types of specialised terpenes, particularly in their trichomes (Falara *et al*., 2011; McDowell *et al*., 2011). A unique feature of terpenoid metabolism in tomato and other *Solanum* species is the use by TPS enzymes of both *trans*‐ and *cis*‐prenyl diphosphates (Sallaud *et al*., 2009; Schilmiller *et al*., 2009). The release of the first high‐quality genome sequence of tomato (Tomato Genome Consortium, 2012) made possible a thorough examination of its TPS gene family, albeit 10 of the identified 30 functional genes (out of a total of 45 loci) could not be functionally characterised (Colby *et al*., 1998; van Schie *et al*., 2007; Schilmiller *et al*., 2009, 2010; Bleeker *et al*., 2011; Falara *et al*., 2011, 2014; Matsuba *et al*., 2013, 2015). Based on the analysis of the latest tomato genome release (2017 release of v. SL3.0), we now report that the tomato genome contains 34 functional TPS genes, and we have successfully determined the catalytic activities, gene expression patterns and subcellular localisations of this entire group of enzymes. These results make the tomato TPS family the only one to be fully characterised so far.

## Materials and Methods

### Plant materials and chemicals

Tomato (*Solanum lycopersicum* L. cv MP1) seeds were obtained from the Tomato Genetic Resource Center (https://tgrc.ucdavis.edu). Throughout the text, when not specifically indicated, the tomato plants used were of cv MP1. *Nicotiana benthamiana* and *Arabidopsis thaliana* (Col‐0 ecotype) seeds were obtained from our laboratory and Arabidopsis Biological Resource Center, respectively. Details of plant growth conditions can be found in Supporting Information Methods S1.

IPP, DMAPP, GPP, NPP, *E*,*E*‐FPP, *Z*,*Z*‐FPP and GGPP were obtained from Echelon Biosciences (Salt Lake City, UT, USA). Terpene standards were purchased from Sigma Aldrich (St Louis, MO, USA). All other chemicals and solvents were obtained from Fisher Scientific (Hampton, NH, USA).

### Identification of new TPS gene models in the tomato genome

The most recent tomato genome (https://solgenomics.net) was used for blastn searches with known tomato TPS genomic sequences. The resulting candidates were manually annotated to accomplish a more accurate result. To verify and fill the gaps of the newly discovered TPS sequences (*TPS47* through *TPS53*), genomic DNA was extracted from tomato leaves and used as template for PCR with gene‐specific primers (Table S1). PCR amplification was performed with KOD Hot Start DNA polymerase (EMD Millipore, Burlington, MA, USA). DNA fragments obtained were extracted with EZNA Gel Extraction Kit (Omega Bio‐tek, Norcross, GA, USA) and then verified by the Sanger sequencing method.

### RNA isolation, cDNA synthesis and quantitative real‐time PCR

Different tissues of *S. lycopersicum* plants were collected and immediately frozen in liquid nitrogen and stored at −80°C until use. Trichomes were collected by gently shaking the corresponding tissues in liquid nitrogen. Details of RNA isolation, cDNA synthesis and quantitative real‐time PCR (RT‐qPCR) can be found in Methods S1.

### Isolation of full‐length TPS and prenyltransferase cDNAs

The full‐length cDNAs of *TPS10*, *TPS16*, *TPS27*, *TPS28*, *TPS33*, *TPS35* and *TPS36* were obtained by PCR amplification with gene‐specific primers (Table S1) based on a previous publication (Falara *et al*., 2011). The full‐length cDNAs of *TPS25*, *TPS47*, *TPS48*, *TPS51*, *TPS52*, *GGPPS3*, *SSU I*, *SSU II*, *TPT1* and *TPT2* were obtained by PCR amplification with gene‐specific primers (Table S1) based on annotated results from the genome database and our manual annotation (Fig. S1). The cDNA used as template was synthesised from MP1 leaf, flower, mature red fruit, stem trichome and root RNA. The other TPS and prenyltransferase genes were obtained as described by Falara *et al*. (2011) (*TPS24* and *TPS40*), Matsuba *et al*. (2013) (*TPS18* and *TPS41*), Akhtar *et al*. (2013) (*CPT1*, *CPT2* and *CPT6*), Gaffe *et al*. (2000) (*FPPS1*) and Ament *et al*. (2006) (*GGPPS1* and *GGPPS2*). Amino acid sequences of all cloned TPS and prenyltransferase genes from MP1 are identical to the tomato reference genome obtained from Heinz 1706.

### Synthesis of codon‐optimised genes

*Escherichia coli* codon‐optimised versions of *TPS10*, *TPS16*, *TPS47*, *TPS51* and *TPS52* were synthesised by Gene Universal (Newark, DE, USA).

### Multiple sequence alignment and phylogenetic analysis

The full‐length amino acid sequences were aligned using the clustalw program (Thompson *et al*., 1994) with default parameters. A maximum‐likelihood phylogenetic tree was constructed using the Mega7 tool (Kumar *et al*., 2016b) with default settings, bootstrap values were performed with 1000 repetitions. The scale bar corresponds to 20% amino acid sequence divergence.

### Protoplast transformation and confocal microscopy

Regions corresponding to the first *c*. 120 or full‐length amino acids of the tested proteins were amplified (detailed in Table S1), digested accordingly and ligated into pEZS‐NL vector (Zhou *et al*., 2017), creating an in‐frame C‐terminal fusion protein with EGFP. The resulting constructs were applied for PEG‐mediated transient expression in Arabidopsis mesophyll protoplasts as described before (Yoo *et al*., 2007). MitoTracker Red (Invitrogen, Carlsbad, CA, USA) was used as a mitochondrial marker. Fluorescence signals were observed using a Leica SP5 laser scanning confocal microscope as described previously (Falara *et al*., 2011). All the transient expression experiments were repeated independently at least three times.

### Expression in *Escherichia coli* and TPS enzyme assays

TPS genes without their fragments corresponding to putative transit peptides were cloned in pET32b or pET28a to express TPS‐His recombinant proteins (detailed in Table S1). Protein expression and purification are detailed in Methods S1.

TPS enzyme assays were performed as described previously (Falara *et al*., 2011) using GPP, NPP, *E*,*E*‐FPP, *Z*,*Z*‐FPP and GGPP as substrates. After enzyme assay incubation, products were extracted with methyl tert‐butyl ether (MTBE) and analysed using gas chromatography–mass spectrometry (GC–MS).

### Isoprene synthase enzyme assays

Enzymatic assays for isoprene synthase were performed as previously described (Sasaki *et al*., 2005) using DMAPP as the substrate. After enzyme assay incubation, headspace products were collected with a 100 μm solid‐phase micro extraction (SPME) fibre (Supelco, St Louis, MO, USA) at 42°C for 15 min and analysed by GC–MS.

### Prenyltransferase assays

For enzymatic assays, truncated *trans*‐prenyltransferases (TPTs) without stop codons were cloned into pET32b upstream and in‐frame of the (His)_6_‐tag sequence to express TPT‐His recombinant proteins. Truncated SSU I and SSU II were cloned into pET28a downstream and in‐frame of the (His)_6_‐tag sequence to express His‐SSU recombinant proteins. For co‐expression with His‐SSU, truncated GGPPSs with stop codons were subcloned into pET32b to express nontagged GGPPS proteins (detailed in Table S1).

The prenyltransferase assays were performed using IPP and DMAPP as substrates as described previously (Zhou *et al*., 2017). After enzyme assay incubation, products were hydrolysed to their corresponding alcohols, extracted with MTBE and analysed by GC–MS. To calculate the molar ratio of formed products, standard curves were constructed using commercial geraniol, *E*,*E*‐farnesol and geranylgeraniol.

### *Agrobacterium*‐mediated transient expression of heterologous proteins in tobacco

For constructing of plant transformation vectors, full‐length or truncated ORFs of the tested genes (detailed in Table S1) were amplified by PCR and cloned into the binary vector pEAQ‐HT (Sainsbury *et al*., 2009) that contains the cauliflower mosaic virus 35S promoter and nopaline synthase terminator. *In planta* transient expression in tobacco (*Nicotiana benthamiana*) leaves was performed as previously described (Sainsbury *et al*., 2009). A pEAQ‐HT construct carrying GFP alone was used as a control. Tobacco leaves were harvested 3 d after infiltration and immediately dipped in MTBE for 30 min to extract terpene products. The MTBE extracts were concentrated by evaporating the solvent to a final volume of *c*. 100 μl for GC–MS analysis.

### Profiling of tomato terpene volatiles and correlation analysis

All the tested tissues were freshly harvested and submerged in MTBE containing 10 ng μl^−1^ of the tetradecane internal standard. Metabolites were extracted for 15 min with gentle shaking and the resulting extracts were concentrated to a final volume of *c*. 100 μl for GC–MS analysis. The correlation analyses of tomato terpene synthase genes and volatile terpenes are detailed in Methods S1.

### GC–MS analysis of terpenes

The analysis of terpene products (C_10_–C_25_) was performed as described previously (Falara *et al*., 2011). Briefly, 1 μl of sample was autoinjected into a Shimadzu QP‐2010 SE GC‐MS system equipped with a Rxi‐5Sil column (30 m length, 0.25 mm i.d., and 0.25 μm film thickness; Restek, Bellefonte, PA, USA). Analysis of isoprene was performed by directly injecting the SPME fibre into the Shimadzu QP‐2010 SE GC‐MS system equipped with the Rxi‐5Sil column. The analysis of prenyltransferase assay products was performed using an EC‐WAX column (30 m length, 0.32 mm i.d., and 0.25 μm film thickness; Grace, Columbia, MD, USA). The identification methods for each terpene compound are listed in Table S2. Details of GC–MS parameters can be found in Methods S1.

## Results

### Identification of new TPS genes in the tomato genome

A search for TPS genes in the most recently updated tomato genome (https://solgenomics.net/organism/Solanum_lycopersicum/genome; the 2017 release of v. SL3.0) identified 52 gene models, of which 45 had previously been reported (*TPS1*–*TPS46*, not including *TPS45*, which is present in *S. habroichaites* but not in *S. lycopersicum*; Tables S3, S4). The seven additional loci were designated as *TPS47–TPS53*. *TPS47* and *TPS48* appeared to have uncompromised open reading frames (Table S3), whereas *TPS49*, *TPS50* and *TPS53* appeared to have mutations and deletions (Table S4). Genomic sequences of *TPS51* and *TPS52* had *c*. 1 kb gaps between exons 2 and 5 (Fig. S1). To verify the mutations and fill the gaps in the new gene sequences, genomic DNA of these seven genes was amplified by PCR and fully sequenced. The results indicated that *TPS47*, *TPS48*, *TPS51* and *TPS52* encoded potentially functional TPSs, with protein lengths of 562, 560, 550 and 551 amino acids, respectively (Table S3). By contrast, *TPS49*, *TPS50* and *TPS53* were pseudogenes (Table S4).

**Figure 1:**
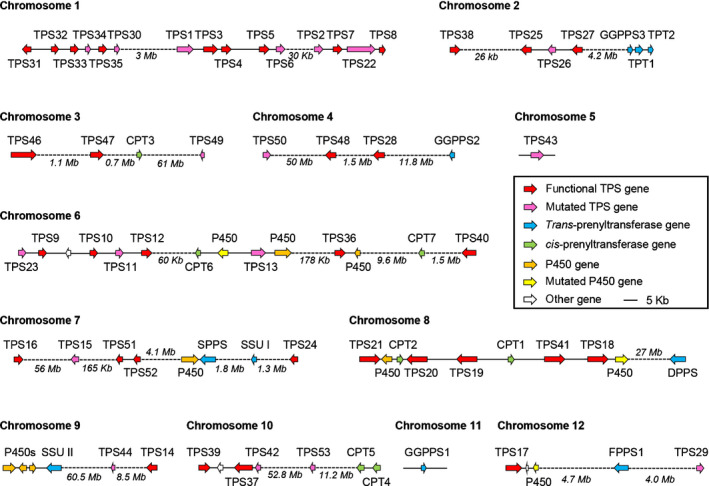
The organisation of tomato TPS genes and other related genes on the chromosomes. CPT, *cis*‐prenyltransferase; DPPS, decaprenyl diphosphate synthase; FPPS, farnesyl diphosphate synthase; GGPPS, geranylgeranyl diphosphate synthase; P450, putative cytochrome P450 oxidoreductase; SPPS, solanesyl diphosphate synthase; SSU, small subunit of geranyl diphosphate synthase; TPS, terpene synthase; TPT, *trans*‐prenyltransferase.

**Figure 2:**
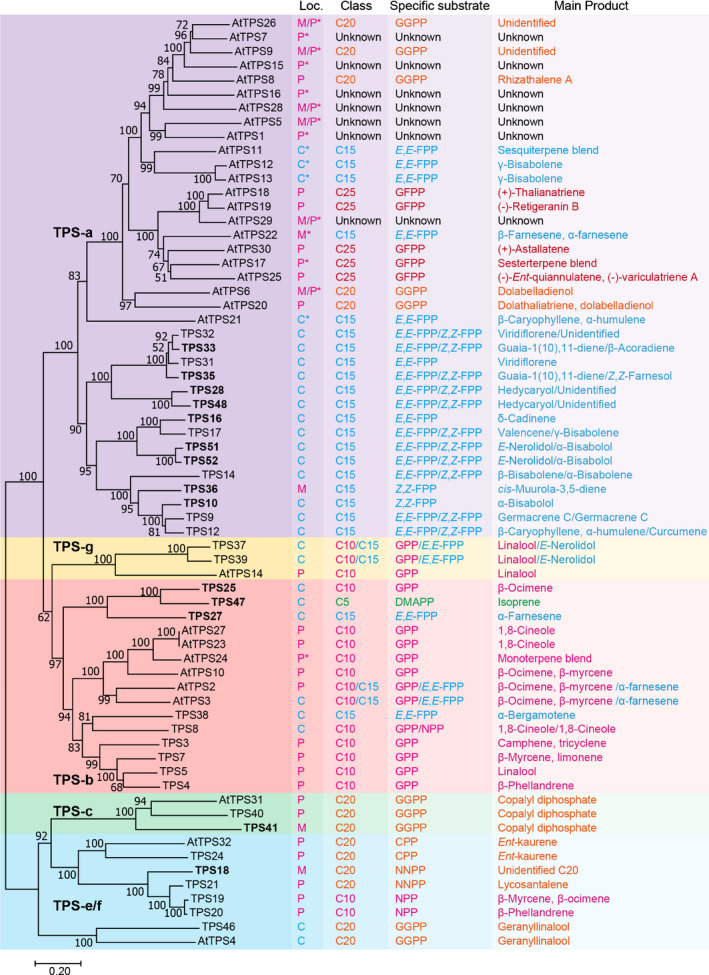
Phylogenetic analysis of tomato and Arabidopsis TPS gene family. A maximum‐likelihood phylogenetic tree of the TPS proteins is shown on the left panel, while subcellular localisation (Loc.), substrate class, specific substrate and main product of the corresponding enzymes are shown on the right four panels. The TPS‐a, TPS‐g, TPS‐b, TPS‐c and TPS‐e/f clades are shading in purple, yellow, pink, green and blue, respectively. Cytosolic (C) localisation is shown in blue, plastidic (P) and mitochondrial (M) localisations are shown in magenta. Localisations predicted by TargetP and ChloroP are denoted by asterisks. C_5_, C_10_, C_15_, C_20_ and C_25_ compounds are shown in green, magenta, blue, orange and red, respectively. Terpene synthases characterised in this work are shown in bold. At, *Arabidopsis thaliana*; CPP, *ent*‐copalyl diphosphate; DMAPP, dimethylallyl diphosphate; FPP, farnesyl diphosphate; GFPP, geranylfarnesyl diphosphate; GGPP, geranylgeranyl diphosphate; GPP, geranyl diphosphate; NNPP, nerylneryl diphosphate; NPP, neryl diphosphate; TPS, terpene synthase.

*TPS47* and *TPS49* are located on chromosome 3, *TPS48* and *TPS50* are located on chromosome 4, *TPS51* and *TPS52* are close to each other and in the same orientation on chromosome 7, and *TPS53* resides on chromosome 10 (Fig. 1). Phylogenetic analysis indicates that TPS47 belongs to the TPS‐b clade, together with five previously characterised monoterpene synthases, one previously characterised sesquiterpene synthase and two uncharacterised enzymes (TPS25 and TPS27) (Fig. 2). TPS48, TPS51, TPS52 and six other uncharacterised enzymes (TPS10, TPS16, TPS28, TPS33, TPS35 and TPS36, which was previously shown to have activity with *Z*,*Z*‐FPP, but the structure of the sesquiterpene product could not be identified) (Falara *et al*., 2011), belong to the TPS‐a clade, a clade that also contains seven previously characterised enzymes, all of which are sesquiterpene synthases. Thus, the tomato genome contains at least 52 TPS genes including 34 putative functional TPSs, among these 14 (the 12 genes listed above, plus *TPS18* and *TPS41*, which belong to the e/f clade) have not been previously functionally characterised. To determine the terpene synthase activity of the 14 uncharacterised TPS enzymes, their full‐length cDNAs were obtained from different tissues and functionally tested by expression in *Escherichia coli* or in *Nicotiana benthamiana*.

### Characterisation of the enzymatic activities of 14 previously uncharacterised TPS genes

#### TPS enzymes of the TPS‐a clade – TPS48, TPS51, TPS52, TPS10, TPS16, TPS28, TPS33, TPS35 and TPS36

**Figure 3:**
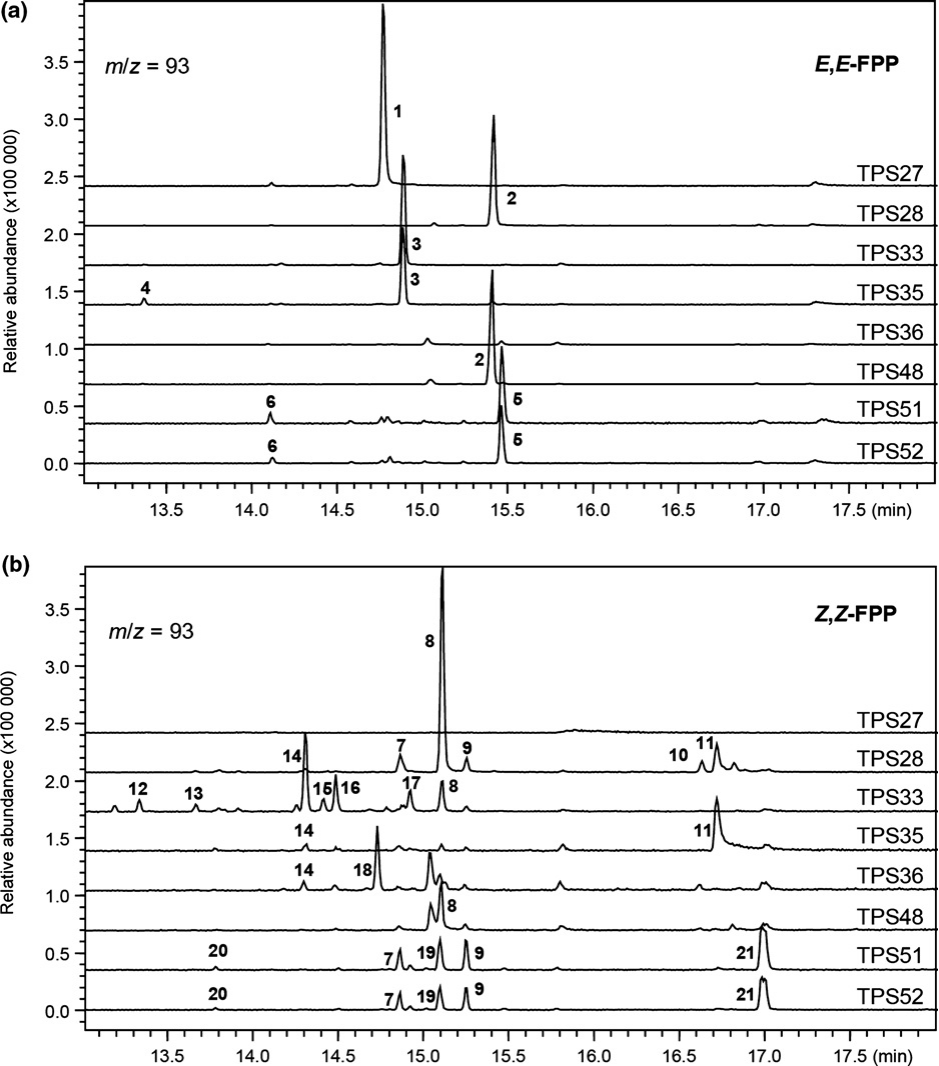
GC–MS analysis of the products formed *in vitro* by the enzymatic activities of tomato TPS proteins. Enzymes were incubated with *E*,*E*‐FPP (a) and *Z*,*Z*‐FPP (b) and products were analysed as described in the ‘Materials and Methods’ section. Reaction products were identified by comparison of their mass spectra and retention indices with authentic standards and NIST libraries: 1, α‐farnesene; 2, elemol; 3, guaia‐1(10),11‐diene; 4, β‐elemene; 5, *E*‐nerolidol; 6, *E*‐β‐farnesene; 7, *E*‐β‐bisabolene; 8, unknown 1; 9, α‐bisabolene; 10, α‐eudesmol; 11, (*Z*,*Z*)‐farnesol; 12, α‐cedrene; 13, *cis*‐α‐bergamotene; 14, β‐acoradiene; 15, unknown 2; 16, unknown 3; 17, β‐curcumene; 18, *cis*‐muurola‐3,5‐diene; 19, *Z*‐nerolidol; 20, *Z*‐β‐farnesene; 21, α‐bisabolol. Mass spectra and retention indices of the terpene products can be found in Supporting Information Fig. S3 and Table S2, respectively. FPP, farnesyl diphosphate; TPS, terpene synthase.

All soluble TPS proteins (Fig. S2) described in this section (and subsequent ones) were assayed *in vitro* using GPP, NPP, *E*,*E*‐FPP, *Z*,*Z*‐FPP and GGPP. Results indicated that both TPS28 and TPS48 (which show 85% amino acid sequence identity) used *E*,*E*‐FPP to produce a product identified as elemol (Figs 3a, S3; Table S2); elemol is reported to be the thermal breakdown product of (+)‐hedycaryol (Fig. S4; Hattan *et al*., 2016). TPS28 and TPS48 also catalysed the formation of a major sesquiterpene from *Z*,*Z*‐FPP that is currently unidentified (Figs 3b, S3; Table S2). TPS33 and TPS35 (91.7% identical) both catalysed the formation of guaia‐1(10),11‐diene from *E*,*E*‐FPP (Figs 3a, S3; Table S2). When assayed with *Z*,*Z*‐FPP, TPS33 made several sesquiterpenes including β‐acoradiene, β‐curcumene, α‐cedrene and *cis*‐α‐bergamotene, while TPS35 made predominantly *Z*,*Z*‐farnesol (Figs 3b, S3; Table S2). TPS36 only had activity using *Z*,*Z*‐FPP and produced mostly *cis*‐muurola‐3,5‐diene (Figs 3b, S3; Table S2). TPS51 and TPS52 (94.7% sequence identity) catalysed the formation of mostly *E*‐nerolidol from *E*,*E*‐FPP (Figs 3a, S3; Table S2) and several sesquiterpenes (α‐bisabolol, *Z*‐nerolidol, α‐bisabolene, β‐bisabolene and *Z*‐β‐farnesene) from *Z*,*Z*‐FPP (Figs 3b, S3; Table S2). No activity was observed for either of these proteins with any of the other tested substrates.

**Figure 4:**
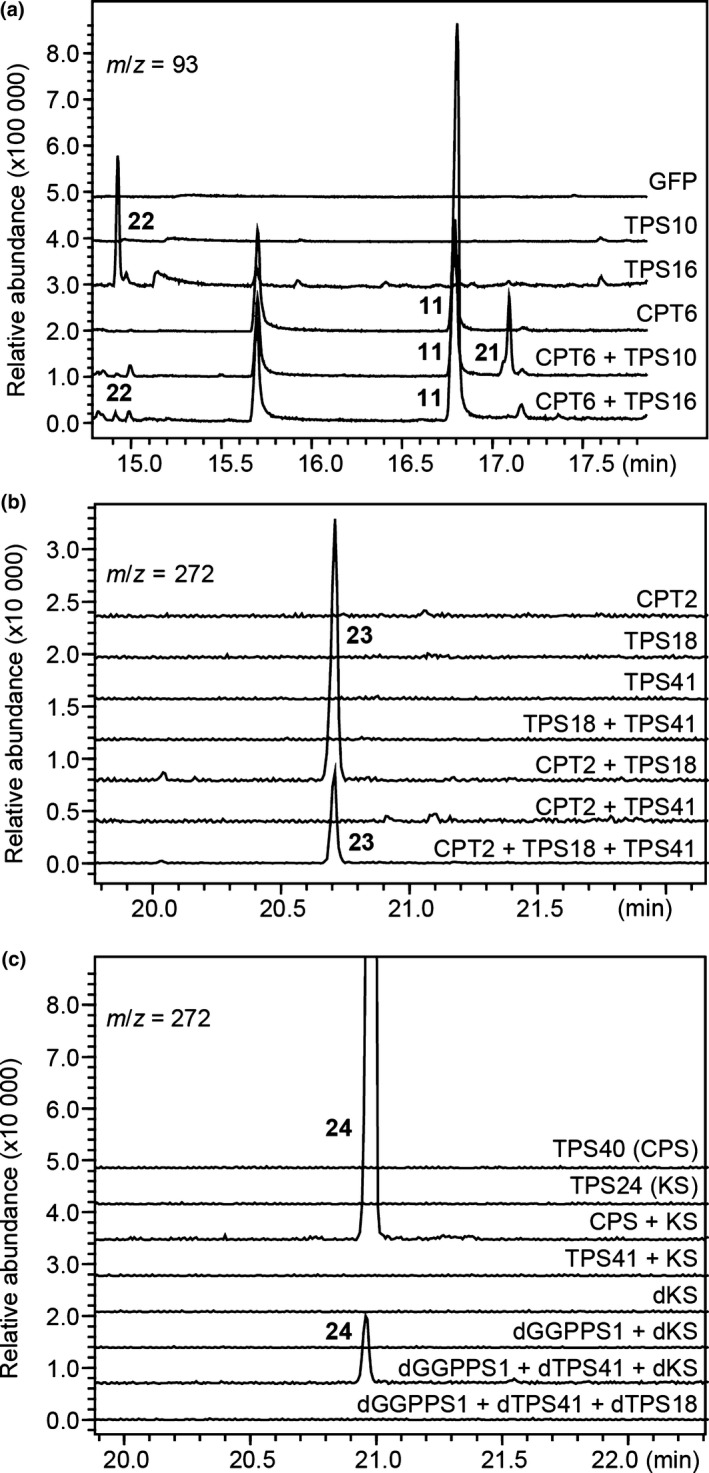
GC–MS analysis of the products formed in planta by transiently expressing TPS genes in *Nicotiana benthamiana* leaves. (a) Analysis of sesquiterpenes formed by *TPS10* and *TPS16*. *Nicotiana benthamiana* leaves expressing *GFP* alone was used as a negative control. (b) Analysis of the formation of an unknown diterpene by co‐expressing *TPS18* with *CPT2*. (c) Analysis of the formation of *ent*‐kaurene by expressing the full‐length and transit peptide deleted version (‘d’) of TPS genes. Products were extracted with methyl tert‐butyl ether (MTBE) and analysed as described in the ‘Materials and Methods’ section. Reaction products were identified by comparison of their mass spectra and retention indices with authentic standards and NIST libraries: 11, (*Z*,*Z*)‐farnesol; 21, α‐bisabolol; 22, δ‐cadinene; 23, unknown 4; 24, *ent*‐kaurene. Mass spectra and retention indices of the terpene products can be found in Supporting Information Fig. S3 and Table S2, respectively. CPS, *ent*‐copalyl diphosphate synthase; CPT, *cis*‐prenyltransferase; GGPPS, geranylgeranyl diphosphate synthase; KS, *ent*‐kaurene synthase; TPS, terpene synthase.

We could not obtain soluble proteins from *TPS10* and *TPS16* when expressed in *E. coli*, and we therefore transiently expressed their full‐length cDNAs in *N. benthamiana*. Using this approach, we determined that TPS16 catalysed the formation of δ‐cadinene from the endogenous *E*,*E*‐FPP pool (Figs 4a, S3; Table S2). However, no product was detected when *TPS10* was expressed alone (Fig. 4a). *TPS10* is located on chromosome 6 *c*. 60 kb away from *CPT6*, the gene encoding the *cis*‐prenyltransferase responsible for the biosynthesis of *Z*,*Z*‐FPP (Fig. 1), and when *TPS10* was co‐expressed with *CPT6* in *N. benthamiana*, the formation of α‐bisabolol was detected (Figs 4a, S3; Table S2). TPS10 and TPS16 showed no activity when their genes were co‐expressed with either *CPT1* or *CPT2* (Fig. S5).

#### TPS enzymes of the TPS‐b clade – TPS25, TPS27 and TPS47

**Figure 5:**
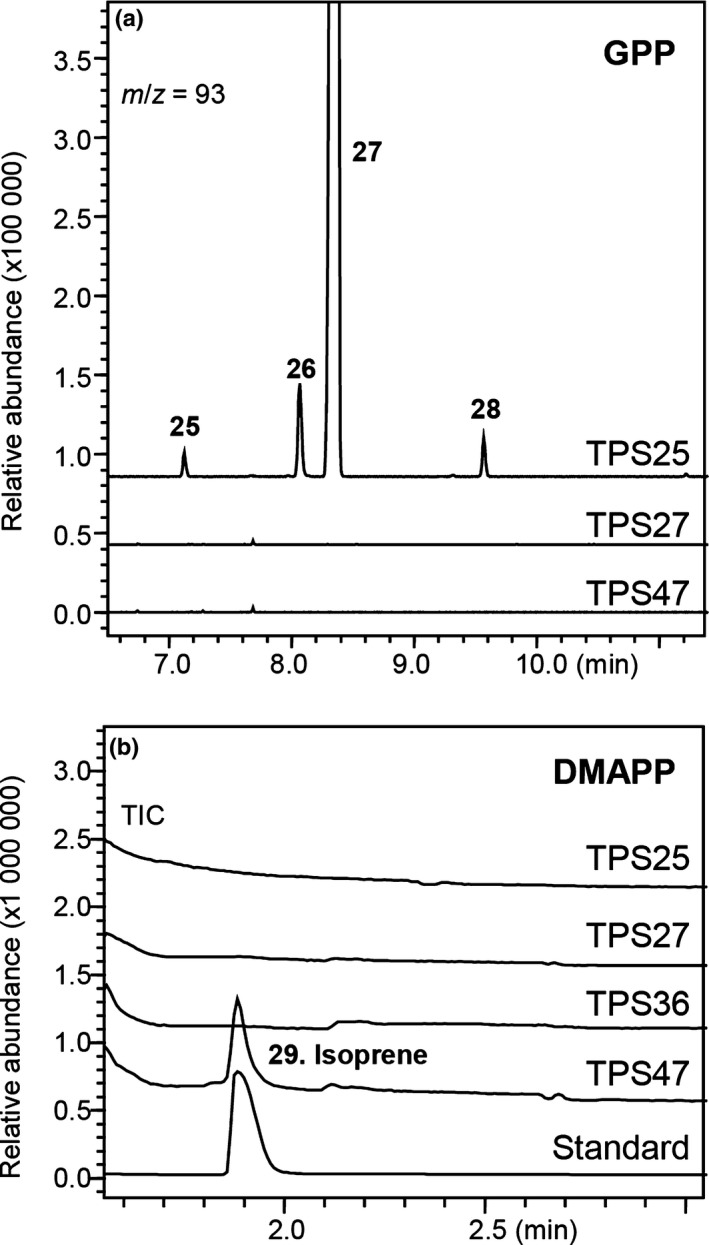
GC–MS analysis of the products formed *in vitro* by the enzymatic activities of tomato terpene synthase (TPS) proteins using geranyl diphosphate (GPP) (a) and dimethylallyl diphosphate (DMAPP) (b) as substrates. Reaction products were collected and analysed as described in the ‘Materials and Methods’ section, commercial isoprene was used as a standard. Reaction products were identified by comparison of their mass spectra and retention indices with authentic standards and NIST libraries: 25, β‐myrcene; 26, β‐*cis*‐ocimene; 27, β‐*trans*‐ocimene; 28, linalool; 29, isoprene. Mass spectra and retention indices of the terpene products can be found in Supporting Information Fig. S3; Table S2, respectively.

These three enzymes fall into a separate subclade within the TPS‐b clade (Fig. 2). TPS25 had activity only with GPP as the substrate, producing mostly β‐ocimene (Figs 5a, S3; Table S2). TPS27 had activity only with *E*,*E*‐FPP to form α‐farnesene (Figs 3a, S3; Table S2). TPS47 showed no activity towards any of the initially tested substrates (GPP, NPP, *E*,*E*‐FPP, *Z*,*Z*‐FPP and GGPP). Further phylogenetic analysis with terpene synthases from other plants indicated that isoprene synthases (IspSs), which catalyse the enzymatic conversion of DMAPP to isoprene, from various species are closely related to these three enzymes (Fig. S6a). Structural and functional analysis have established four ‘isoprene score’ amino acids that are specific to isoprene synthases (Köksal *et al*., 2010; Sharkey *et al*., 2013; Ilmén *et al*., 2015). These four amino acids are F338, S445, F485 and N505 (numbering refers to IspS from *Populus alba*) (Fig. S6b), of which only F338 is strictly conserved in all functional IspSs presently known, and when both F338 and F485 are present, the enzyme is always isoprene synthase (Ilmén *et al*., 2015). Among the tomato TPS enzymes, TPS36 has the first Phe residue, while TPS47 contains both Phe residues (Fig. S6b). When tested *in vitro*, TPS47 catalysed the formation of isoprene from DMAPP (Figs 5b, S3). By contrast, no isoprene synthase activity was observed for TPS25, TPS27 or TPS36 (Fig. 5b).

#### TPS enzymes of the TPS‐e/f and TPS‐c clades – TPS18 and TPS41

*TPS18* and *TPS41*, which were not previously biochemically characterised, belong to the e/f and c clades of the TPS family, respectively (Fig. 2). Proteins in these two clades are typically longer (*c*. 800 amino acids) than other TPS proteins, and past attempts to express such proteins, including TPS18 and TPS41, in *E. coli* have met with meagre success in obtaining soluble, enzymatically functional proteins (Falara *et al*., 2011; Matsuba *et al*., 2013). We transiently expressed these two genes in *N. benthamiana*, but no terpene products were observed (Fig. 4b). *TPS18* and *TPS41* are present in a cluster on chromosome eight where *CPT1* (encoding neryl diphosphate synthase) and *CPT2* (encoding nerylneryl diphosphate synthase) are also present (Fig. 1), suggesting that they may be functionally associated. To test this hypothesis, *TPS18* and *TPS41* were each co‐expressed with either *CPT1* or *CPT2* in *N. benthamiana* leaves. When *TPS18* was co‐expressed with *CPT2*, an unidentified diterpene was produced (Figs 4b, S3; Table S2), while no monoterpene was detected when co‐expressed with *CPT1* (Fig. S5). By contrast, *TPS41* showed no activity when co‐expressed with either *CPT1* (Fig. S5) or *CPT2* (Fig. 4b). Neither TPS18 nor TPS41 showed activity when their genes were co‐expressed with *CPT6* (Fig. S5).

TPS41 is most similar to TPS40, another TPS‐c member with a proven *ent*‐copalyl diphosphate synthase (CPS) activity (Bensen & Zeevaart, 1990; Falara *et al*., 2011). To test whether TPS41 has CPS activity, we co‐expressed *TPS41* with the previously characterised *ent*‐kaurene synthase (TPS24, KS; Falara *et al*., 2011). No terpene product was detected when the full‐length cDNAs of *TPS41* and *KS* were co‐expressed. By contrast, co‐expression of *TPS40* and *TPS24* led to the production of *ent*‐kaurene (Fig. 4c). TPS41 has a long N‐terminal extension (Fig. S7) and was predicted to be localised to the mitochondria by TargetP, which is different from the predicted plastidic localisation of KS (Fig. S8 and see below subcellular localisation section). We then co‐expressed the truncated versions of *TPS41*, *KS*, as well as *GGPPS1* (Ament *et al*., 2006) to provide GGPP in the cytosol, and *ent*‐kaurene was detected, indicating that TPS41 does have CPS activity (Figs 4c, S3; Table S2). Since the TPS18 protein was also shown to be in the mitochondria (see below subcellular localisation section), we expressed a *TPS18* construct lacking a transit peptide‐encoding region together with a similarly truncated construct for *TPS41* and *GGPPS1* in *N. benthamiana*, but no new product was detected in those plants (Fig. 4c).

### Characterisation of the enzymatic activities of previously uncharacterised *trans*‐prenyltransferases

**Figure 6:**
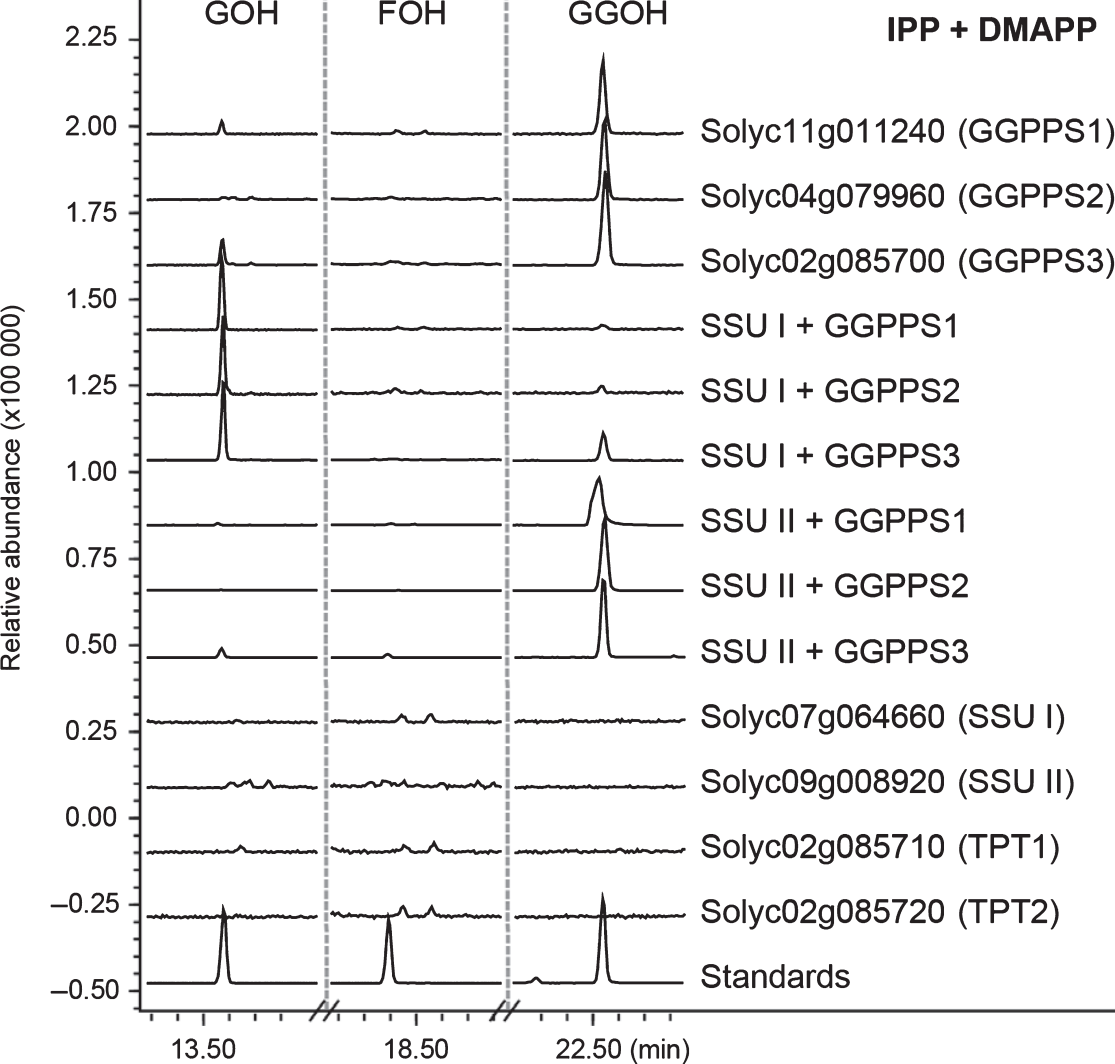
Characterization of the tomato *trans*‐prenyltransferase proteins. *In vitro* enzyme assays of recombinant transprenyltransferases (TPTs) and the geranylgeranyl diphosphate synthase (GGPPS)/SSU heterodimers with isopentenyl diphosphate (IPP) and dimethylallyl diphosphate (DMAPP). Products were hydrolysed to corresponding alcohols (GOH, geraniol; FOH, farnesol; GGOH, geranylgeraniol) and analysed with GC–MS. *m*/*z* = 93 was monitored for terpene products, only compounds corresponding to the prenyl alcohols were shown. SSU, small subunit of geranyl diphosphate synthase.

The *cis*‐prenyltransferase (CPT) gene family in tomato has been fully characterised (Akhtar *et al*., 2013), but the composition and enzymatic functions of the *trans*‐prenyltransferase (TPT) family members have not been thoroughly investigated. Our analysis shows that the tomato genome contains 10 putative full‐length TPTs (Fig. 1; Table S5), including one enzymatically characterised FPP synthase (FPPS1; Gaffe *et al*., 2000), two GGPP synthases (GGPPS1 and GGPPS2; Ament *et al*., 2006) and two long‐chain (C_45_ and C_50_) prenyl diphosphate synthases (SPPS and DPPS; Jones *et al*., 2013). Our analysis of the genome sequence also identified one homologue each of type I and type II small subunit genes of GPP synthases (GPPSs), called respectively SSU I and SSU II (Wang & Dixon, 2009) (Figs 1, S9; Table S5). SSU I and SSU II proteins are catalytically inactive by themselves but each is able to bind a subunit of GGPPS to form a heterodimer that can produce GPP (Orlova *et al*., 2009; Zhou *et al*., 2017). To examine the prenyltransferase activities of the newly identified TPT enzymes (Table S5), affinity‐purified His‐tagged recombinant proteins were incubated with IPP and DMAPP, and the hydrolysed products were analysed by GC–MS. Consistent with previous reports, GGPPS1 and GGPPS2 exhibited GGPPS activity, producing GGPP as the main product (66.3% and 86.3%, respectively) with small amounts of GPP (28.1% and 9.6%, respectively) and FPP (5.6% and 4.1%, respectively) (Fig. 6). Solyc02g085700 also showed predominantly GGPPS activity, producing a mixture of GGPP (61.2%), FPP (2.8%) and GPP (36.0%) (Fig. 6), and was thus designated as GGPPS3. No activity was observed for TPT1 and TPT2 (Fig. 6). SSU I and SSU II displayed no catalytic activity alone, but SSU I was able to change the product specificity of GGPPS1, GGPPS2 as well as GGPPS3 from GGPP to predominantly GPP (95.3%, 93.3% and 87.1%, respectively), while the heterodimers formed between SSU II and GGPPS1, GGPPS2 and GGPPS3 catalysed the formation of mainly GGPP (92.0%, 97.4% and 79.0%, respectively) (Fig. 6). Taken together, these results indicated that GPP and GGPP pools in tomato cells are provided by heteromeric GGPPSs/SSU I and three GGPPSs, respectively.

### The current tomato genome does not contain sesterterpene synthase genes

TPT1 and TPT2 are the only two remaining unidentified TPTs (Fig. 6). Phylogenetic analysis indicates that TPT1 and TPT2 are clustered with Arabidopsis GFPP synthases (Fig. S9), however, neither enzyme is predicted to have GFPP synthase activity by the well established ‘three floors’ model (Table S5; C. Y. Wang *et al*., 2016). Since no activity was detected when TPT1 and TPT2 were assayed *in vitro*, we co‐expressed both genes with a sesterterpene synthase (sesterTPS) gene (*AtTPS19*; Shao *et al*., 2017) in *N. benthamiana* cytosol to test whether they have GFPP synthase activity. As a positive control (−)‐retigeranin B was produced when *AtGFPPS2* was co‐expressed with *AtTPS19*, while no sesterterpenes were formed when either *TPT1* or *TPT2* was co‐expressed with *AtTPS19* (Fig. S10), indicating that TPT1 and TPT2 do not have GFPP synthase activity. Arabidopsis sesterTPSs belong to the TPS‐a clade (Fig. 2), and a recent study revealed that a single amino acid with a small side chain – Gly or Pro – near the active centre determines the substrate specificity of sesterTPSs (Chen *et al*., 2019), providing a large active‐site cavity for the larger GFPP substrates (Fig. S11a,c). However, TPS enzymes from tomato all possess a large‐side‐chain amino acid at the same site (Fig. S11a,b). These results suggest that the current tomato genome does not contain GFPPS or sesterTPS genes.

### Subcellular localisation of tomato TPS and TPT proteins

The subcellular localisation of only a handful of TPS enzymes in tomato has been reported. TPS14 was previously shown to be localised in the cytosol and TPS36 was targeted to the mitochondria (Falara *et al*., 2011). CPT1, CPT2 and CPT6 were shown to be localised to the plastids (Akhtar *et al*., 2013), but no short‐chain TPT has yet been localised to a subcellular compartment. We therefore endeavoured to determine the subcellular localisation of the all TPS and relevant TPT proteins. Sequence alignment showed that proteins in the TPS‐a clade have a short N‐terminal extension (9–18 residues) upstream the conserved R(R,P)(x)_8_W motif, except for TPS36 (48 residues) and TPS14 (27 residues) (Fig. S12). Most proteins in the TPS‐b clade and TPS‐g clade have a relatively long N‐terminal extension (28–56 residues), except for TPS38 (11 residues) (Fig. S13). Proteins in TPS‐e/f clade appear to have a transit peptide except for TPS46 (Fig. S14). TPS40 and TPS41 in TPS‐c clade both have a long N‐terminal extension (Fig. S7). All TPT proteins seem to have a transit peptide (Fig. S15).

**Figure 7:**
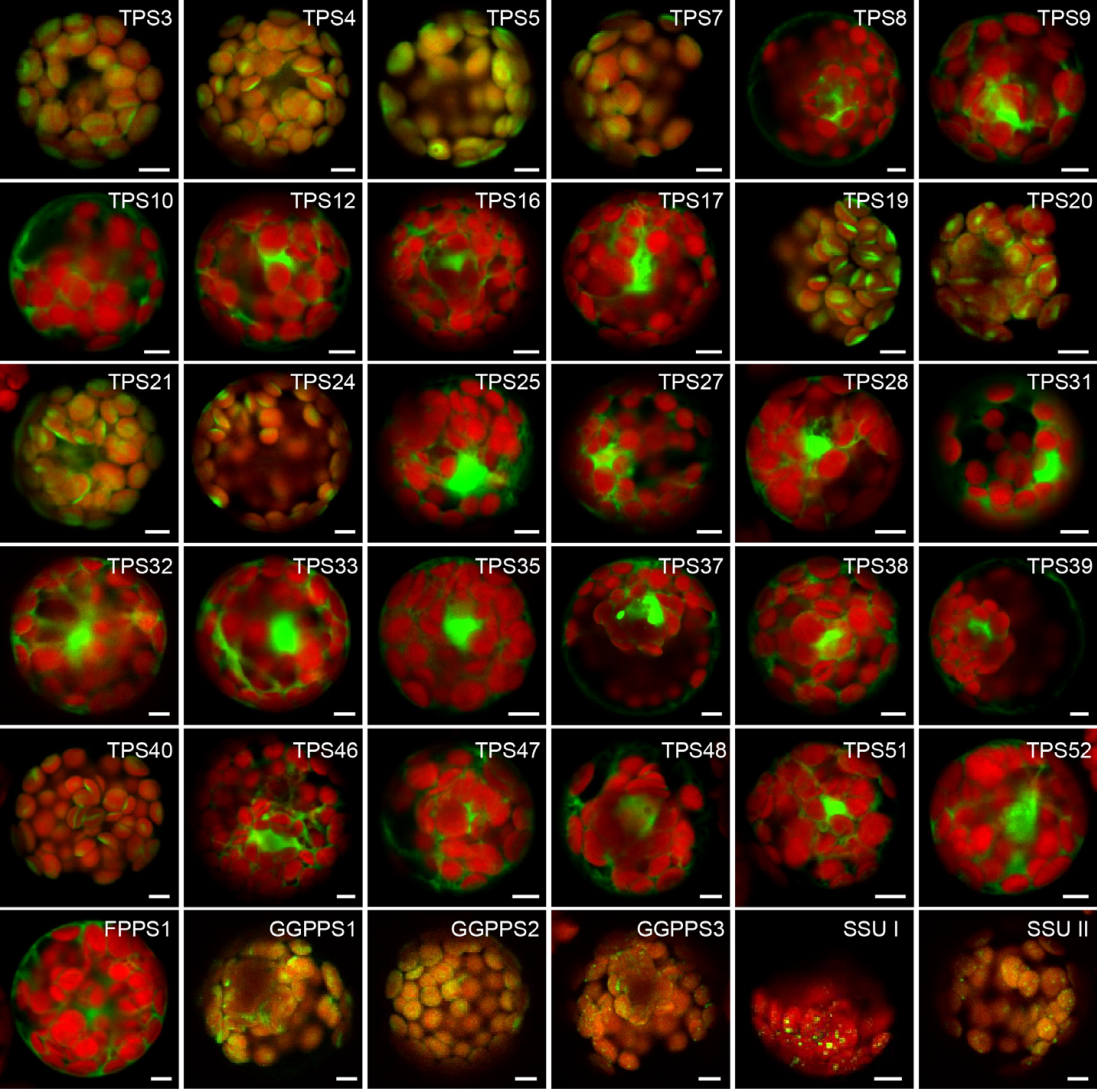
Subcellular localisation of the tomato terpene synthase (TPS) and *trans*‐prenyltransferase (TPT) proteins. The complete open reading frames of TPTs (bottom line) and the first *c*. 120 codons of TPSs were fused to a downstream GFP and transiently expressed in Arabidopsis leaf protoplasts. GFP fluorescence indicates the location of each fusion protein (shown in green) and the location of chloroplasts was determined by chlorophyll autofluorescence (shown in red), pictures shown are the merged channel. Bars, 5 μm. FPPS, farnesyl diphosphate synthase; GGPPS, geranylgeranyl diphosphate synthase; SSU, small subunit of geranyl diphosphate synthase.

**Figure 8:**
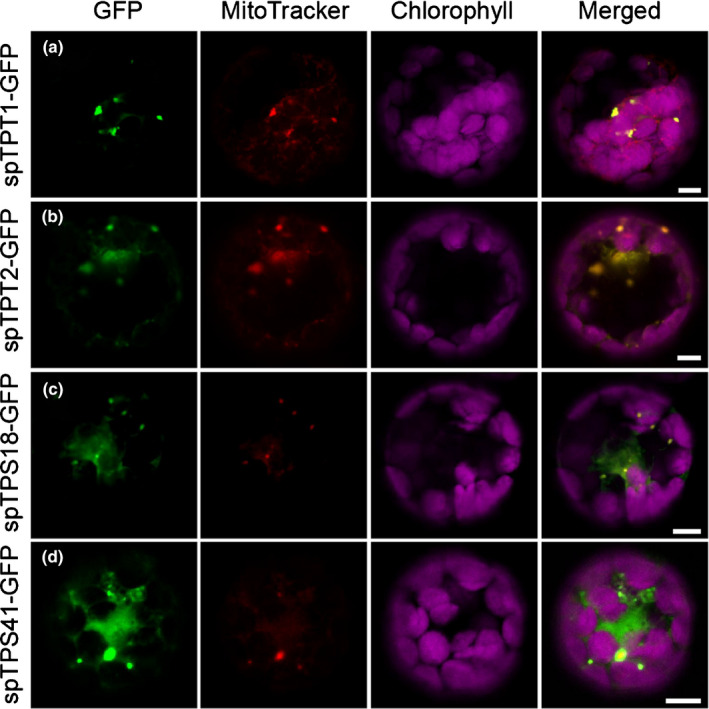
Subcellular localisation of the four mitochondrial proteins. (a, b) Localisation of *trans*‐prenyltransferase (TPT) 1 and 2, respectively. (c, d) Localisation of terpene synthase (TPS) 18 and 41, respectively. Sequences corresponding to the putative mitochondria transit peptide (sp) were fused to a downstream GFP and transiently expressed in Arabidopsis leaf protoplasts. GFP fluorescence indicates the location of each fusion protein (shown in green), the location of chloroplasts was determined by chlorophyll autofluorescence (shown in magenta), and the localisation of mitochondria was determined by the fluorescence of MitoTracker (shown in red). Column labelled ‘Merged’ represents all the combined fluorescent signals. Bars, 5 μm.

To experimentally determine their subcellular localisations, the part of each gene encoding the *c*. 120 amino acids of the N‐terminus was fused to the N‐terminus of GFP and transiently expressed in Arabidopsis mesophyll protoplasts. Consistent with predictions made by TargetP and ChloroP (Fig. S8), GFP fusion proteins of TPS3, TPS4, TPS5, TPS7, TPS19, TPS20, TPS21, TPS24, TPS40, GGPPS1, GGPPS2, GGPPS3 and SSU II were localised in the plastids, while GFP‐fusions of TPS8, TPS9, TPS10, TPS12, TPS16, TPS17, TPS25, TPS27, TPS28, TPS31, TPS32, TPS33, TPS35, TPS38, TPS39, TPS46, TPS47, TPS48, TPS51, TPS52 and FPPS1 were localised in the cytosol (Fig. 7). TPS18, TPS41, TPT1 and TPT2 were targeted to mitochondria as predicted by TargetP (Figs 8, S8a). TPS37 was predicted to be localised in the plastids by both TargetP and ChloroP (Fig. S8), however fluorescence signal of TPS37‐GFP fusion protein was only observed in the nonorganellar area of the cell, presumably the cytosol (Fig. 7). SSU I was targeted to plastids instead of the predicted cytosol localisation (Fig. 7). Taken together, three of the tomato TPS proteins are localised in the mitochondria, nine of them are targeted to the plastids and all the rest reside in the cytosol (Figs 7, 8). Analysis of the TPTs relevant to the production of TPS substrates indicates that FPPS1 is localised in the cytosol, while GGPPS1, GGPPS2, GGPPS3, SSU I and SSU II are localised in the plastids (Fig. 7). The speckled fluorescence pattern of GGPPS3, SSU I and SSU II‐GFP fusion proteins inside the plastid also suggests a suborganelle localisation, presumably the thylakoid, as reported in other plants (Zhou *et al*., 2017; Wang *et al*., 2018). It must be noted that these results, obtained in Arabidopsis protoplasts, will need to be confirmed in the future using tomato cells.

### Expression of TPS and prenyltransferase genes and metabolic profiling of terpenes

**Figure 9:**
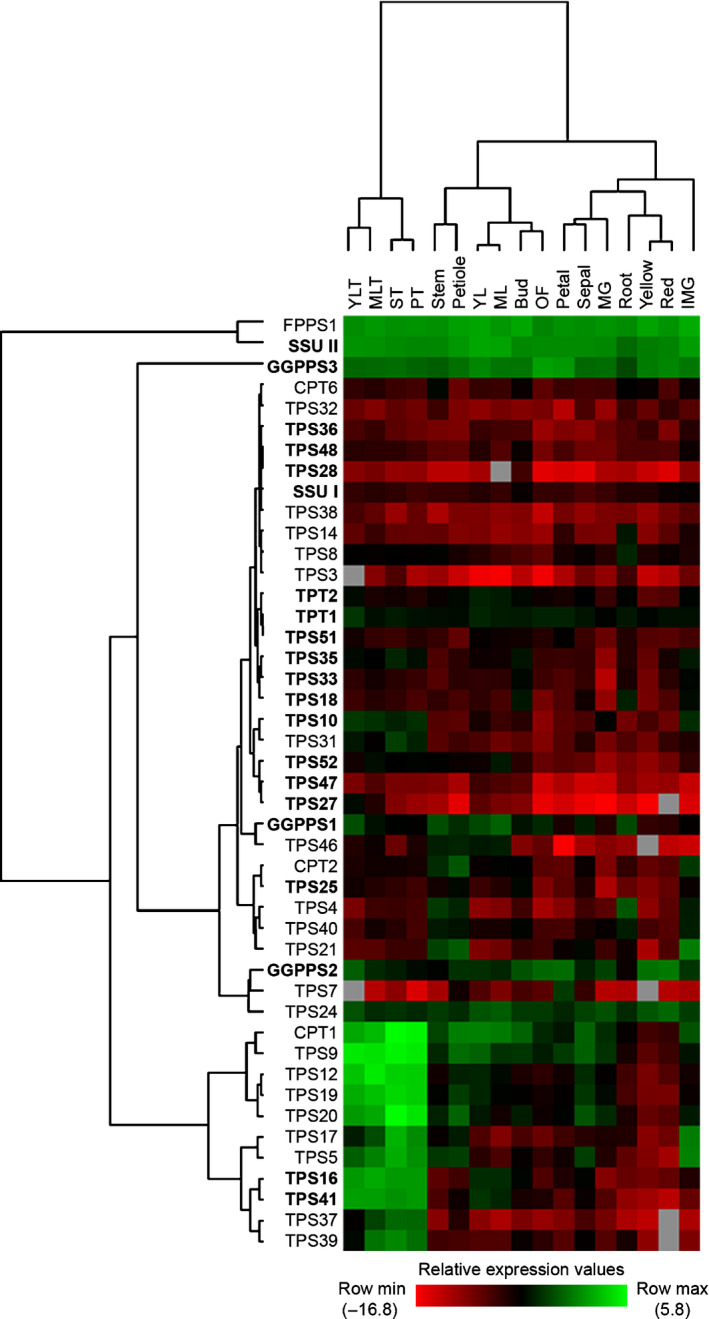
Gene expression analysis of terpene metabolism‐related genes. Heat map showing the relative transcript abundance of terpene synthase and prenyltransferase genes from tomato plant tissues taken at different developmental stages. Genes functionally characterised in this work are shown in bold. The values in the colour bar are log‐transformed values of relative transcript levels (2^−∆Ct^) determined by quantitative real‐time PCR (RT‐qPCR), grey colour indicates missing of transcript reads. The hierarchical clustering was performed using average linkage method by cluster 3.0. Abbreviations for tissues: IMG, immature green fruit; MG, mature green fruit; ML, mature leaf; MLT, mature leaf trichome; OF, fully opened flower; PT, petiole trichome; ST, stem trichome; YL, young leaf; YLT, young leaf trichome. Abbreviations for genes: CPT, *cis*‐prenyltransferase; FPPS, farnesyl diphosphate synthase; GGPPS, geranylgeranyl diphosphate synthase; SSU, small subunit of geranyl diphosphate synthase; TPS, terpene synthase; TPT, *trans*‐prenyltransferase.

**Figure 10:**
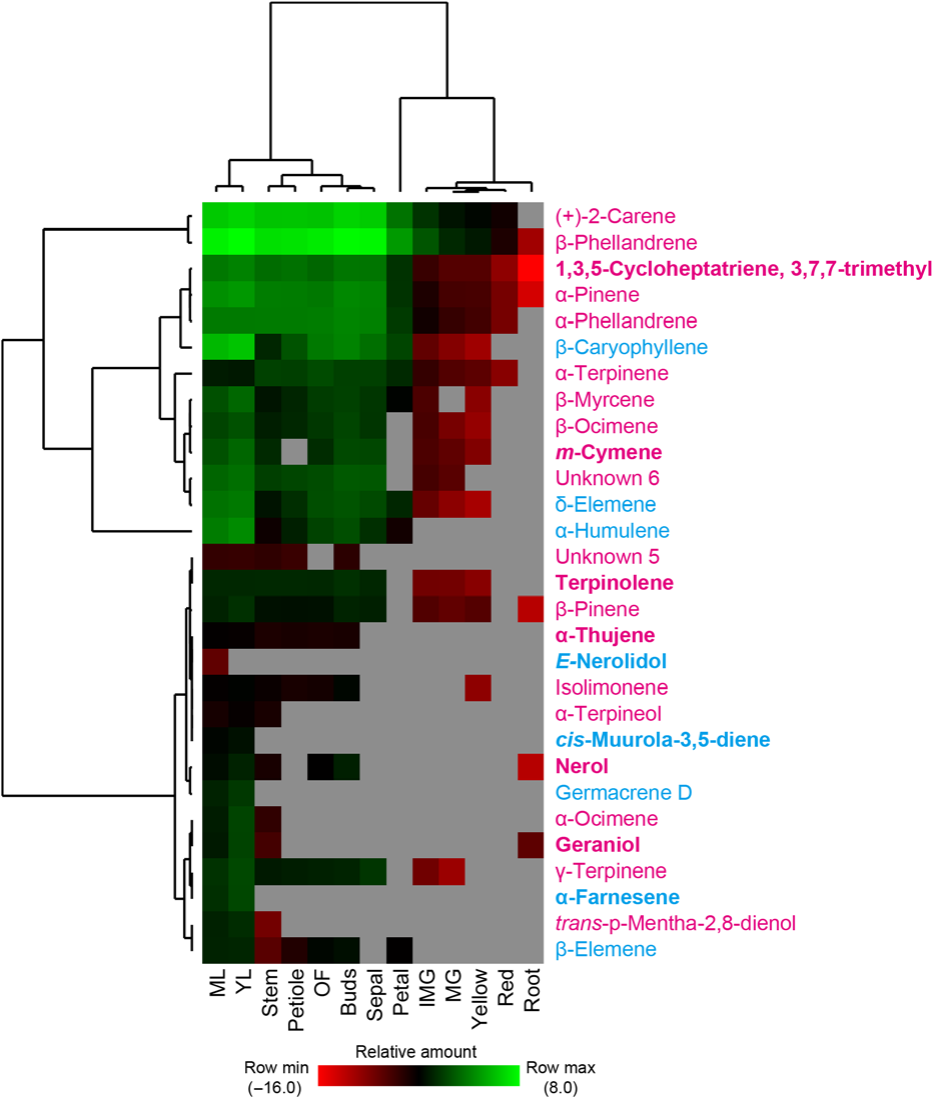
Terpene profiles of different tomato tissues. The peak areas normalised to the internal standard and tissue wet weight were calculated for each of the 29 terpene signals in 13 tomato tissues. Mass spectra and retention indices of the terpene products can be found in Supporting Information Fig. S3; Table S2, respectively. The average from two to three replicates was calculated and log‐transformed for the construction of heatmap. Grey colour indicates undetectable. The hierarchical clustering was performed using average linkage method by cluster 3.0. Monoterpenes are shown in magenta and sesquiterpenes are shown in blue. New terpenes identified in this work are shown in bold. IMG, immature green fruit; MG, mature green fruit; ML, mature leaf; OF, fully opened flower; YL, young leaf.

To determine the relative amount and tissue distribution of all the TPS transcripts as well as TPT and *cis*‐prenyltransferase transcripts encoding the enzymes that form the substrates of TPS enzymes, the expression of each gene was measured in a total of 17 tomato tissues using quantitative real‐time PCR (RT‐qPCR) with gene‐specific primers. Expression was quantified and is presented as a heat map (Fig. 9) to compare the relative expression levels for each gene in different tissues. To identify any correlation between TPS and prenyltransferase gene expression and terpene profiles, terpene volatiles in these tissues were analysed as well (Figs 10, S16). Transcripts of the four new TPS genes (*TPS47*, *TPS48*, *TPS51* and *TPS52*) are present at low levels in multiple tissues, with slightly higher amounts observed in young leaf, flower bud, petal and mature leaf, respectively (Fig. 9). Among the other previously functionally uncharacterised TPS genes, transcripts of *TPS16* and *TPS41* are almost exclusively found in various trichomes. *TPS10* transcripts are enriched in immature green fruit in addition to trichomes, while *TPS25* is expressed in multiple tissues primarily in petiole and flower bud, *TPS27* is maximally expressed in young leaf trichomes, and *TPS28* is mainly expressed in flower bud at low levels. *TPS18* is mostly expressed in roots and flower buds, *TPS36* is mainly expressed in mature leaf trichomes and immature green and yellow fruits. *TPS33* is primarily expressed in immature green fruit and flower bud, while *TPS35* is mainly expressed in stem trichomes, flower bud and immature green fruit (Fig. 9).

The TPT genes *FPPS1*, *SSU II* and *GGPPS3* are highly expressed in all 17 tissues, *GGPPS1* is mostly expressed in leaf tissues, *GGPPS2* is mainly expressed in fruits and flower parts, and *SSU I* is mainly expressed in flower bud and fruits (Fig. 9). Our results for CPTs were similar to those previously reported (Akhtar *et al*., 2013; Matsuba *et al*., 2013), with the highest expression of *CPT1* found in trichomes, lower expression of *CPT2* mostly in petiole and stem, and lowest expression of *CPT6* mainly in stem and root tissues (Fig. 9).

**Figure 11:**
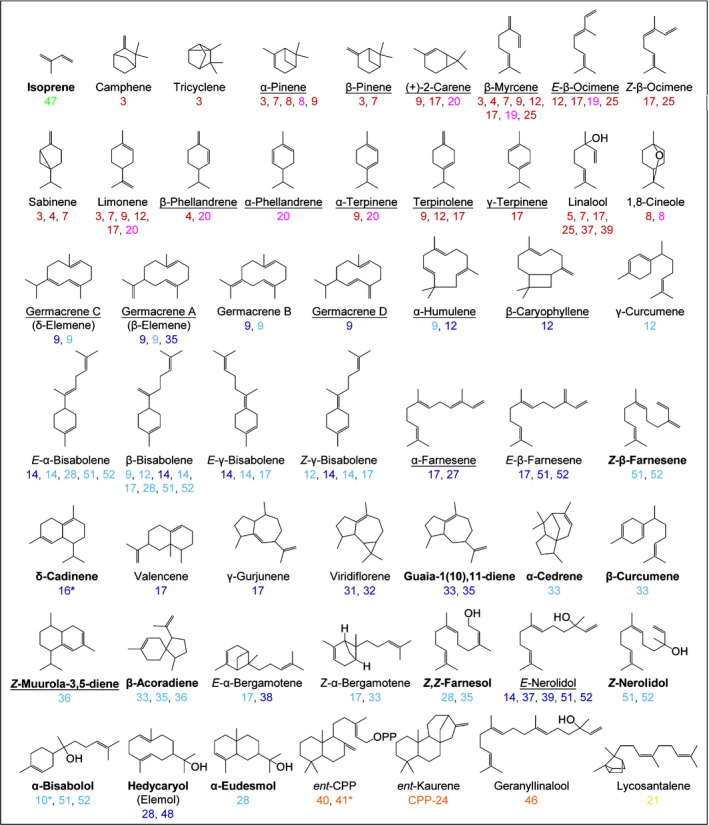
Terpene skeletons formed by the 34 functional tomato terpene synthases. New enzymatic terpene products reported in this study are shown in bold, the corresponding terpene synthase (TPS) enzymes are showing beneath each compound with different colours: light green, dimethylallyl diphosphate (DMAPP) as substrate; dark red, geranyl diphosphate (GPP) as substrate; magenta, neryl diphosphate (NPP) as substrate; dark blue, *trans*‐farnesyl diphosphate (*E*,*E*‐FPP) as substrate; light blue, *cis*‐farnesyl diphosphate (*Z*,*Z*‐FPP) as substrate; orange, geranylgeranyl diphosphate (GGPP) as substrate; yellow, nerylneryl diphosphate (NNPP) as substrate. Enzymes characterised by transiently expressing in tobacco are indicated with asterisks, all other enzymes are characterised by *in vitro* enzyme assays (detailed in Supporting Information Table S6). Elemol is the thermal breakdown product of (+)‐hedycaryol; β‐elemene and δ‐elemene are the thermal rearrangements of germacrene A and germacrene C, respectively. Terpenes detected from tomato tissues are underlined.

Our metabolic profiling of multiple tomato tissues detected a total of 21 monoterpenes and eight sesquiterpenes (Figs 10, S3; Table S2). In addition to the abundant and previously reported monoterpenes (+)‐2‐carene and β‐phellandrene and the sesquiterpenes β‐caryophyllene and α‐humulene (Schilmiller *et al*., 2009; Falara *et al*., 2011), we also identified several new terpenes (Fig. 10). Particularly noteworthy are α‐farnesene and *cis*‐muurola‐3,5‐diene, detected only from leaf tissues (Fig. 10), positively correlated with the expression patterns of *TPS27* (α‐farnesene synthase) and *TPS36* (*cis*‐muurola‐3,5‐diene synthase) (Fig. S17), which are responsible for their synthesis respectively (Figs 3, 11; Table S6).

## Discussion

### The arrangement of TPS genes in the tomato genome

The majority of the 34 functional tomato TPS genes are located in clusters on chromosomes 1, 2, 6, 7, 8 and 10, while the other TPS genes are located on chromosomes 3, 4, 9 and 12 without another TPS genes nearby (Fig. 1). Examination of previously performed plantiSMASH analysis of the tomato genome (Kautsar *et al*., 2017) identified eight gene clusters related to terpene metabolism (Fig. S18). The clusters on chromosomes 1 and 2 consist of only TPS genes, while the other clusters also contain putative cytochrome P450s, methyltransferases, acyltransferases and glycosyltransferases (Fig. S18). Potential modifications of the direct terpene products (Fig. 11) by these enzymes may lead to the formation of new terpene skeletons with new biological functions, and will need further investigation.

### Evolution of the TPS family

While there are still seven remaining enzymes in the Arabidopsis TPS family that have not yet been biochemically characterised (a quarter of the members) and subcellular localisations from many of the Arabidopsis TPS proteins have not yet been examined experimentally (Table S7), a comparison of the TPS families from tomato and Arabidopsis affords unprecedented and illuminating view of the dynamic evolution of the terpene synthase family. However, the fact that these two angiospermous species are both eudicots means that the generality of any conclusion drawn from such a comparison must await confirmation from additional comparisons taken among more distantly related land plants. With this caveat, we note that while the number of functional TPS genes in both dicot species is similar (Tables S3, S7), this similarity is misleading, masking major changes that have occurred in the TPS family in the two lineages since they last shared a common ancestor.

**Figure 12:**
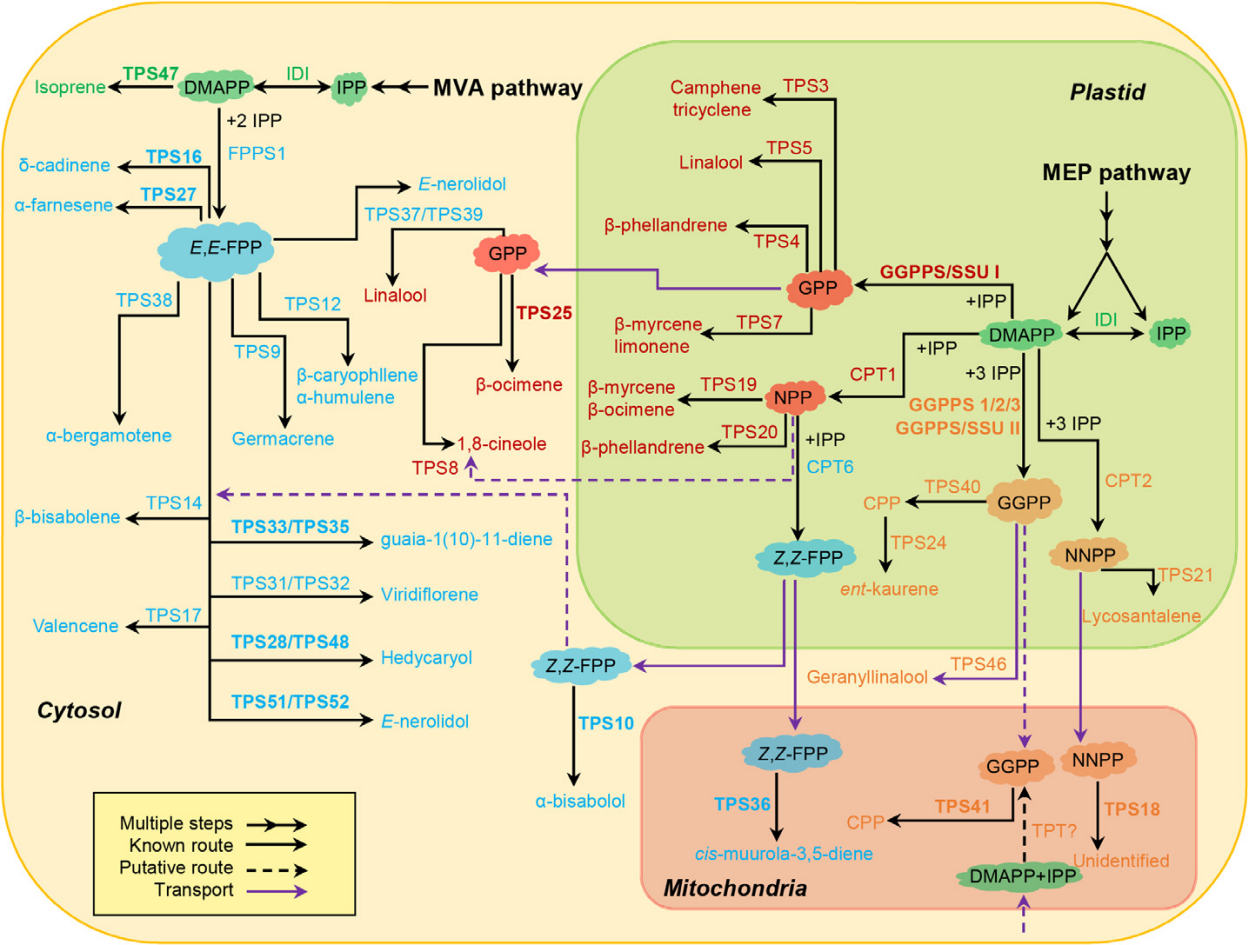
Terpene biosynthesis pathway in tomato. Terpene precursors are synthesised by the cytosolic mevalonic acid (MVA) pathway and the plastidic methylerythritol phosphate (MEP) pathway. The prenyl diphosphate substrates are labelled with clouds. C_5_, C_10_, C_15_, C_20_ compounds and their related enzymes are shown in green, red, light blue and orange, respectively. Enzymes characterised in this work are shown in bold. CPP, copalyl diphosphate; CPT, *cis*‐prenyltransferase; DMAPP, dimethylallyl diphosphate; *E*,*E*‐FPP, *trans*‐farnesyl diphosphate; FPPS, *E*,*E*‐FPP synthase; GPP, geranyl diphosphate; GGPP, geranylgeranyl diphosphate; GGPPS, GGPP synthase; IDI, isopentenyl diphosphate isomerase; IPP, isopentenyl diphosphate; NPP, neryl diphosphate; NNPP, nerylneryl diphosphate; SSU, small subunit of GGPPS; TPS, terpene synthase; TPT, *trans*‐prenyltransferase; *Z*,*Z*‐FPP, *cis*‐farnesyl diphosphate.

There are similar numbers of members in the TPS‐a clade in tomato and Arabidopsis – 15 and 22, respectively – but the phylogenetic analysis makes clear that the two species shared only one common TPS‐a ancestor from which each species evolved, by gene duplications, its extant group of TPS‐a genes (Fig. 2). This observation almost surely means that each lineage must have also lost TPS‐a genes along the way, and the presence of many TPS pseudogenes in both genomes (Table S4; Aubourg *et al*., 2002) lends strong support to this supposition. More interesting, while multiple gene duplications occurred in the TPS‐a clade in both lineages, the process of functional divergence of the duplicated genes appears to be quite different in the two lineages. In tomato, all 15 TPS‐a genes encode sesquiterpene synthases, and all but one of these enzymes (TPS36) are localised to the cytosol (Figs 2, 7, 12). However, they have clearly diverged in the type of products they produce, and also in the type of substrate they use; some use only *E*,*E*‐FPP, some can also use *Z*,*Z*‐FPP, and some use *Z*,*Z*‐FPP exclusively (Fig. 2). By contrast, only four of the 22 Arabidopsis TPS‐a genes are cytosolic sesquiterpene synthases. The rest are plastidic and/or mitochondrial diterpene synthases, plastidic sesterterpene synthases and one is a mitochondrial sesquiterpene synthase, the same as tomato TPS36 (seven of the Arabidopsis TPS‐a enzymes are still not functionally characterised; Fig. 2).

Regarding the TPS‐b clade, the Arabidopsis genome is missing (Fig. 2) representatives from a deep branch called the ‘β‐ocimene synthase branch’ to which all known isoprene synthases belong (Sharkey *et al*., 2013). There are three members in this branch in the tomato genome, one of which, *TPS47*, encodes a cytosolic isoprene synthase (all other known isoprene synthases are plastidic; Sharkey *et al*., 2013). The other two members, *TPS25* and *TPS27*, encode cytosolic monoterpene and sesquiterpene synthases, respectively (Figs 3, 5, 7). The second branch of the TPS‐b clade contains six genes in both tomato and Arabidopsis (Fig. 2), and again the phylogenetic analysis indicates that both species had only a single common TPS gene ancestor of this branch, and that the six genes of this branch in each species are the result of more recent gene duplications (and loss). While most of these genes in each species encode plastidic monoterpene synthases, a cytosolic TPS‐b C_10_/C_15_ synthase evolved in this group in the Arabidopsis lineage, while in the tomato lineage this group also contains one cytosolic monoterpene synthase and one cytosolic sesquiterpene synthase (Fig. 2).

The TPS‐g clade is present in both genomes, with one representative – a plastidic monoterpene synthase – in Arabidopsis, and two representatives – both cytosolic C_10_/C_15_ synthases – in tomato (Fig. 2). Similarly, there is one Arabidopsis gene in the TPS‐c clade, a *bona fide* plastidic CPS, while tomato has two CPS genes, *TPS40* and *TPS41* (Fig. 2). TPS40 is plastidic (Fig. 7) and was shown to be involved in gibberellin biosynthesis (Rebers *et al*., 1999; Falara *et al*., 2011). TPS41 is localised to the mitochondria (Fig. 8d) and its physiological role is presently unknown. It should be pointed out that *N. tabacum* has at least one additional member in the TPS‐c clade besides its *bona fide* CPS, but this additional enzyme has been shown to produce 8‐hydroxy copalyl diphosphate (rather than CPP) that is the substrate of abienol synthase (Sallaud *et al*., 2012).

The TPS‐e/f clade in general contains two deep branches that diverged before the split of the gymnosperm/angiosperm lineages (Fig. 2), one encoding a plastidic KS (Falara *et al*., 2011) and one encoding a cytosolic geranyllinalool synthase (GLS) (Falara *et al*., 2014). The enzymes encoded by both genes use GGPP as the substrate (Fig. 2). There seems to have been little evolutionary novelty in this clade in the Arabidopsis genome. It contains a single GLS gene and a single KS gene (Fig. 2; Table S7). Tomato also contains a single GLS gene and a single KS gene (Fig. 2; Table S3). However, as previously shown (Matsuba *et al*., 2013), the tomato genome also contains four TPS‐e/f genes in a clade that diverged from KS before the split of the Arabidopsis and tomato lineages, two of which encode plastidic monoterpene synthases using NPP as the substrate, and a third one encoding a plastidic diterpene synthase whose substrate is NNPP (Fig. 2). Here we show that the fourth gene in this group, *TPS18*, encodes a mitochondrial diterpene synthase that uses NNPP (Figs 4b, 8c). While the functions of these four genes are likely to have evolved recently, the orthologue of these genes must have been lost in the lineage leading to Arabidopsis.

### Biosynthesis of different classes of short‐chain terpenes in different subcellular compartments

Early analysis of the subcellular distribution of short‐chain (C_10_–C_25_) *trans*‐prenyltransferases indicated that GPPS and GGPPS are localised in the plastid, while *E*,*E*‐FPPS is localised mostly to the cytosol and perhaps to the mitochondria as well (Sun *et al*., 2016). This led to the general assumption that monoterpenes and diterpenes are synthesised in the plastids, while sesquiterpenes are synthesised in the cytosol (McGarvey & Croteau, 1995). Short‐chain CPTs, which provide substrates to some TPSs in *Solanum*, were shown to be all localised to the plastids (Akhtar *et al*., 2013), and the mono‐ and diterpene synthases that were previously shown to use these substrates were also localised to the plastids, thus providing no exception to the rule. However, *Z*,*Z*‐FPPS was also localised to the plastid, and a plastidic TPS‐e/f enzyme capable of using *Z*,*Z*‐FPP to produce sesquiterpenes was discovered in *S. harbochaites* (Sallaud *et al*., 2009).

In the last decade, there have been additional examples of rule‐breaking TPSs. GLS is a cytosolic diterpene synthase in both Arabidopsis (Herde *et al*., 2008) and tomato (Fig. 7). A cytosolic monoterpene synthase, geraniol synthase, from *Lippia dulcis* was shown to produce geraniol from GPP *in planta* (Dong *et al*., 2013), a cytosolic C_10_/C_15_ synthase was shown to use GPP to produce the monoterpene linalool in strawberry (Aharoni *et al*., 2004), and a basil cytosolic C_10_/C_15_ synthase was shown to use GPP to produce monoterpenes in a tomato heterologous system (Gutensohn *et al*., 2013). Although a cytosolic GGPPS has now been molecularly identified from several species (Coman *et al*., 2014), a cytosolic GPPS has not, although there are reports of cytosolic localisation of GPPS activity in the roots of *Lithospermum erythrorhizon* (Sommer *et al*., 1995). Additionally, accumulating results have led to the realisation that plant cells have mechanisms for transport of various prenyl diphosphates between compartments. The observations presented here indicate that more than a few tomato TPS enzymes are present in subcellular compartments in which their substrate may *not* be synthesised (Fig. 12). To highlight one example, mitochondrial TPS36 used *Z*,*Z*‐FPP to catalyse the formation of *cis*‐muurola‐3,5‐diene (Fig. 3b), a sesquiterpene that is not produced by any other tomato TPS enzyme (Fig. 11) but can be detected from tomato leaves (Fig. 10), suggesting that *Z*,*Z*‐FPP can also be imported into mitochondria from plastid (Fig. 12). Taken together, it appears that any class of short‐chain terpenes can no longer be assumed to be limited to a specific cellular compartment (Fig. 12).

### Conclusions

The analysis of the biochemical activities and subcellular localisations of all the enzymes encoded by the functional TPS genes in tomato and of the genes encoding the enzymes that synthesise the substrates of terpene synthases provides an unprecedented view of the complexity of the terpenoid metabolic network. The terpenes directly produced by tomato TPS genes, and those that are obtained after modification of the direct products by additional enzymes, some of which may be encoded by genes clustered together with TPS genes, participate in multiple processes in the plants. Some have well established physiological roles such as hormones (e.g. gibberellins). The roles of others may be hypothesised to be ecological in nature, such as defence against insects or microorganisms, attraction of beneficial organisms, or even interaction with other plants (Pichersky & Raguso, 2018). The complete functional elucidation of the tomato TPS family now allows such roles to be investigated in detail.

## Author contributions

EP and FZ designed the research. FZ performed all the experiments. EP and FZ interpreted the results and wrote the paper.

## Supporting information

Please note: Wiley Blackwell are not responsible for the content or functionality of any Supporting Information supplied by the authors. Any queries (other than missing material) should be directed to the *New Phytologist* Central Office.

**Fig. S1** Annotated sequences of TPS51 and TPS52.
**Fig. S2** SDS‐PAGE analysis of the purified recombinant His‐tagged TPS proteins.
**Fig. S3** Mass spectra of identified terpenes from Figs 3–5 & 10.
**Fig. S4** GC–MS analysis of elemol and (+)‐hedycaryol.
**Fig. S5** GC–MS analysis of the products formed in planta by transiently co‐expressing TPS with CPT genes in *Nicotiana benthamiana* leaves.
**Fig. S6** Analysis of tomato terpene synthases with isoprene synthases from other plants.
**Fig. S7** Sequence alignment of the proteins encoded by the functional tomato TPS‐c genes and CPS genes from other plants.
**Fig. S8** Predictions of subcellular localisation for TPS and prenyltransferase proteins made by TargetP 1.1 and ChloroP 1.1.
**Fig. S9** Phylogenetic analysis of tomato TPT homologues and TPTs identified in other plants.
**Fig. S10** GC–MS analysis of the products formed in planta by transiently co‐expressing TPS with TPT genes in *Nicotiana benthamiana* leaves.
**Fig. S11** Sequence alignment of five Arabidopsis sesterterpene synthases and tomato TPSs from TPS‐a, TPS‐b and TPS‐g clades.
**Fig. S12** Sequence alignment of the proteins encoded by the functional tomato TPS‐a genes.
**Fig. S13** Sequence alignment of the proteins encoded by the functional tomato TPS‐b and TPS‐g genes.
**Fig. S14** Sequence alignment of the proteins encoded by the functional tomato TPS‐e/f genes.
**Fig. S15** Sequence analysis of TPT homologues from tomato and other plants.
**Fig. S16** GC–MS analysis of terpenes extracted from tomato leaves.
**Fig. S17** Correlation analysis of terpene biosynthesis genes and volatile terpenes from tomato.
**Fig. S18** Terpene biosynthesis gene clusters identified from tomato genome by plantiSMASH.
**Methods S1** Details on experimental procedures.
**Table S1** Primers used in this study.
**Table S2** A list of terpene compounds identified from Figs 3–5 & 10.
**Table S3** A list of tomato terpene synthase genes, their characteristics and the enzymes they encode.
**Table S4** A list of tomato terpene synthase pseudogenes.
**Table S5** A list of the *trans*‐prenyltransferase genes in tomato.
**Table S6** A list of terpene compounds produced by all the functional tomato terpene synthases.
**Table S7** A list of Arabidopsis terpene synthase genes, their characteristics and the enzymes they encode.

## References

[nph16431-bib-0001] Aharoni A , Giri AP , Verstappen FWA , Bertea CM , Sevenier R , Sun ZK , Jongsma MA , Schwab W , Bouwmeester HJ . 2004 Gain and loss of fruit flavor compounds produced by wild and cultivated strawberry species. Plant Cell 16: 3110–3131. 15522848 10.1105/tpc.104.023895 PMC527202

[nph16431-bib-0002] Akhtar TA , Matsuba Y , Schauvinhold I , Yu G , Lees HA , Klein SE , Pichersky E . 2013 The tomato *cis*‐prenyltransferase gene family. The Plant Journal 73: 640–652. 23134568 10.1111/tpj.12063

[nph16431-bib-0003] Alquézar B , Rodríguez A , de la Peña M , Peña L . 2017 Genomic analysis of terpene synthase family and functional characterization of seven sesquiterpene synthases from *Citrus sinensis* . Frontiers in Plant Science 8: 1481. 28883829 10.3389/fpls.2017.01481 PMC5573811

[nph16431-bib-0004] Ament K , Van Schie CC , Bouwmeester HJ , Haring MA , Schuurink RC . 2006 Induction of a leaf specific geranylgeranyl pyrophosphate synthase and emission of (*E*, E)‐4,8,12‐trimethyltrideca‐1,3,7,11‐tetraene in tomato are dependent on both jasmonic acid and salicylic acid signaling pathways. Planta 224: 1197–1208. 16786318 10.1007/s00425-006-0301-5

[nph16431-bib-0005] Aubourg S , Lecharny A , Bohlmann J . 2002 Genomic analysis of the terpenoid synthase (AtTPS) gene family of *Arabidopsis thaliana* . Molecular Genetics and Genomics 267: 730–745. 12207221 10.1007/s00438-002-0709-y

[nph16431-bib-0006] Bensen RJ , Zeevaart JAD . 1990 Comparison of *ent*‐kaurene synthetase A‐activity and B‐activity in cell‐free‐extracts from young tomato fruits of wild‐type and *gib‐1*, *gib‐2*, and *gib‐3* tomato plants. Journal of Plant Growth Regulation 9: 237–242.

[nph16431-bib-0007] Bleeker PM , Spyropoulou EA , Diergaarde PJ , Volpin H , De Both MT , Zerbe P , Bohlmann J , Falara V , Matsuba Y , Pichersky E *et al* 2011 RNA‐seq discovery, functional characterization, and comparison of sesquiterpene synthases from *Solanum lycopersicum* and *Solanum habrochaites* trichomes. Plant Molecular Biology 77: 323–336. 21818683 10.1007/s11103-011-9813-x PMC3193516

[nph16431-bib-0008] Bohlmann J , Meyer‐Gauen G , Croteau R . 1998 Plant terpenoid synthases: molecular biology and phylogenetic analysis. Proceedings of the National Academy of Sciences, USA 95: 4126–4133. 10.1073/pnas.95.8.4126 PMC22453 9539701

[nph16431-bib-0009] Booth JK , Page JE , Bohlmann J . 2017 Terpene synthases from *Cannabis sativa* . PLoS ONE 12: e0173911. 28355238 10.1371/journal.pone.0173911 PMC5371325

[nph16431-bib-0010] Boutanaev AM , Moses T , Zi JC , Nelson DR , Mugford ST , Peters RJ , Osbourn A . 2015 Investigation of terpene diversification across multiple sequenced plant genomes. Proceedings of the National Academy of Sciences, USA 112: E81–E88. 10.1073/pnas.1419547112 PMC4291660 25502595

[nph16431-bib-0011] Chen F , Ro DK , Petri J , Gershenzon J , Bohlmann J , Pichersky E , Tholl D . 2004 Characterization of a root‐specific Arabidopsis terpene synthase responsible for the formation of the volatile monoterpene 1,8‐cineole. Plant Physiology 135: 1956–1966. 15299125 10.1104/pp.104.044388 PMC520767

[nph16431-bib-0012] Chen F , Tholl D , Bohlmann J , Pichersky E . 2011 The family of terpene synthases in plants: a mid‐size family of genes for specialized metabolism that is highly diversified throughout the kingdom. The Plant Journal 66: 212–229. 21443633 10.1111/j.1365-313X.2011.04520.x

[nph16431-bib-0013] Chen F , Tholl D , D'Auria JC , Farooq A , Pichersky E , Gershenzon J . 2003 Biosynthesis and emission of terpenoid volatiles from Arabidopsis flowers. Plant Cell 15: 481–494. 12566586 10.1105/tpc.007989 PMC141215

[nph16431-bib-0014] Chen QW , Jiang T , Liu YX , Liu HL , Zhao T , Liu ZX , Gan XC , Hallab A , Wang XM , He J *et al* 2019 Recently duplicated sesterterpene (C25) gene clusters in *Arabidopsis thaliana* modulate root microbiota. Science China Life Sciences 62: 947–958. 31079337 10.1007/s11427-019-9521-2

[nph16431-bib-0015] Christianson DW . 2017 Structural and chemical biology of terpenoid cyclases. Chemical Reviews 117: 11570–11648. 28841019 10.1021/acs.chemrev.7b00287 PMC5599884

[nph16431-bib-0016] Colby SM , Crock J , Dowdle‐Rizzo B , Lemaux PG , Croteau R . 1998 Germacrene C synthase from *Lycopersicon esculentum* cv. VFNT cherry tomato: cDNA isolation, characterization, and bacterial expression of the multiple product sesquiterpene cyclase. Proceedings of the National Academy of Sciences, USA 95: 2216–2221. 10.1073/pnas.95.5.2216 PMC19298 9482865

[nph16431-bib-0017] Coman D , Altenhoff A , Zoller S , Gruissem W , Vranová E . 2014 Distinct evolutionary strategies in the GGPPS family from plants. Frontiers in Plant Science 5: 230. 24904625 10.3389/fpls.2014.00230 PMC4034038

[nph16431-bib-0018] Dong LM , Miettinen K , Goedbloed M , Verstappen FWA , Voster A , Jongsma MA , Memelink J , van der Krol S , Bouwmeester HJ . 2013 Characterization of two geraniol synthases from *Valeriana officinalis* and *Lippia dulcis*: Similar activity but difference in subcellular localization. Metabolic Engineering 20: 198–211. 24060453 10.1016/j.ymben.2013.09.002

[nph16431-bib-0019] Dudareva N , Martin D , Kish CM , Kolosova N , Gorenstein N , Fäldt J , Miller B , Bohlmann J . 2003 (*E*)‐beta‐ocimene and myrcene synthase genes of floral scent biosynthesis in snapdragon: function and expression of three terpene synthase genes of a new terpene synthase subfamily. Plant Cell 15: 1227–1241. 12724546 10.1105/tpc.011015 PMC153728

[nph16431-bib-0020] Dudareva N , Raguso RA , Wang J , Ross JR , Pichersky E . 1998 Floral scent production in *Clarkia breweri*. III. Enzymatic synthesis and emission of benzenoid esters. Plant Physiology 116: 599–604. 9489012 10.1104/pp.116.2.599 PMC35117

[nph16431-bib-0021] Falara V , Akhtar TA , Nguyen TT , Spyropoulou EA , Bleeker PM , Schauvinhold I , Matsuba Y , Bonini ME , Schilmiller AL , Last RL *et al* 2011 The tomato terpene synthase gene family. Plant Physiology 157: 770–789. 21813655 10.1104/pp.111.179648 PMC3192577

[nph16431-bib-0022] Falara V , Alba JM , Kant MR , Schuurink RC , Pichersky E . 2014 Geranyllinalool synthases in Solanaceae and other angiosperms constitute an ancient branch of diterpene synthases involved in the synthesis of defensive compounds. Plant Physiology 166: 428–441. 25052853 10.1104/pp.114.243246 PMC4149726

[nph16431-bib-0023] Gaffe J , Bru JP , Causse M , Vidal A , Stamitti‐Bert L , Carde JP , Gallusci P . 2000 *LeFPS1*, a tomato farnesyl pyrophosphate gene highly expressed during early fruit development. Plant Physiology 123: 1351–1362. 10938353 10.1104/pp.123.4.1351 PMC59093

[nph16431-bib-0024] Gao Y , Honzatko RB , Peters RJ . 2012 Terpenoid synthase structures: a so far incomplete view of complex catalysis. Natural Product Reports 29: 1153–1175. 22907771 10.1039/c2np20059g PMC3448952

[nph16431-bib-0025] Gershenzon J , Dudareva N . 2007 The function of terpene natural products in the natural world. Nature Chemical Biology 3: 408–414. 17576428 10.1038/nchembio.2007.5

[nph16431-bib-0026] Gutensohn M , Orlova I , Nguyen TT , Davidovich‐Rikanati R , Ferruzzi MG , Sitrit Y , Lewinsohn E , Pichersky E , Dudareva N . 2013 Cytosolic monoterpene biosynthesis is supported by plastid‐generated geranyl diphosphate substrate in transgenic tomato fruits. The Plant Journal 75: 351–363. 23607888 10.1111/tpj.12212

[nph16431-bib-0027] Hattan J , Shindo K , Ito T , Shibuya Y , Watanabe A , Tagaki C , Ohno F , Sasaki T , Ishii J , Kondo A *et al* 2016 Identification of a novel hedycaryol synthase gene isolated from *Camellia brevistyla* flowers and floral scent of *Camellia* cultivars. Planta 243: 959–972. 26744017 10.1007/s00425-015-2454-6

[nph16431-bib-0028] Hayashi K , Kawaide H , Notomi M , Sakigi Y , Matsuo A , Nozaki H . 2006 Identification and functional analysis of bifunctional *ent*‐kaurene synthase from the moss *Physcomitrella patens* . FEBS Letters 580: 6175–6181. 17064690 10.1016/j.febslet.2006.10.018

[nph16431-bib-0029] Herde M , Gärtner K , Köllner TG , Fode B , Boland W , Gershenzon J , Gatz C , Tholl D . 2008 Identification and regulation of TPS04/GES, an Arabidopsis geranyllinalool synthase catalyzing the first step in the formation of the insect‐induced volatile C_16_‐homoterpene TMTT. Plant Cell 20: 1152–1168. 18398052 10.1105/tpc.106.049478 PMC2390743

[nph16431-bib-0030] Huang AC , Kautsar SA , Hong YJ , Medema MH , Bond AD , Tantillo DJ , Osbourn A . 2017 Unearthing a sesterterpene biosynthetic repertoire in the Brassicaceae through genome mining reveals convergent evolution. Proceedings of the National Academy of Sciences, USA 114: E6005–E6014. 10.1073/pnas.1705567114 PMC5530694 28673978

[nph16431-bib-0031] Iijima Y , Davidovich‐Rikanati R , Fridman E , Gang DR , Bar E , Lewinsohn E , Pichersky E . 2004 The biochemical and molecular basis for the divergent patterns in the biosynthesis of terpenes and phenylpropenes in the peltate glands of three cultivars of basil. Plant Physiology 136: 3724–3736. 15516500 10.1104/pp.104.051318 PMC527170

[nph16431-bib-0032] Ilmén M , Oja M , Huuskonen A , Lee S , Ruohonen L , Jung S . 2015 Identification of novel isoprene synthases through genome mining and expression in *Escherichia coli* . Metabolic Engineering 31: 153–162. 26275749 10.1016/j.ymben.2015.08.001

[nph16431-bib-0033] Jones MO , Perez‐Fons L , Robertson FP , Bramley PM , Fraser PD . 2013 Functional characterization of long‐chain prenyl diphosphate synthases from tomato. Biochemical Journal 449: 729–740. 23126257 10.1042/BJ20120988

[nph16431-bib-0034] Kautsar SA , Suarez Duran HG , Blin K , Osbourn A , Medema MH . 2017 plantiSMASH: automated identification, annotation and expression analysis of plant biosynthetic gene clusters. Nucleic Acids Research 45: W55–W63. 28453650 10.1093/nar/gkx305 PMC5570173

[nph16431-bib-0035] Keeling CI , Weisshaar S , Ralph SG , Jancsik S , Hamberger B , Dullat HK , Bohlmann J . 2011 Transcriptome mining, functional characterization, and phylogeny of a large terpene synthase gene family in spruce (*Picea* spp.). BMC Plant Biology 11: 43. 21385377 10.1186/1471-2229-11-43 PMC3058080

[nph16431-bib-0036] Köksal M , Hu H , Coates RM , Peters RJ , Christianson DW . 2011 Structure and mechanism of the diterpene cyclase *ent*‐copalyl diphosphate synthase. Nature Chemical Biology 7: 431–433. 21602811 10.1038/nchembio.578 PMC3118866

[nph16431-bib-0037] Köksal M , Zimmer I , Schnitzler JP , Christianson DW . 2010 Structure of isoprene synthase illuminates the chemical mechanism of teragram atmospheric carbon emission. Journal of Molecular Biology 402: 363–373. 20624401 10.1016/j.jmb.2010.07.009 PMC2942996

[nph16431-bib-0038] Kumar S , Kempinski C , Zhuang X , Norris A , Mafu S , Zi J , Bell SA , Nybo SE , Kinison SE , Jiang Z *et al* 2016a Molecular diversity of terpene synthases in the liverwort *Marchantia polymorpha* . Plant Cell 28: 2632–2650. 27650333 10.1105/tpc.16.00062 PMC5134972

[nph16431-bib-0039] Kumar S , Stecher G , Tamura K . 2016b MEGA7: molecular evolutionary genetics analysis version 7.0 for bigger datasets. Molecular Biology and Evolution 33: 1870–1874. 27004904 10.1093/molbev/msw054 PMC8210823

[nph16431-bib-0040] Li G , Kollner TG , Yin Y , Jiang Y , Chen H , Xu Y , Gershenzon J , Pichersky E , Chen F . 2012 Nonseed plant *Selaginella moellendorffii* has both seed plant and microbial types of terpene synthases. Proceedings of the National Academy of Sciences, USA 109: 14711–14715. 10.1073/pnas.1204300109 PMC3437839 22908266

[nph16431-bib-0041] Martin DM , Aubourg S , Schouwey MB , Daviet L , Schalk M , Toub O , Lund ST , Bohlmann J . 2010 Functional annotation, genome organization and phylogeny of the grapevine (*Vitis vinifera*) terpene synthase gene family based on genome assembly, FLcDNA cloning, and enzyme assays. BMC Plant Biology 10: 226. 20964856 10.1186/1471-2229-10-226 PMC3017849

[nph16431-bib-0042] Matsuba Y , Nguyen TT , Wiegert K , Falara V , Gonzales‐Vigil E , Leong B , Schäfer P , Kudrna D , Wing RA , Bolger AM *et al* 2013 Evolution of a complex locus for terpene biosynthesis in *Solanum* . Plant Cell 25: 2022–2036. 23757397 10.1105/tpc.113.111013 PMC3723610

[nph16431-bib-0043] Matsuba Y , Zi J , Jones AD , Peters RJ , Pichersky E . 2015 Biosynthesis of the diterpenoid lycosantalonol via nerylneryl diphosphate in *Solanum lycopersicum* . PLoS ONE 10: e0119302. 25786135 10.1371/journal.pone.0119302 PMC4364678

[nph16431-bib-0044] McDowell ET , Kapteyn J , Schmidt A , Li C , Kang JH , Descour A , Shi F , Larson M , Schilmiller A , An LL *et al* 2011 Comparative functional genomic analysis of *Solanum* glandular trichome types. Plant Physiology 155: 524–539. 21098679 10.1104/pp.110.167114 PMC3075747

[nph16431-bib-0045] McGarvey DJ , Croteau R . 1995 Terpenoid metabolism. Plant Cell 7: 1015–1026. 7640522 10.1105/tpc.7.7.1015 PMC160903

[nph16431-bib-0046] Orlova I , Nagegowda DA , Kish CM , Gutensohn M , Maeda H , Varbanova M , Fridman E , Yamaguchi S , Hanada A , Kamiya Y *et al* 2009 The small subunit of snapdragon geranyl diphosphate synthase modifies the chain length specificity of tobacco geranylgeranyl diphosphate synthase in planta. Plant Cell 21: 4002–4017. 20028839 10.1105/tpc.109.071282 PMC2814502

[nph16431-bib-0047] Pichersky E , Noel JP , Dudareva N . 2006 Biosynthesis of plant volatiles: nature's diversity and ingenuity. Science 311: 808–811. 16469917 10.1126/science.1118510 PMC2861909

[nph16431-bib-0048] Pichersky E , Raguso RA . 2018 Why do plants produce so many terpenoid compounds? New Phytologist 220: 692–702. 27604856 10.1111/nph.14178

[nph16431-bib-0049] Rebers M , Kaneta T , Kawaide H , Yamaguchi S , Yang YY , Imai R , Sekimoto H , Kamiya Y . 1999 Regulation of gibberellin biosynthesis genes during flower and early fruit development of tomato. The Plant Journal 17: 241–250. 10097383 10.1046/j.1365-313x.1999.00366.x

[nph16431-bib-0050] Sainsbury F , Thuenemann EC , Lomonossoff GP . 2009 pEAQ: versatile expression vectors for easy and quick transient expression of heterologous proteins in plants. Plant Biotechnology Journal 7: 682–693. 19627561 10.1111/j.1467-7652.2009.00434.x

[nph16431-bib-0051] Sallaud C , Giacalone C , Töpfer R , Goepfert S , Bakaher N , Rösti S , Tissier A . 2012 Characterization of two genes for the biosynthesis of the labdane diterpene *Z*‐abienol in tobacco (*Nicotiana tabacum*) glandular trichomes. The Plant Journal 72: 1–17. 22672125 10.1111/j.1365-313X.2012.05068.x

[nph16431-bib-0052] Sallaud C , Rontein D , Onillon S , Jabès F , Duffe P , Giacalone C , Thoraval S , Escoffier C , Herbette G , Leonhardt N *et al* 2009 A novel pathway for sesquiterpene biosynthesis from *Z*, Z‐farnesyl pyrophosphate in the wild tomato *Solanum habrochaite*s. Plant Cell 21: 301–317. 19155349 10.1105/tpc.107.057885 PMC2648096

[nph16431-bib-0053] Sasaki K , Ohara K , Yazaki K . 2005 Gene expression and characterization of isoprene synthase from *Populus alba* . FEBS Letters 579: 2514–2518. 15848197 10.1016/j.febslet.2005.03.066

[nph16431-bib-0054] Schilmiller AL , Miner DP , Larson M , McDowell E , Gang DR , Wilkerson C , Last RL . 2010 Studies of a biochemical factory: tomato trichome deep expressed sequence tag sequencing and proteomics. Plant Physiology 153: 1212–1223. 20431087 10.1104/pp.110.157214 PMC2899918

[nph16431-bib-0055] Schilmiller AL , Schauvinhold I , Larson M , Xu R , Charbonneau AL , Schmidt A , Wilkerson C , Last RL , Pichersky E . 2009 Monoterpenes in the glandular trichomes of tomato are synthesized from a neryl diphosphate precursor rather than geranyl diphosphate. Proceedings of the National Academy of Sciences, USA 106: 10865–10870. 10.1073/pnas.0904113106 PMC2705607 19487664

[nph16431-bib-0056] Shao J , Chen QW , Lv HJ , He J , Liu ZF , Lu YN , Liu HL , Wang GD , Wang Y . 2017 (+)‐Thalianatriene and (−)‐retigeranin B catalyzed by sesterterpene synthases from *Arabidopsis thaliana* . Organic Letters 19: 1816–1819. 28350168 10.1021/acs.orglett.7b00586

[nph16431-bib-0057] Sharkey TD , Gray DW , Pell HK , Breneman SR , Topper L . 2013 Isoprene synthase genes form a monophyletic clade of acyclic terpene synthases in the TPS‐b terpene synthase family. Evolution 67: 1026–1040. 23550753 10.1111/evo.12013

[nph16431-bib-0058] Sommer S , Severin K , Camara B , Heide L . 1995 Intracellular‐localization of geranylpyrophosphate synthase from cell‐cultures of *Lithospermum Erythrorhizon* . Phytochemistry 38: 623–627.

[nph16431-bib-0059] Sun P , Schuurink RC , Caissard JC , Hugueney P , Baudino S . 2016 My way: noncanonical biosynthesis pathways for plant volatiles. Trends in Plant Science 21: 884–894. 27475252 10.1016/j.tplants.2016.07.007

[nph16431-bib-0060] Tholl D , Kish CM , Orlova I , Sherman D , Gershenzon J , Pichersky E , Dudareva N . 2004 Formation of monoterpenes in *Antirrhinum majus* and *Clarkia breweri* flowers involves heterodimeric geranyl diphosphate synthases. Plant Cell 16: 977–992. 15031409 10.1105/tpc.020156 PMC412871

[nph16431-bib-0061] Tholl D , Lee S . 2011 Terpene specialized metabolism in *Arabidopsis thaliana* . The Arabidopsis Book 9: e0143. 22303268 10.1199/tab.0143 PMC3268506

[nph16431-bib-0062] Thompson JD , Higgins DG , Gibson TJ . 1994 CLUSTAL W: improving the sensitivity of progressive multiple sequence alignment through sequence weighting, position‐specific gap penalties and weight matrix choice. Nucleic Acids Research 22: 4673–4680. 7984417 10.1093/nar/22.22.4673 PMC308517

[nph16431-bib-0063] Tomato Genome Consortium . 2012 The tomato genome sequence provides insights into fleshy fruit evolution. Nature 485: 635–641. 22660326 10.1038/nature11119 PMC3378239

[nph16431-bib-0064] van Schie CC , Haring MA , Schuurink RC . 2007 Tomato linalool synthase is induced in trichomes by jasmonic acid. Plant Molecular Biology 64: 251–263. 17440821 10.1007/s11103-007-9149-8 PMC1876254

[nph16431-bib-0065] Vaughan MM , Wang Q , Webster FX , Kiemle D , Hong YJ , Tantillo DJ , Coates RM , Wray AT , Askew W , O'Donnell C *et al* 2013 Formation of the unusual semivolatile diterpene rhizathalene by the Arabidopsis class I terpene synthase TPS08 in the root stele is involved in defense against belowground herbivory. Plant Cell 25: 1108–1125. 23512856 10.1105/tpc.112.100057 PMC3634680

[nph16431-bib-0066] Vranová E , Coman D , Gruissem W . 2013 Network analysis of the MVA and MEP pathways for isoprenoid synthesis. Annual Review of Plant Biology 64: 665–700. 10.1146/annurev-arplant-050312-120116 23451776

[nph16431-bib-0067] Wang CY , Chen QW , Fan DJ , Li JX , Wang GD , Zhang P . 2016 Structural analyses of short‐chain prenyltransferases identify an evolutionarily conserved GFPPS clade in Brassicaceae plants. Molecular Plant 9: 195–204. 26537048 10.1016/j.molp.2015.10.010

[nph16431-bib-0068] Wang G , Dixon RA . 2009 Heterodimeric geranyl(geranyl)diphosphate synthase from hop (*Humulus lupulus*) and the evolution of monoterpene biosynthesis. Proceedings of the National Academy of Sciences, USA 106: 9914–9919. 10.1073/pnas.0904069106 PMC2701037 19482937

[nph16431-bib-0069] Wang Q , Huang XQ , Cao TJ , Zhuang Z , Wang R , Lu S . 2018 Heteromeric geranylgeranyl diphosphate synthase contributes to carotenoid biosynthesis in ripening fruits of red pepper (*Capsicum annuum* var. *conoides*). Journal of Agricultural and Food Chemistry 66: 11691–11700. 30339374 10.1021/acs.jafc.8b04052

[nph16431-bib-0070] Wang Q , Jia MR , Huh JH , Muchlinski A , Peters RJ , Tholl D . 2016 Identification of a dolabellane type diterpene synthase and other root‐expressed diterpene synthases in Arabidopsis. Frontiers in Plant Science 7: 1761. 27933080 10.3389/fpls.2016.01761 PMC5122590

[nph16431-bib-0071] Wei G , Jia Q , Chen X , Köllner TG , Bhattacharya D , Wong GK , Gershenzon J , Chen F . 2019 Terpene biosynthesis in red algae is catalyzed by microbial type but not typical plant terpene synthases. Plant Physiology 179: 382–390. 30538166 10.1104/pp.18.01413 PMC6426406

[nph16431-bib-0072] Yoo SD , Cho YH , Sheen J . 2007 Arabidopsis mesophyll protoplasts: a versatile cell system for transient gene expression analysis. Nature Protocol 2: 1565–1572. 10.1038/nprot.2007.199 17585298

[nph16431-bib-0073] Zerbe P , Bohlmann J . 2015 Plant diterpene synthases: exploring modularity and metabolic diversity for bioengineering. Trends in Biotechnology 33: 419–428. 26003209 10.1016/j.tibtech.2015.04.006

[nph16431-bib-0074] Zhou F , Wang CY , Gutensohn M , Jiang L , Zhang P , Zhang D , Dudareva N , Lu S . 2017 A recruiting protein of geranylgeranyl diphosphate synthase controls metabolic flux toward chlorophyll biosynthesis in rice. Proceedings of the National Academy of Sciences, USA 114: 6866–6871. 10.1073/pnas.1705689114 PMC5495272 28607067

[nph16431-bib-0075] Zhuang X , Köllner TG , Zhao N , Li G , Jiang Y , Zhu L , Ma J , Degenhardt J , Chen F . 2012 Dynamic evolution of herbivore‐induced sesquiterpene biosynthesis in sorghum and related grass crops. The Plant Journal 69: 70–80. 21880075 10.1111/j.1365-313X.2011.04771.x

