# Supplementary material for: The complete functional characterisation of the terpene synthase family in tomato
Source: New Phytol. 2020 Feb 19;226(5):1341–60. doi: 10.1111/nph.16431 (PMC7422722; doi:10.1111/nph.16431)
Supplement: Supplementary file 1 — Fig. S1 Annotated sequences of TPS51 and TPS52. Fig. S2 SDS‐PAGE analysis of the purified recombinant His‐tagged TPS proteins. Fig. S3 Mass spectra of identified terpenes from Figs 3–5 & 10. Fig. S4 GC–MS analysis of elemol and (+)‐hedycaryol. Fig. S5 GC–MS analysis of the products formed in planta by transiently co‐expressing TPS with CPT genes in Nicotiana benthamiana leaves. Fig. S6 Analysis of tomato terpene synthases with isoprene synthases from other plants. Fig. S7 Sequence alignment of the proteins encoded by the functional tomato TPS‐c genes and CPS genes from other plants. Fig. S8 Predictions of subcellular localisation for TPS and prenyltransferase proteins made by TargetP 1.1 and ChloroP 1.1. Fig. S9 Phylogenetic analysis of tomato TPT homologues and TPTs identified in other plants. Fig. S10 GC–MS analysis of the products formed in planta by transiently co‐expressing TPS with TPT genes in Nicotiana benthamiana leaves. Fig. S11 Sequence alignment of five Arabidopsis sesterterpene synthases and tomato TPSs from TPS‐a, TPS‐b and TPS‐g clades. Fig. S12 Sequence alignment of the proteins encoded by the functional tomato TPS‐a genes. Fig. S13 Sequence alignment of the proteins encoded by the functional tomato TPS‐b and TPS‐g genes. Fig. S14 Sequence alignment of the proteins encoded by the functional tomato TPS‐e/f genes. Fig. S15 Sequence analysis of TPT homologues from tomato and other plants. Fig. S16 GC–MS analysis of terpenes extracted from tomato leaves. Fig. S17 Correlation analysis of terpene biosynthesis genes and volatile terpenes from tomato. Fig. S18 Terpene biosynthesis gene clusters identified from tomato genome by plantiSMASH. Methods S1 Details on experimental procedures. Table S1 Primers used in this study. Table S2 A list of terpene compounds identified from Figs 3–5 & 10. Table S3 A list of tomato terpene synthase genes, their characteristics and the enzymes they encode. Table S4 A list of tomato terpene synthase pseudogenes. Table S5 A list [file NPH-226-1341-s001.pdf]

New Phytologist **Supporting Information Figs S1-S18, Methods S1 and Tables S1-S7**

Article title: The complete functional characterization of the terpene synthase family in tomato

Authors: Fei Zhou and Eran Pichersky

Article acceptance date: 5 January 2020

The following Supporting Information is available for this article:

Fig. S1 Annotated sequences of *TPS51* and *TPS52*.

Fig. S2 SDS-PAGE analysis of the purified recombinant His-tagged TPS proteins.

Fig. S3 Mass spectra of identified terpenes from Figs 3-5 and 10.

Fig. S4 GC-MS analysis of elemol and (+)-hedycaryol.

Fig. S5 GC-MS analysis of the products formed in planta by transiently co-expressing TPS with CPT genes in *Nicotiana benthamiana* leaves.

Fig. S6 Analysis of tomato terpene synthases with isoprene synthases from other plants.

Fig. S7 Sequence alignment of the proteins encoded by the functional tomato TPS-c genes and CPS genes from other plants.

Fig. S8 Predictions of subcellular localization for TPS and prenyltransferase proteins made by TargetP 1.1 (a) and ChloroP 1.1 (b).

Fig. S9 Phylogenetic analysis of tomato TPT homologs and TPTs identified in other plants.

Fig. S10 GC-MS analysis of the products formed in planta by transiently co-expressing TPS with TPT genes in *Nicotiana benthamiana* leaves.

Fig. S11 Sequence alignment of five Arabidopsis sesterterpene synthases and tomato TPSs from TPS-a, TPS-b and TPS-g clades.

Fig. S12 Sequence alignment of the proteins encoded by the functional tomato TPS-a genes.

Fig. S13 Sequence alignment of the proteins encoded by the functional tomato TPS-b and TPS-g genes.

Fig. S14 Sequence alignment of the proteins encoded by the functional tomato TPS-e/f

genes.

Fig. S15 Sequence analysis of TPT homologs from tomato and other plants.

Fig. S16 GC-MS analysis of terpenes extracted from tomato leaves.

Fig. S17 Correlation analysis of terpene biosynthesis genes and volatile terpenes from tomato.

Fig. S18 Terpene biosynthesis gene clusters identified from tomato genome by plantiSMASH.

Table S1 Primers used in this study.

Table S2 A list of terpene compounds identified from Figs 3-5 and 10.

Table S3 A list of tomato terpene synthase genes, their characteristics, and the enzymes they encode.

Table S4 A list of tomato terpene synthase pseudogenes.

Table S5 A list of the *trans*-prenyltransferase genes in tomato.

Table S6 A list of terpene compounds produced by all the functional tomato terpene synthases.

Table S7 A list of Arabidopsis terpene synthase genes, their characteristics, and the enzymes they encode.

Methods S1 Details on experimental procedures.

Fig. S1 Annotated sequences of *TPS51* and *TPS52*. Exons are shading in yellow, gaps in the reference genome (https://solgenomics.net/organism/Solanum_lycopersicum/genome; the 2017 release of version SL3.0) are underlined and filled by sequencing the cloned genomic DNA. Stop codons are shown in red. The obtained genomic sequences of *TPS51* and *TPS52* have been deposited to GenBank under accession numbers MN366354 and MN366355, respectively. The genomic sequences of other genes studied in this work can be found in the tomato genome database under loci listed in Tables S3-S5.

```
>TPS51 genomic sequence
AATTATTTATTTTTATGAAAAGTATTTTCTTAACGAATCTTTTCAGAGTTGAATTTTGACCATTATAAGTT
GGTCAGTTGAGCTCGTTGAATATCCAACTAACATCTATTTAATCAAAATGTTGGTTCTTTTCTTTCACCTA
AATGAACAAGAAAAATGGATGGATTTGGTGATGAGGCAGAGATTATACGTCGGTGTGCCAATCATCATCCT
TCTGTTTGGGGAGACCATTTTCTTGCTTATGCTAATCTCTTGGTAAATTTGGATTTAATTAATTACTTTAT
GTTATATATATTCTTTATGTATTATTATACGTATATACATACCATAAATTTCGAAATTTACTCTTGCACGT
AGGGAGCCAATGAATGTGAAAAGAAGAAACATGAAGACCTAAAAAAAGAAGCGAGGAAAATGCTAGTGCTT
TCTCCTTCCAAATCTTTGCAAAAACTTGAACTTATAAACACAATCCAATTGCTTGGAGTGTCATACCATTT
TGAACATGAGATTGAGGAATCATTGAGTGAAATCTATAATGGTTATGAAGAATGGATTGGTGAAAGTCATG
ACCTTCATGATGTTGCTCTAAGCTTTCGATTACTCAGGCAACAAGGTTATTACGTCTCATCAGGTGAGTAC
CCCCAACACCAACTAGAGATTATGTGATGAGTTTAAATTGTTGGTAAAAGTTTTTTTAACTTTCCTATACT
AACGGTACAAGTATGGTTATTTTTTAAGCAGATGTTTTTAGGAAGTTCACTGATGAGAGAGGAAATTACAA
GGAAACTTTGGCCAGCGACGTACAAGGATTATTGAGCTTGTATGAGGCAGCGCAACTCAGAGTAGATGATG
AAGAAATTCTGGATGAAGCAATTAATTTCACCACTACTCACTTGAAGCTACTTTTGCCTAATTTGAGTGAT
CCCCTTGCGACTCAAGTTAGTAACGCGCTGAAATTTCCAATCAACAATATTATAGTGAGGGTAGCAACTAG
AAAATACATTTCATTTTACCAAGAAGATAAATCACACAATGAAATGTTACTACATTTTGCTAAATTGGACT
TCAACATTTTACAAAGGCTGCACAAAAGGGAGCTATGTGATATCACAAGGTAATTGACAAGTTAAACATTT
ACTAATCGATTTAACTTATATAATTCCTGAAATTATAATTTTGTCTCTACAAATTTTAGGTGGTGGAAAGA
CTCGGAAATCGCGCAGGCACTACCTTTTGCTAGAGATAGAGTGGTTGAATTATATTTTTGGAGTTTGGGTG
TCTATTTTGAGCCTCACTATAGTGTTGCAAGGAAAATACTAACCAAAGTGTTATGTTTCTGTTCTATTATG
GATGATACTTACGATACCTATGGCACACTGGATGAACTTACTCTCCTCACAACGGCCATTGAAAGGTTTAA
GTTCTTTTCTCACATCAAGATTTTTATGTTTCTAACACTAAAAATGAATTATCACCGTAAAATATAAGAAA
TTTTGAGAATGTGATATATGTATCGTAGTTGGGACATTGATGCTTCGGAACAATTACCGCCCTATATGAAG
CTCTCCTACCGTGCTCTTGTACAAGTTTACACTGAAGCAGAGAGAGAATTGGAAAATCTTGGAAATAAGAT
GACATTTAGAGTCAAATATTCCATCAATGAGGTAATCAAAGAATTATTACTGAATGATGAAAATATAGTCA
AAATTAAAAAAGCTTGTTACTTTTTTCAGATGAAAAAGTTGTTGAGGGCTTACTTCCAAGAGGCCAAATGG
TATCATGAGAAAGTTGTGCCAACAATGGAACAATATATAAAGAATGGAATTCCATCCAGCACTTACCTATT
GCTTGCTACTACTTCTTGGTTAGGCATGGGAGATGTAGCAACTAAGGATGCGTTTGATTGGATATCGAATG
AACCTACAATTCTTGTAGCTTTGTCTATCATTGCAAGATTACTCAATGATCTCGTAACACATGAGGTATTA
TATTGTACGTGTAAATAATAACTCAAGGATAAATAGGAAGTGCTACATATACAAATTGAATATGAGGTACT
ATTTTTGCTAGTCTTTAAACTTGTTTGTCTGATTATATTATGGATACAGATAGAAGTGGAAAGAGGAGATG
TAGCATCGGGTATTGAATGTTACATGAACGAGTATGGTGCCACAAAAGAAGAAGCTTACATGGAGACAAGG
AAAATAATAGAGAATAATTGGAAGGTTTTGAATAGAGGATGCCTAAAGCCTACAACAGTACCAAGAGTTTT
```

ACTCATGCCAGTGCTCAACCTTACGAGGGTGGCAGAGTTCTTTTATAAAGATGATGATGCTTATACTTTTT
CCACAAATAACTTGAAAGACATCATCTCTTTGGTGCTAATTGATCCTATTACAACATAAGAGAACTTTACA
ATGAAACTACTAGTACTTCATAAACATCTAACATTTTTTGATGTTGCTTATTTCTGTGCTAAGTTCTAGCT
TAGCTAAAATTGCCTATGTTTTATTTATGTACTTTGTTATGGTTGTCAATTGATATATGGACTGCTTTGAT
ACAGTTAATACAAACTACAATATATATAAAATCACCTAGCATTATATATAGATTAGTCTTTCGAC

>TPS52 genomic sequence
GAGCTCAAACTAACACCCATTTGTTCAATACTGTTTTTTCTCTTCACTATATATATAGTAACTTTAAAGAA
GAAGAAAAATGGTCGTTGGATTTGGTGGTGAAGCGGAGATTACACGTCGATGTGCCAATCATCATCCTTCT
GTTTGGGGAGACCATTTTCTTACCTATGCTAATCTCTTGGTAATTAAGTGCTTGATTTATGTTGTGAATAT
TATTCATGATTTTTAACAAATCATATTATAAACAAATTATGAAATTTATTTGTGTAGGGAGCCAATGAATG
GGAAGAGAAGGAACATGAAGACCAAAAAGGAGGAGTGAGAAAAATGCTAGTGCTTTCTCCTTCCAAATCTT
TGCAAAAACTTGAACTTATAAACACAATCCAATTGCTTGGAGTGTCATACCATTTTGAACATGAGATTGAG
GAATCATTGAGTGAAATCTATAATGGTTATGAAGAATGGATTGGTGAAAGTCATGACCTTCATGATGTTGC
TCTAAGCTTTCGATTACTCAGGCAACAAGGTTATTACGTCTCATCAGGTGAGTACCCCCAACACCAACTAG
AGATTATGTGATGAGTTTAAATTGTTGGTAAAAGTTTTTTTAACTTTCCTATACTAACGGTACAAGTATGG
TTATTTTTTTAAGCAGATGTTTTTAGGAAGTTCACTGATGAGAGAGGAAATTACAAGGAAACTTTGGCCAGC
GACGTACAAGGATTATTGAGCTTGTATGAGGCAGCGCAACTCAGAGTAGATGATGAAGAAATTCTGGATGA
AGCAATTAATTTCACCACTACTCACTTGAAGCTACTTTTGCCTAATTTGAGTGATCCCCTTGCGACTCAAG
TTAGTAACGCGCTGAAATTTCCAATCAACAATATTATAGTGAGGGTAGCAACTAGAAAATACATTTCATTT
TACCAAGAAGATAAATCACACAATGAAATGTTACTACATTTTGCTAAATTGGACTTCAACATTTTACAAAG
GCTGCACAAAAGGGAGCTATGTGATATCACAAGGTAATTGACAAGTTAAACATTTACTAATCGATTTAACT
TATATAATTCCTGAAATTATAATTTTGTCTCTACAAATTTTAGGTGGTGGAAAGACTCGGAAATCGCGCAG
GCACTACCTTTTGCTAGAGATAGAGTGGTTGAATTATATTTTTGGAGTTTGGGTGTCTATTTTGAGCCTCA
CTATAGTGTTGCAAGGAAAATACTAACCAAAGTGTTATGTTTCTGTTCTATTATGGATGATACTTACGATA
CCTATGGCACACTGGATGAACTTACTCTCCTCACAACGGCCATTGAAAGGTTTAAGTTCTTTTCTCACATC
AAGATTTTTATGTTTCTAACACTAAAAATGAATTATCACCGTAAAATATAAGAAATTTTGAGAATGTGATA
TATGTATCGTAGGTGGGACATTGATGCTTCGGAACAATTACCTTCATATATGAAGCTCTCCTACCGTGCTC
TTGTACAAGTTTACAATGAAACGGAGAAAGAATTGGAAAATCTTGGAAATAAGATGACATATAGAGTCAAA
TATTCCATCAATGAGGTAATCAAGAATTATTACTGAATGATGAAAATATAGTCAAGATTAAAAGCTTGTTA
CTTTTTTTCAGATGAAAAAGTTGTTGAGGGCTTACTTCCAAGAGGCCAAATGGTATCATGGGAAGGATGTG
CCAACAATGGAACAATATATAAAGAATGGAATTCCATCAAGTACTTATCTATTGCTTGCCACTACTTCTTG
GTTAGGCATGGGAGACGTAGCAACTAAGGATGCGTTTGATTGGATATCGAATGAACCTACAATTCTTGTAG
CTTCGTCTATCATTGCAAGATTACTCAATGATCTCGTAACACATGAGGTATTATATTGTACGCGCAAATAT
TAATTATGAGGGTATAATTTCTTTATATTTTCACTTGTTTGCCTAATTATATTATGGATACAGATAGAAGT
GGAAAGAGGAGATGTAGCATCGGGTATTGAATGTTACATGAACGAGTATGGTGCCACAAAAGAAGAAGCTT
ACATGGAGATAAGGAAAATAATAGAGAATAATTGGAAGGATTTGAATAGAGGATGCCTAAAGCCTACAACA
GTACCAAGAGTTTTACTCATGCCAGTGCTCAACCTTACGAGGGTGGCAGAGTTTTTTTATAAAGACGAAGA
TGCTTATACTTTTTCGAAAAATAACTTGAAAGACGTCATCTCTATGGTGCTAATTGATCCTATAAAAGCAT
GAGAGAATTTCACTATGAAGGGAAAATAATAAATAAAAGTAGTAGTGCATGAACAACATCTGATGTTGCTT
CTTTATGGAGGTTGTGGCTTGTTTTTACTATTTTGTATCTTGATGTTGTTTTATCTTTGTGTCAGCTGTTG

Fig. S2 SDS-PAGE analysis of the purified recombinant His-tagged TPS proteins. T, total protein after IPTG induction; S, soluble fraction; P, purified fraction with Ni-NTA agarose as described in Methods S1.

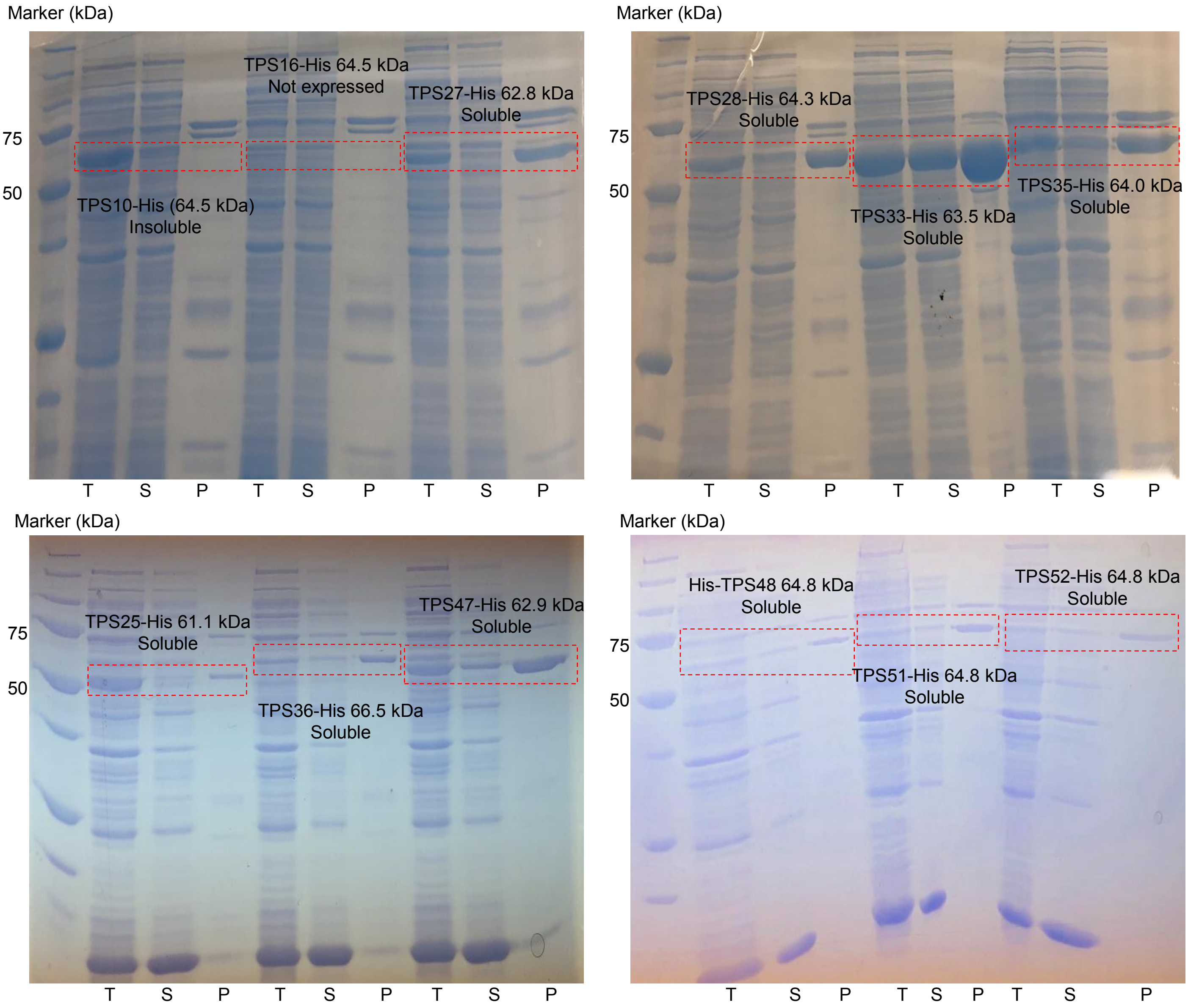

Fig. S3 Mass spectra of identified terpenes from Figs 3-5 and 10. The mass spectra of identified terpenes from NIST library are shown on the right. Compounds listed in order of elution from the Rxi-5Sil MS column, the enzymatic products are shown in bold. Detailed information can be found in Table S2.

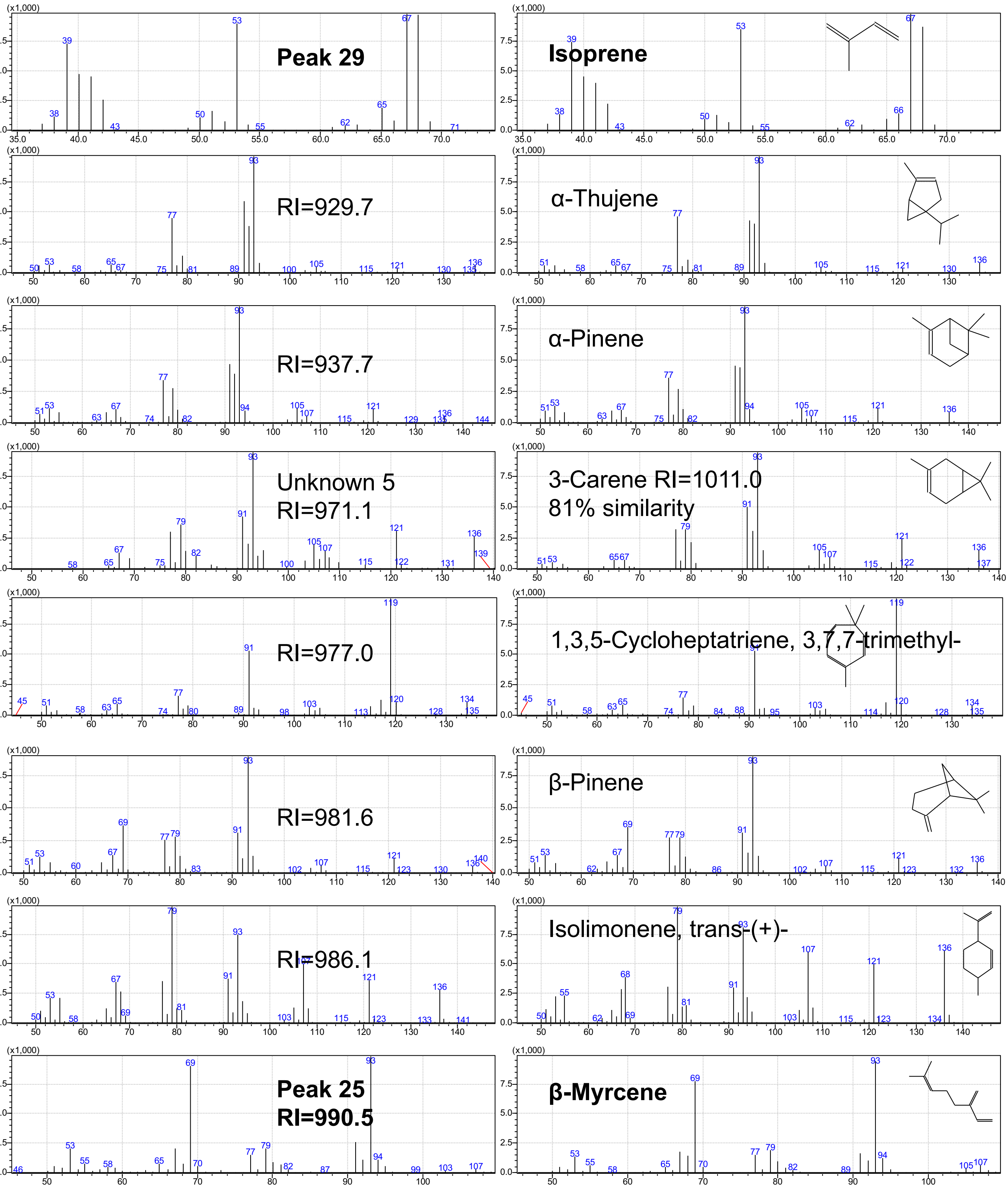

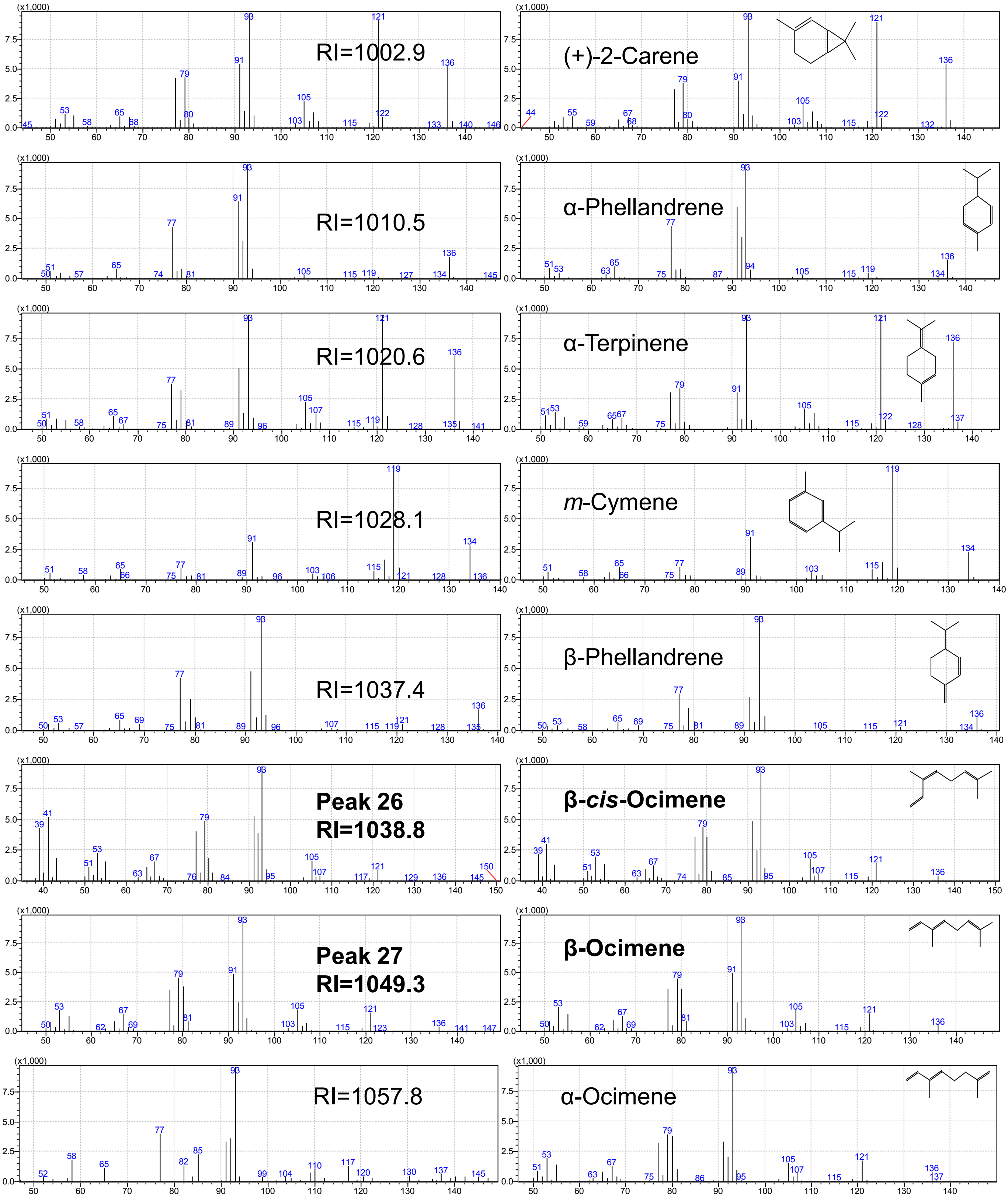

RI=1002.9
(+)-2-Carene
RI=1010.5
α-Phellandrene
RI=1020.6
α-Terpinene
RI=1028.1
m-Cymene
RI=1037.4
β-Phellandrene
Peak 26
RI=1038.8
β-cis-Ocimene
Peak 27
RI=1049.3
β-Ocimene
RI=1057.8
α-Ocimene

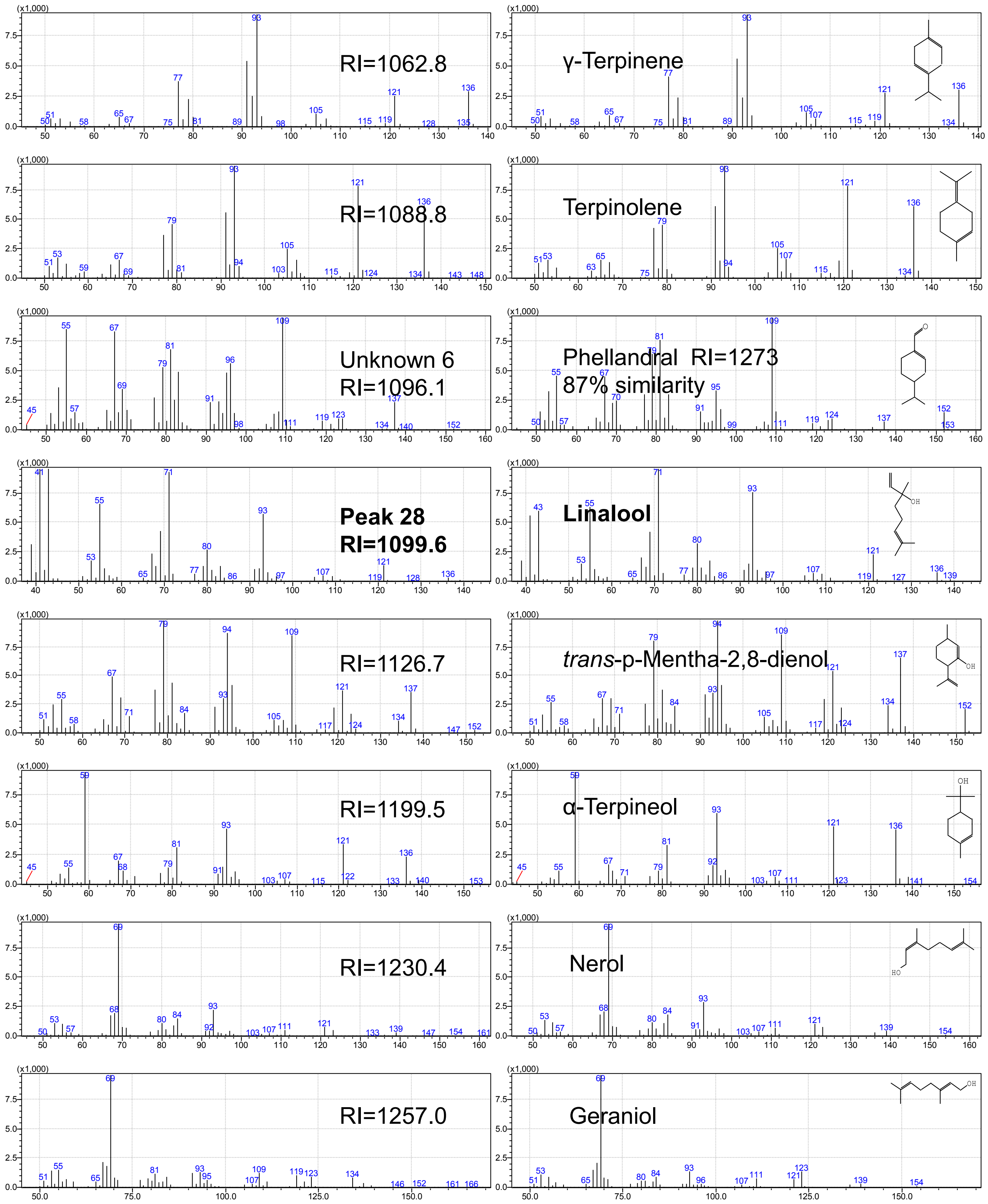
RI=1062.8
γ-Terpinene
RI=1088.8
Terpinolene
Unknown 6
RI=1096.1
Phellandral RI=1273
87% similarity
Peak 28
RI=1099.6
Linalool
RI=1126.7
trans-p-Mentha-2,8-dienol
RI=1199.5
α-Terpineol
RI=1230.4
Nerol
RI=1257.0
Geraniol

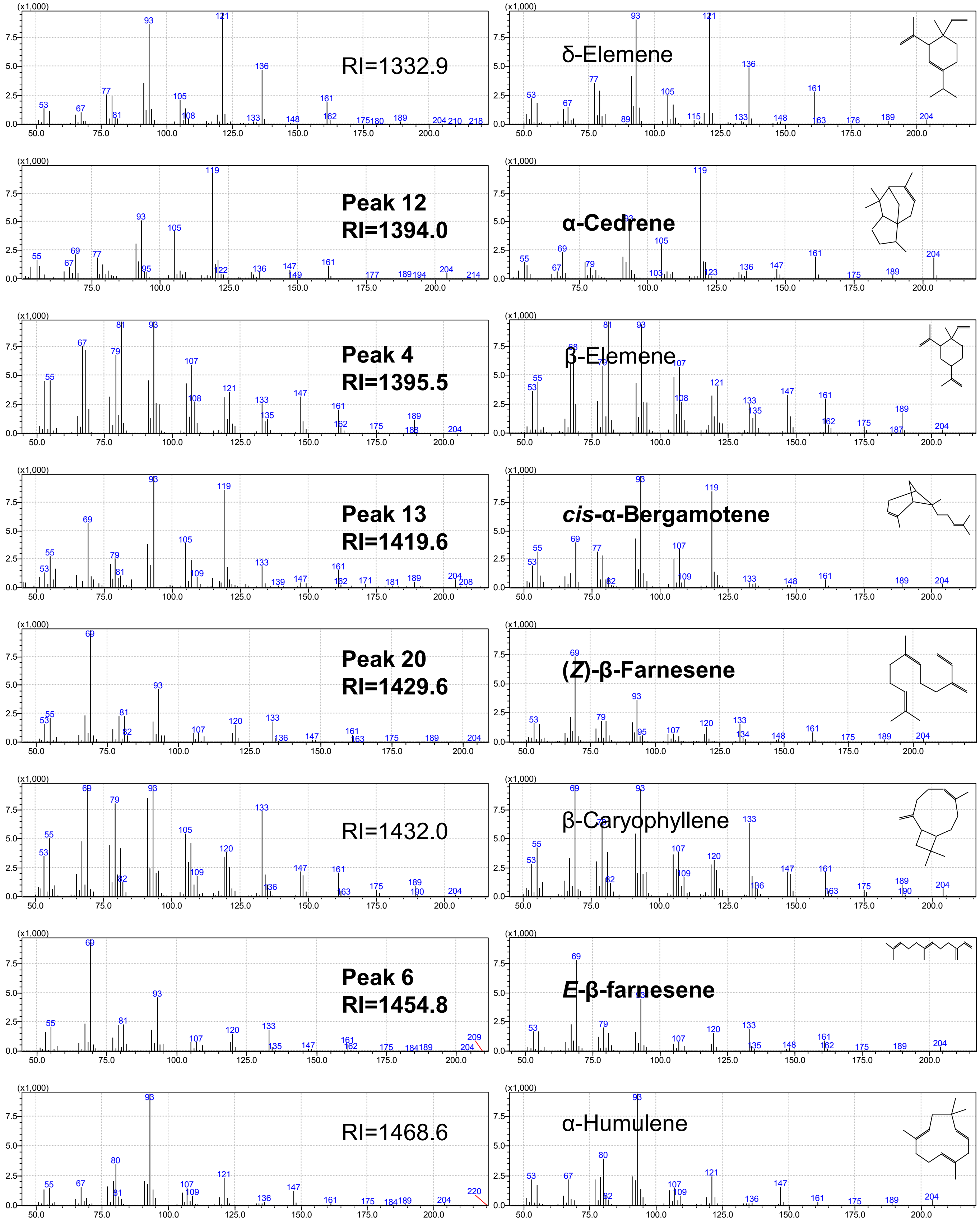
RI=1332.9
δ-Elemene
Peak 12
RI=1394.0
α-Cedrene
Peak 4
RI=1395.5
β-Elemene
Peak 13
RI=1419.6
cis-α-Bergamotene
Peak 20
RI=1429.6
(Z)-β-Farnesene
RI=1432.0
β-Caryophyllene
Peak 6
RI=1454.8
E-β-farnesene
RI=1468.6
α-Humulene

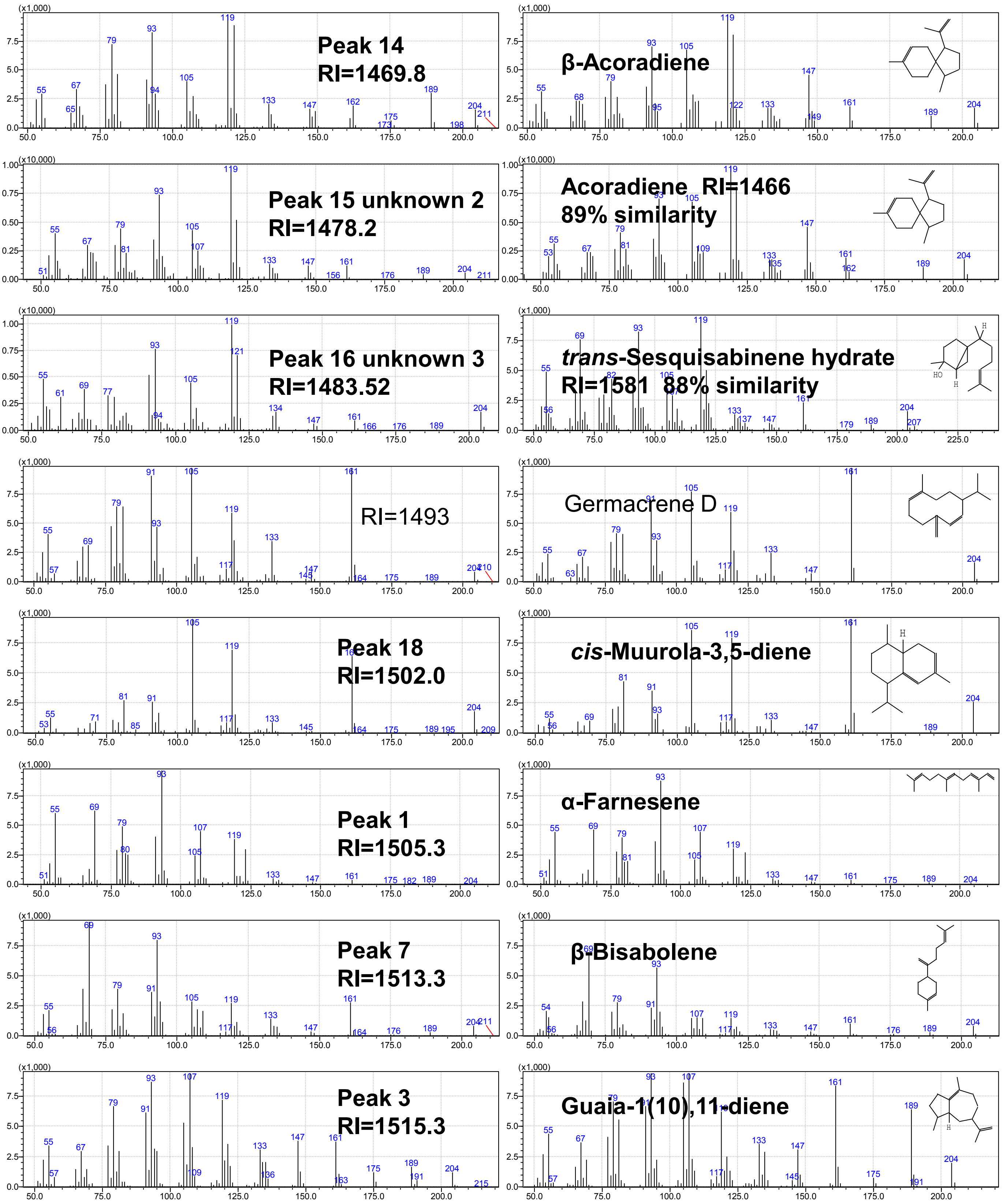

Peak 14
RI=1469.8
β-Acoradiene
Peak 15 unknown 2
RI=1478.2
Acoradiene RI=1466
89% similarity
Peak 16 unknown 3
RI=1483.52
trans-Sesquisabinene hydrate
RI=1581 88% similarity
RI=1493
Germacrene D
Peak 18
RI=1502.0
cis-Muurola-3,5-diene
Peak 1
RI=1505.3
α-Farnesene
Peak 7
RI=1513.3
β-Bisabolene
Peak 3
RI=1515.3
Guaia-1(10),11-diene

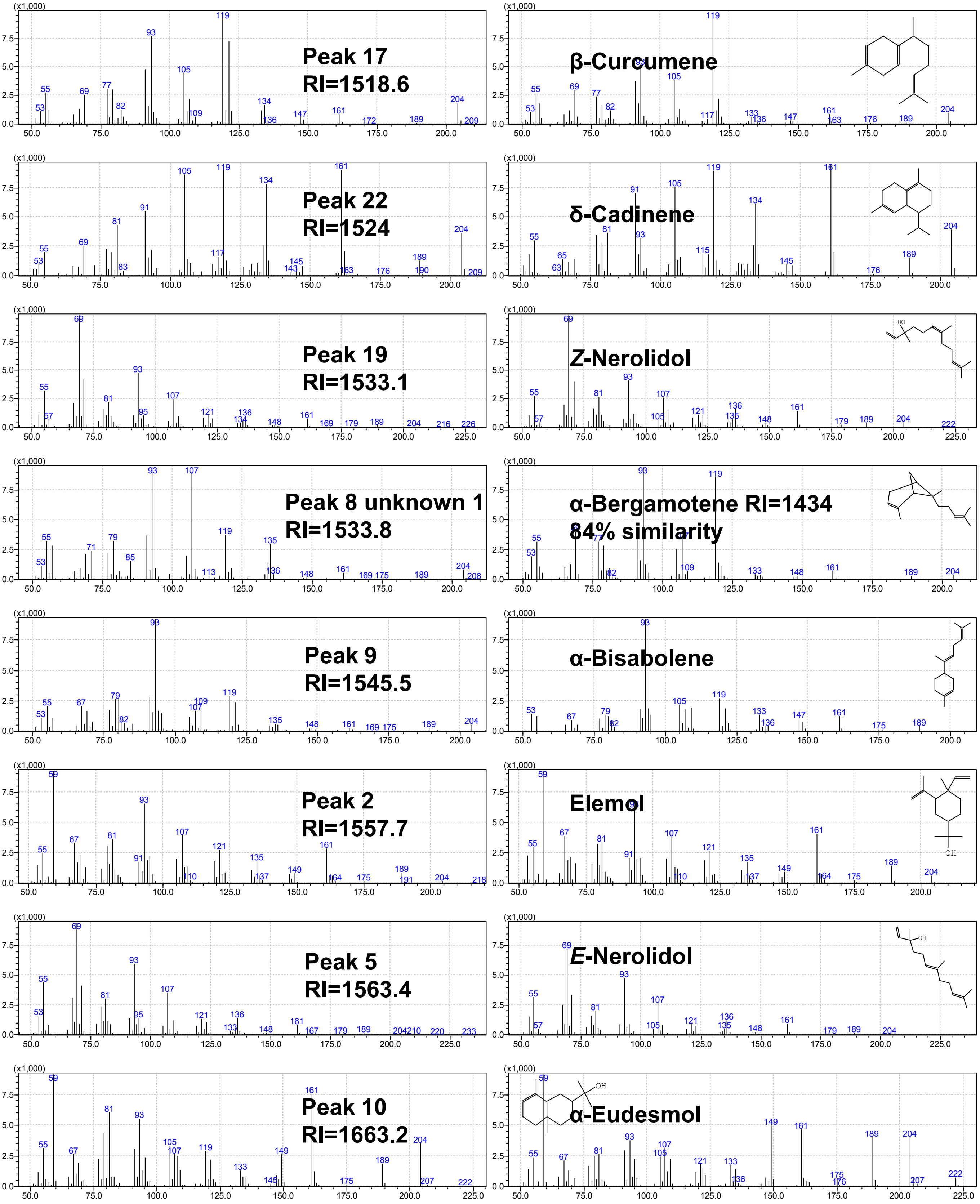

Peak 17
RI=1518.6
β-Curcumene
Peak 22
RI=1524
δ-Cadinene
Peak 19
RI=1533.1
Z-Nerolidol
Peak 8 unknown 1
RI=1533.8
α-Bergamotene RI=1434
84% similarity
Peak 9
RI=1545.5
α-Bisabolene
Peak 2
RI=1557.7
Elemol
Peak 5
RI=1563.4
E-Nerolidol
Peak 10
RI=1663.2
α-Eudesmol

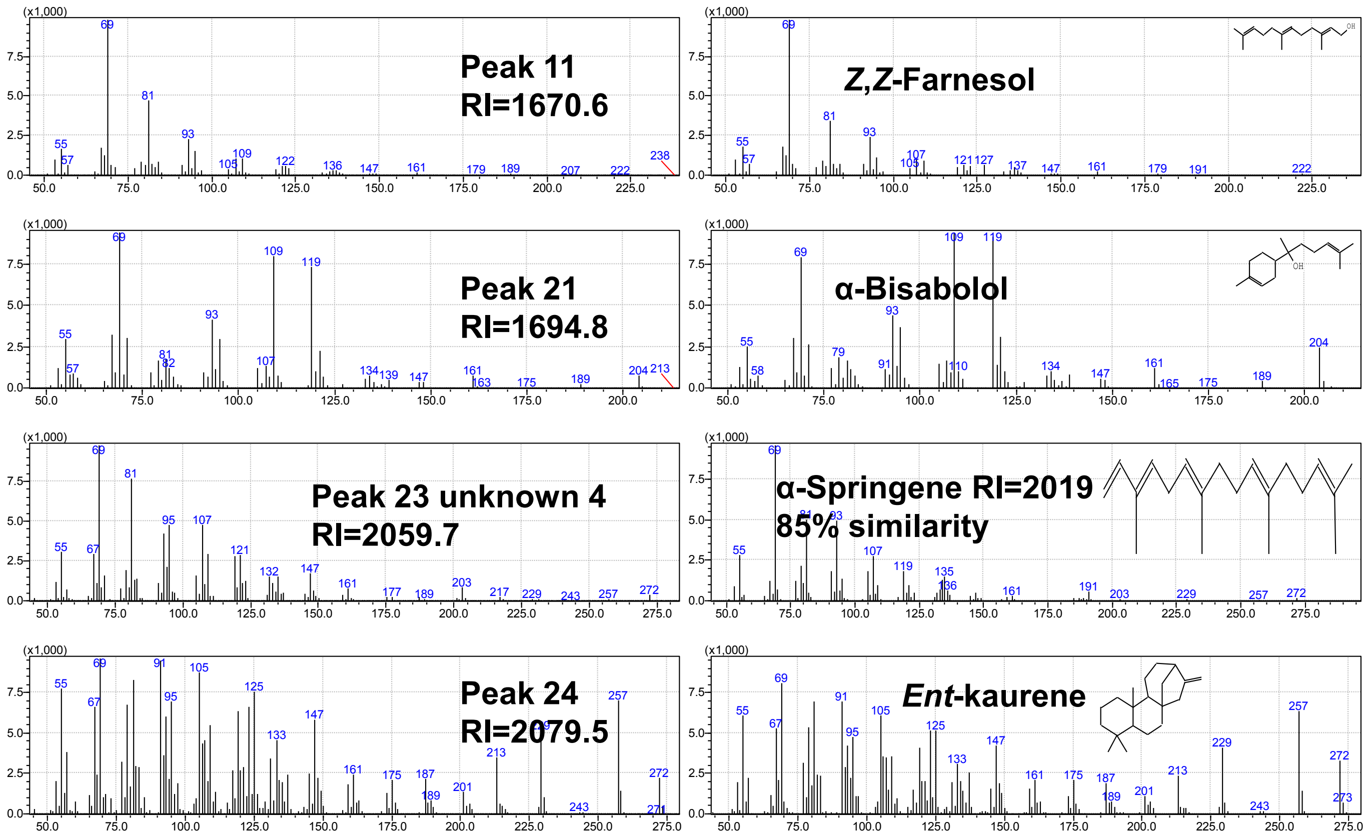

Peak 11
RI=1670.6
Z,Z-Farnesol
Peak 21
RI=1694.8
α-Bisabolol
Peak 23 unknown 4
RI=2059.7
α-Springene RI=2019
85% similarity
Peak 24
RI=2079.5
Ent-kaurene

Fig. S4 GC-MS analysis of elemol and (+)-hedycaryol. Elemol is the thermal breakdown product of (+)-hedycaryol, which can be detected by lowing the injection port temperature.

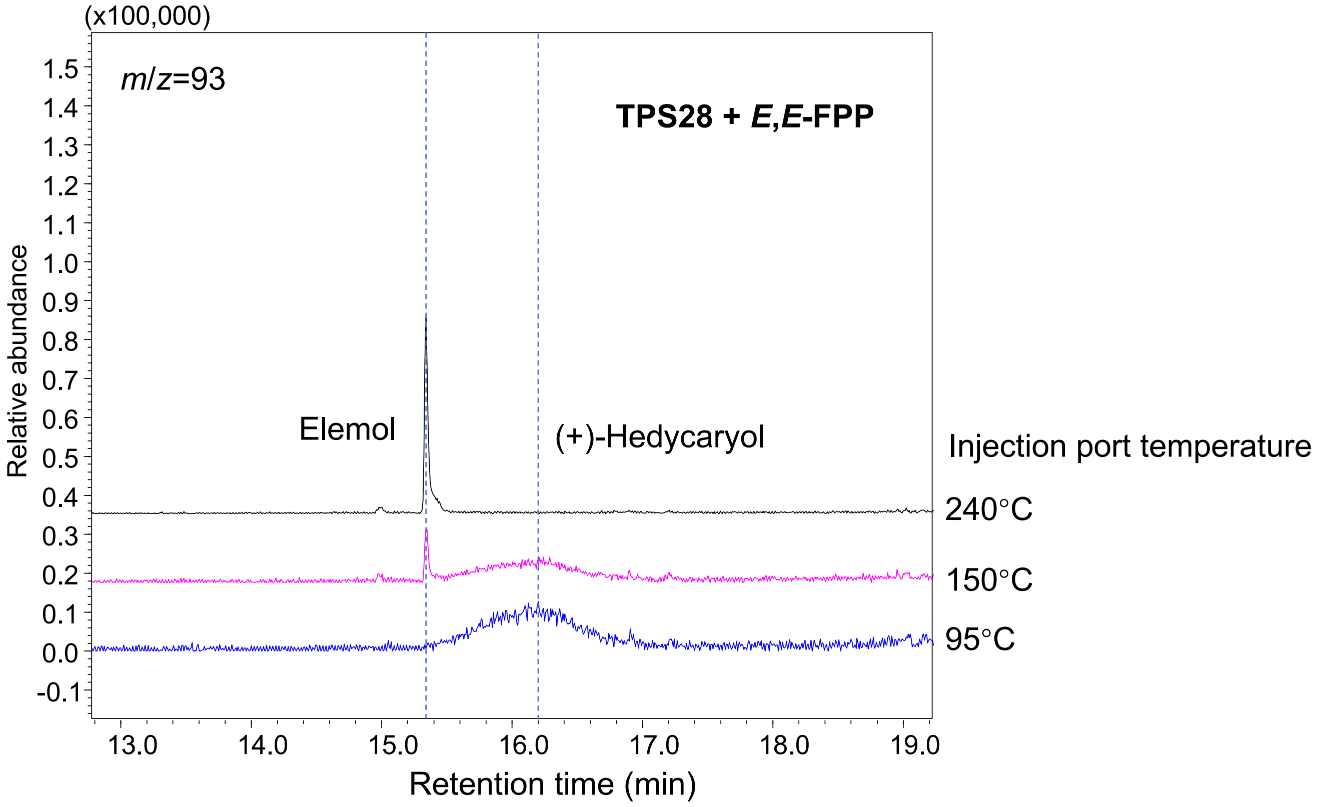

Fig. S5 GC-MS analysis of the products formed in planta by transiently co-expressing TPS with CPT genes in *Nicotiana benthamiana* leaves.

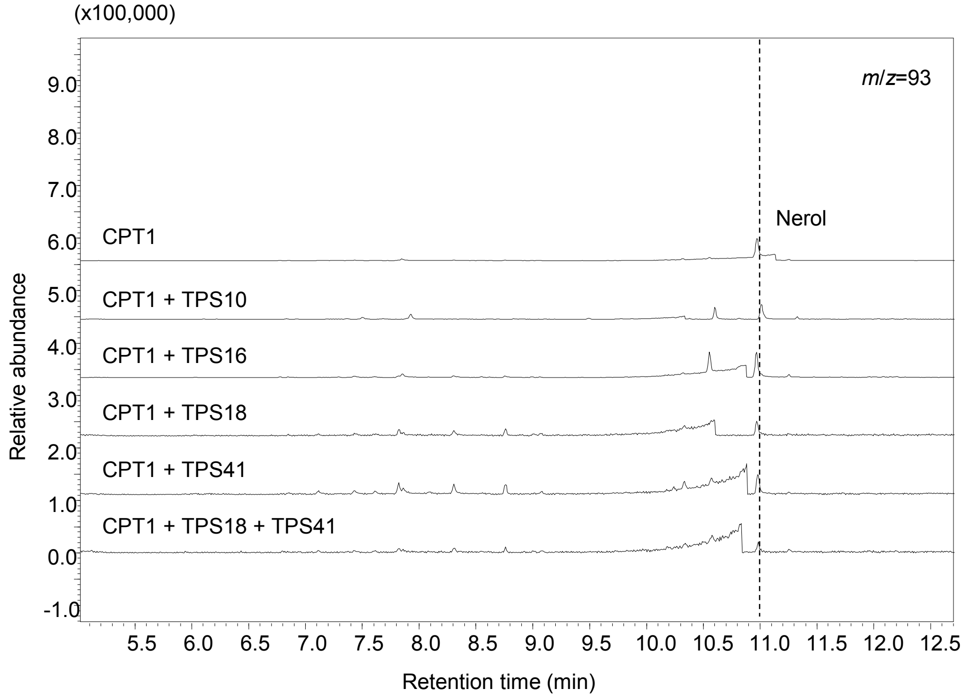

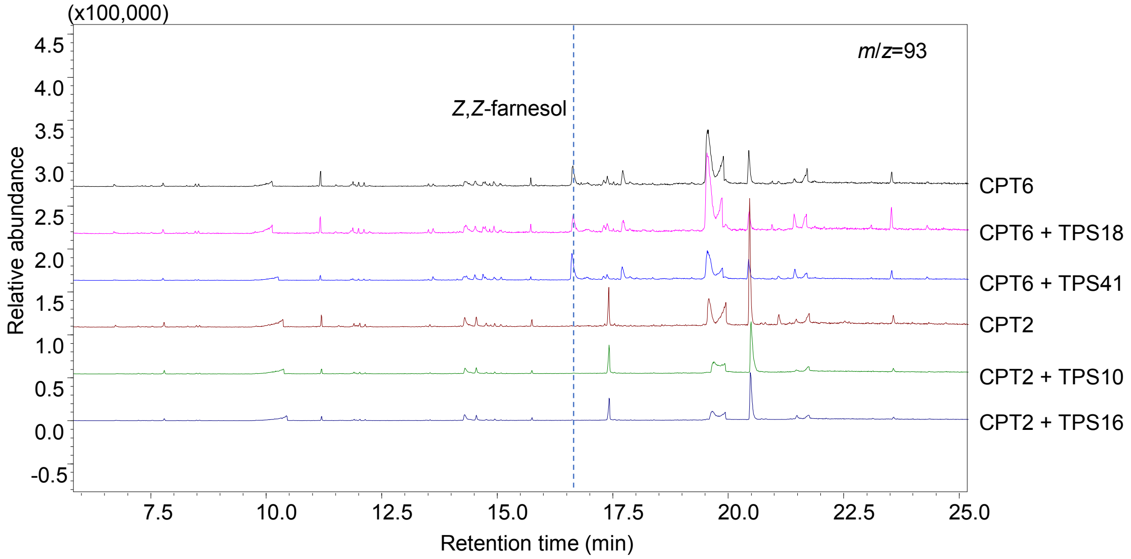

Fig. S6 Analysis of tomato terpene synthases with isoprene synthases from other plants. (a) Phylogenetic analysis of 34 tomato TPSs with three functionally characterized isoprene synthases. The three TPSs clustered with known isoprene synthase are shown in bold and shaded in green. (b) Multiple sequence alignment tomato TPSs from TPS-a, TPS-b and TPS-g clades with isoprene synthases. Black shading indicates identical amino acid residues and grey shading are the conserved or semi-conserved substitutions observed and dashes indicate gaps. Amino acids belonging to the 'isoprene score' amino acids are shown in red, the calculated "isoprene score" is shown next to each TPS. Among the calculated tomato TPSs, TPS47 has the highest isoprene score and is shown in bold. PaIspS, isoprene synthase from *Populus alba* (AB198180); EgIspS, isoprene synthase from *Eucalyptus globulus* (BAF02831.1); PmIspS, isoprene synthase from *Pueraria montana* (AY316691.1).

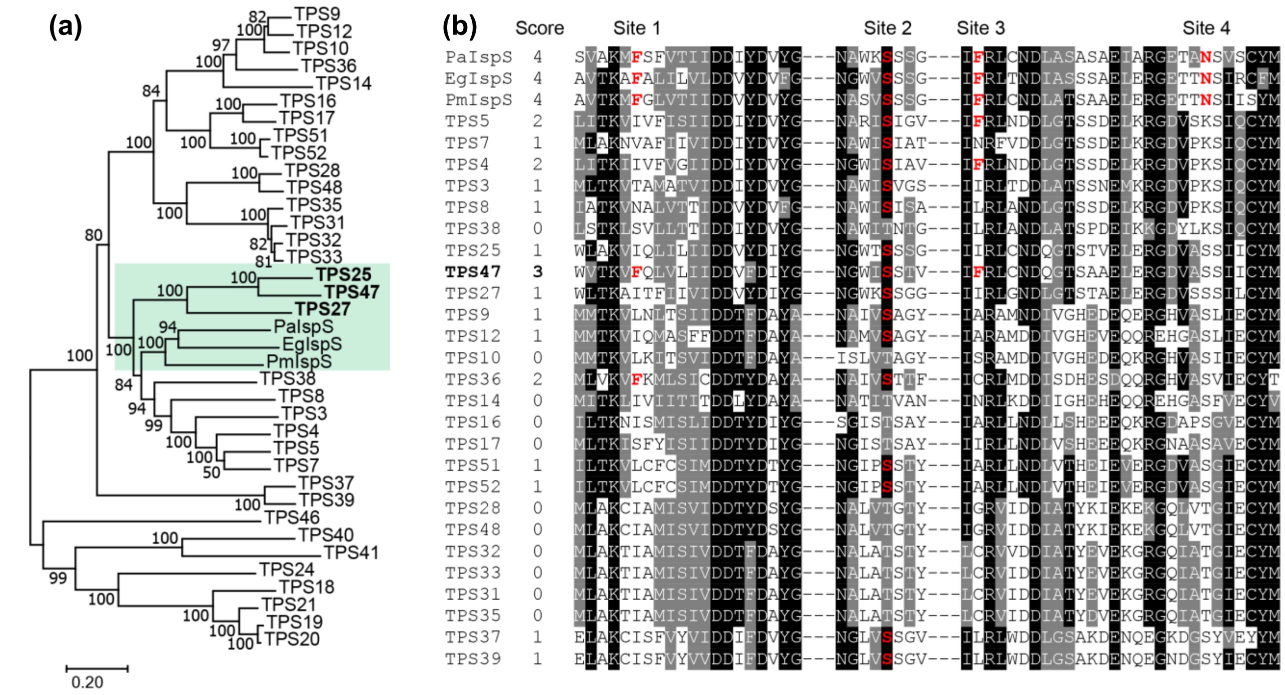

Fig. S7 Sequence alignment of the proteins encoded by the functional tomato TPS-c genes and CPS genes from other plants. Black shading indicates identical amino acid residues and grey shading are the conserved or semi-conserved substitutions observed and dashes indicate gaps. The conserved DxDD motif is underlined. The truncation sites for protein expression are indicated with triangle. AtCPS, copalyl diphosphate synthase from *Arabidopsis thaliana* (At4g02780); NtCPS2, 8-hydroxy-copalyl diphosphate from *Nicotiana tobacum* (HE588139.1); OsCPS1$_{ent}$, *ent*-copalyl diphosphate synthase 1 from *Oryza sativa* (Q6ET36.1); OsCPS2$_{ent}$, *ent*-copalyl diphosphate synthase 2 from *Oryza sativa* (Q6Z5I0.1); OsCPS3$_{syn}$, *syn*-copalyl diphosphate synthase 3 from *Oryza sativa* (Q0JF02.1); MtHPS, halimadienyl diphosphate synthase from *Mycobacterium tuberculosis* (NP_217894); SaCPS, copalyl diphosphate synthase from *Salinispora arenicola* (WP_012181499.1).

```
TPS40        1 ----------------------------------MSISASFLRFSLTAHYQPSPSSSPPNQPFKFLKSNREHVEFNRILQCHAVSRRRTKDYKEVQSGSL
AtCPS        1 ----------------------------------MSLQYHVLNSIPSTTFLSSTKTTISSSFLTISGSPLNVARDKSRSGSIHCSKLRTQEYINSQEVQH
TPS41        1 ---------------------------------------------MLVITSNYRLSCHIHRLKSPNFLLTQQAEFKRHGSRNWLFQTEG---LPPLSLNCAPN
NtCPS2       1 ----------------------------------------MQVIITSSHRFFCHHLHQLKSPTSLSAQKAEFKKHGPRNWLFQTEGSLLYKPVRLNCATS
OsCPS1ent    1 MIHLHSPPTAPAAFGGAGSADWRRRRRWSWSSSSRAPVAKGGHLRPCVWRRGGDDGGGEDHHADGGGGGGGGAAWRARATTAGVSSSSSTAKGLQANIIE
OsCPS2ent    1 ------------------------------------------------MQMQVLTAASSLPRATLLRPAAAEPWRQSFLQLQARPIQRPGIMLHCKAQLQ
OsCPS3syn    1 ---------------------------------------------MPVFTASFQCVTLFGQPASAADAQPLLQGQRPFLHLHARRRRPCGPMLISKSPPY
MtHPS        1 ----------------------------------------------------------------------------------------------------
SaCPS        1 -----------------------------------------------------------------------------------------------MSADL

                                                  ▼
TPS40       67 PVIKWDDIAEEVDVETHTLEVY-----DPS-SNEDHIDAIRSMLGSMG--DGEISVSAYDTAWVAMVKDVKG-----TETPQFPSSLEWIANNQLADGSW
AtCPS       67 DLPLIHEWQQLQGEDAPQISVG-----SNSNAFKEAVKSVKTILRNLT--DGEITISAYDTAWVALID--AG-----DKTPAFPSAVKWIAENQLSDGSW
TPS41       56 DASYFGNVSDLKSDHSNKYEE-------KDIQMRNLREEIKNMLSSMG--DGRSSVSPYDTAWVSFIEDTNTNINGTSKRPLFPSCLQWIIDNQLDDGSW
NtCPS2      61 DASYLGNVNEYLESDHSKNSEEKDIQVSRTIQMKGLTEEIKHMLNSME--DGRLNVLAYDTAWVSFIPNTTN--NGNDQRPMFPSCLQWIIDNQLSDGSW
OsCPS1ent  101 HETPRITKWPNESRDLDDHQQNNEADEEADDELQPLVEQVRSMLSSME--DGAITASAYDTAWVALVPRLDG-----EGGTQFPAAVRWIVGSQLADGSW
OsCPS2ent   53 GQETRERRQLDDDEHARPPQGGDDDVAASTSELPYMIESIKSKLRAARNSLGETTVSAYDTAWIALVNRLDGGGE---RSPQFPEAIDWIARNQLPDGSW
OsCPS3syn   56 PASEETREWEADGQHEHTDELR--------ETTTTMIDGIRTALRSIG--EGEISISAYDTSLVALLKRLDGGD-----GPQFPSTIDWIVQNQLPDGSW
MtHPS        1 -------------------------------------METFRTLLAKAALGNGISSTAYDTAWVAKLGQLDD--------ELSDLALNWLCERQLPDGSW
SaCPS        6 GASAPAAVEPVPAG------------------RADTAESLVAELIRVP--AGQVSPSLYETARLVSLAPWLTG---------HAERVRHLLTSQRPDGGW

TPS40      154 GDNSIFLVYDRVINTLACVIALKS------WNLHPDKILLGMSFMRENLSRIGDENAEHMPIGFEVAFPSLIEIAKKLGLD----FPYDSPVLQDIYASR
AtCPS      153 GDAYLFSYHDRLINTLACVVALRS------WNLFPHQCNKGITFFRENIGKLEDENDEHMPIGFEVAFPSLLEIARGINID----VPYDSPVLKDIYAKK
TPS41      147 GEELVFCIYDRLLNTLACVVALTL------WNTCLHKRNKGVMFIKENLRKLEGGEVVNMTSGFEFVFPSLLDKAQQLHIDN---IPYDAPVFRDIYARR
NtCPS2     157 GEEIVFCIYDRLLNTLVCVIALTL------WNTCLHKRNKGVMFIKENLSKLETGEVENMTSGFELVFPTLLEKAQQLDID----IPYDAPVLKDIYARR
OsCPS1ent  194 GDEALFSAYDRVINTLACVVALTR------WSLHHDQCKQGLQFLNLNLWRLAEEEPDTMPIGFEIAFPSLVEAARGLGID----FPYDHPALKGIYANR
OsCPS2ent  150 GDAGMFIVQDRLINTLGCVVALAT------WGVHEEQRARGLAYIQDNLWRLGEDDEEWMMVGFEITFPVLLEKAKNLGLD----INYDDPALQDIYAKR
OsCPS3syn  141 GDASFFMMGDRIMSTLACVVALKS------WNIHTDKCERGLLFIQENMWRLAHEEEDWMLVGFEIALPSLLDMAKDLDLD----IPYDEPALKAIYAER
MtHPS       56 GAEFPFCYEDRLLSTLAAMISLTSNKHRRRRAAQVEKGLLALKNLTSGAFEGPQLDIKDATVGFELIAPTLMAEAARLGLA----ICHEESILGELVGVR
SaCPS       77 GPPEGYALVPTVSATEALLAELRTAPAAEPLIRATDAGLTVLTRWLSAPRSLPDTPAIDLIVPALAAAINRHLVEADLPSALGHWRAAARLRLPAGMDDR

TPS40      244 QLKLTRIPKDIMHKVPTTLLHSLEGMT-DLDWQKLLQFQCTDGSFLFSPSSTAYALMQTQ----DHNCLNYLKNAVHKFNGGVPNVYPVDLFEHIWTVDR
AtCPS      243 ELKLTRIPKEIMHKIPTTLLHSLEGMR-DLDWEKLLKLQSQDGSFLFSPSSTAFAFMQTR----DSNCLEYLRNAVKRFNGGVPNVFPVDLFEHIWIVDR
TPS41      238 EVKFTRFPKDLIHTIPTIVLFSLEGLR-DLDWQRLLKLQMEDGSFLTSPSSTAIVFMNTN----DDKCFTFLQNAVQKFNGGVPCSYPADIQARLWAIDR
NtCPS2     247 EVKLTRIPKDVIHTIPTTVLFSLEGLRDDLDWQRLLKLQMPDGSFLISPASTAFAFMETN----DEKCLAYLQNVVEKSNG-GARQYPFDLVTRLWAIDR
OsCPS1ent  284 ELKLKRIPKDMMHIVPTSILHSLEGMP-GLDWQRLLKLQCSDGSFLFSPSATAYALMQTG----DKKCFAYIDRIIKKFDGGVPNVYPVDLFEHIWVVDR
OsCPS2ent  240 QLKLAKIPREALHARPTTLLHSLEGME-NLDWERLLQFKCPAGSLHSSPAASAYALSETG----DKELLEYLETAINNFDGGAPCTYPVDNFDRLWSVDR
OsCPS3syn  231 ERKLAKIPRDVLHSMPTTLLHSLEGMV-DLDWEKLLKLRCLDGSFHCSPASTATAFQQTG----DQKCFEYLDGIVKKFNGGVPCIYPLDVYERLWAVDR
MtHPS      152 EQKLRKLGGSKINKHITAAFSVELAGQDGVGMLDVDNLQETNGSVKYSPSASAYFALHVKP--GDKRALAYISSIIQAGDGGAPAFYQAEIFEIVWSLWN
SaCPS      177 RLAAVHGLIGAGAALPEKLLHALEVVG-SAAHGVRGVRPTRSGIVGASPAATAAWLGSPAGGHRHPGASAYLERVVRQHDALAPCATPITVFERAWVVAT

TPS40      339 LQRLGISR---YFELEIKECIDYVSRYWTN-KGICWARNSPVQDIDDTAMAFRLLRLHGYAVSADVFKHFESKGEFFCFVGQSN-QAVTGMYNLYRASHV
AtCPS      338 LQRLGISR---YFEEEIKECLDYVHRYWTD-NGICWARCSHVQDIDDTAMAFRLLRQHGYQVSADVFKNFEKEGEFFCFVGQSN-QAVTGMFNLYRASQL
TPS41      333 LQRLGISY---YFEEEIKDLLEYVFRYWDKENGFFSARNSNICEVDTTCMAIRLLRLHGFDVSPDVLHKFKDGDEFFCLRGESN-KSATVMFNLYRCSQA
NtCPS2     342 LQRLGISY---YFAEEFKELLNHVFRYWDEENGIFSGRNSNVSDVDDTCMAIRLLRLHGYDVSPDALNNFKDGDQFVCFRGEVD-GSPTHMFNLYRCSQV
OsCPS1ent  379 LERLGISR---YFQREIEQNMDYVNRHWTE-DGICWARNSNVKEVDDTAMAFRLLRLHGYNVSPSVFKNFEKDGEFFCFVGQST-QAVTGMYNLNRASQI
OsCPS2ent  335 LRRLGISR---YFTSEIEEYLEYAYRHLSP-DGMSYGGLCPVKDIDDTAMAFRLLRLHGYNVSSSVFNHFEKDGEYFCFAGQSS-QSLTAMYNSYRASQI
OsCPS3syn  326 LTRLGISR---HFTSEIEDCLDYIFRNWTP-DGLAHTKNCPVKDIDDTAMGFRLLRLYGYQVDPCVLKKFEKDGKFFCLHGESNPSSVTPMYNTYRASQL
MtHPS      250 LSRTDIDLSDPEIVRTYLPYLDHVEQHWVRGRGVGWTGNSTLEDCDTTSVAYDVLSKFGRSPDIGAVLQFEDADWFRTYFHEVG----------------
SaCPS      276 LARAGLAVT--QAPDLIPGLIADLTSVGTC------AGPGLPPDADTTAVTLYALAHLGFSVDLECLWRYETPDGFCTWPGEDG----------------
                                                          DxDD
```

```
TPS40      434 MFSGEK-ILENAKIFTSNYLREKRAQNQLLDKWIITKDLPGEVGYALDVPWYASLPRLETRFFLEHYGGEDDVWIGKTLYRMPLVNNSLYLELAKSDYNN
AtCPS      433 AFPREE-ILKNAKEFSYNYLLEKREREELIDKWIIMKDLPGEIGFALEIPWYASLPRVETRFYIDQYGGENDVWIGKTLYRMPYVNNNGYLELAKQDYNN
TPS41      429 LFPGEI-ICEEAKNFTYNFLHQYLANNQSKDKWVIAKDIPGEIRYALEFQWYASLPRVESRLYIDQYGGADEIWISKTLYRMPDVSNNVYLEAAKLDYNR
NtCPS2     438 LFPGEK-ILEEAKNFTYNFLQQCLANNRCLDKWVIAKDIPGEIWYALEFPWYASLPRVEARYYIEQYGGADDIWIGKTLYRMPDVNNNVYLQAAKLDYNR
OsCPS1ent  474 SFPGED-ILQRARNFSYEFLREREAQGTLHDKWIISKDLPGEVQYTLDFPWYASLPRVEARTYIGQYGGNDDVWIGKTLYRMPIVNNATYLELAKQDFNR
OsCPS2ent  430 VFPGDDDGLEQLRAYCRAFLEERRATGNLMDKWVIANGLPSEVEYALDFPWKASLPRVETRVYLEQYGASEDAWIGKGLYRMTLVNNDLYLEAAKADFTN
OsCPS3syn  422 KFPGDDGVLGRAEVFCRSFLQDRRGSNRMKDKWAIAKDIPGEVEYAMDYPWKASLPRIETRLYLDQYGGSGDVWIGKVLHRMTLFCNDLYLKAAKADFSN
MtHPS      334 --------------------------------------------------------------------------------PSISTNVHVLGALKQAGYDK
SaCPS      352 -------------------------------------------------------------------------FSLSTNAHVLDVVGLILTTDPGADRRH

TPS40      533 CQALHQFEWRRIRKWYYECGLGEFG-----LSEKRLLVTYYLGSASIFEAQRSTERMAWVKTAALMDCVRSCFGSPQVSAAAFLCEFAHYSSTALNSRYN
AtCPS      532 CQAQHQLEWDIFQKWYEENRLSEWG-----VRRSELLECYYLAAATIFESERSHERMVWAKSSVLVKAISSSFGESSDSRRSFSDQFHEYIANARRSDHH
TPS41      528 CQSQHRFEWLIMQEWFEKGNFQKFG-----ISKKEVLVSFFLAASSIFEVEKSRQRLAWAKSCILCKMITSYINQEATTWN---SFLMEFKNYRDMSIKK
NtCPS2     537 CQSQHRFEWLIMQEWFEKCNFQQFG-----ISKKYLLVSYFLAAASIFEVEKSRERLAWAKSRIICKMITSYYNDEATTWTTRNSLLMEFKVSHDPTRKN
OsCPS1ent  573 CQALHQHELQGLQKWFIENGLEAFG-----MTPEDVLRAYFLAAACIFEPNRASERLAWARVSVLANTISRHFYSDMSSMKRMERFMWSSLYEENGNVLG
OsCPS2ent  530 FQRLSRLEWLSLKRWYIRNNLQAHG-----VTEQSVLRAYFLAAANIFEPNRAAERLGWARTAILAEAIASHLRQYSANGAADGMTERLISGLASHDWDW
OsCPS3syn  522 FQKECRVELNGLRRWYLRSNLEKFGGT---DPQTTLMTSYFLASANIFEANRAAERLGWARVALLADAVSSHFRRIGGPKNSTSNLEELISLVPFDDAYS
MtHPS      354 CHPRVRKVLEFIRSSKEPGRFCWRDKW---HRSAYYTTAHLICAASNYDDALCSDAIGWILNTQRPDGSWGFFDGQATAEETAYCIQALAHWQRHSG---
SaCPS      379 VTAARRLADALRQRQQADGSWQDRWHASPYYATMCCALALAGFPGPGTAVTSLARAASWIVDTQRANGSWGRWKGTVEETAYAVQVLATVGRG-------

TPS40      628 TEDR--------------LVGVILGTLNHLSLSALLTHG--RDIHHYLRHAWENWLLTVGEGEGEGEGG--------------------AELIIRTLNLC
AtCPS      627 FNDRNMRLDRPGSVQASRLAGVLIGTLNQMSFDLFMSHG--RDVNNLLYLSWGDWMEKWKLYGDEGEGE--------------------LMVKMIILMKN
TPS41      620 SNET----------KEIIVLNNLCQFLHQLTKETYQDLG--KDIHHQLHNVWEEWLEENN---TTCQEA--------------------AVLLVQTINLS
NtCPS2     632 GNET----------KEILVLKNLRQFLRQLSEETFEDLG--KDIHHQLQNAWETWLVFLREEKNACQEE--------------------TELLVRTINLS
OsCPS1ent  668 LEGYAKDG---------ILARTLCQLIDLLSQETPPVREGQKCIHNLIRCAWIEWMMQQINMKDGRYDKGRVMHPGSCTVHNKETCLLIAQIVEICAGRI
OsCPS2ent  625 RESK-----------------DSAARSLLYALDELIDLHAFGNASDSLREAWKQWLMSWTNE--SQGSTGG---------------DTALLLVRTIEICS
OsCPS3syn  619 G---------------------------------------------SLREAWKQWLMAWTAKESSQESIEG---------------DTAILLVRAIEIFG
MtHPS      448 ----------------------------------------------------------------------------------------------------
SaCPS      472 ----------------------------------------------------------------------------------------------------

TPS40      692 SVHWISEEILLSHPTYQKLLEITNRVSHRLR---LYKGHSEKQVGMLTFSEEIEGDMQQLAELVLSHSDASELDANIKDTFLTVAKSFYYSAYCDDRTIN
AtCPS      705 NDLTN----FFTHTHFVRLAEIINRICLPRQ---YLKARRNDEK-----EKTIKSMEKEMGKMVELALSESDTFRDVSITFLDVAKAFYYFALCGD-HLQ
TPS41      685 S-GHMTHDEILS--------KYTNKVCHMLN---EFQN---DQICNSSKARDIELHMQALVKLVFSNTSSNNINQGIEDTYFKVVKTFYYTAHVSEETIN
NtCPS2     700 G-GYMTHDEILFDADYENLSNLTNKVCGKLN---ELQN---DKVTGGSKNTNIELDMQALVKLVFGNTSSN-INQDIKQTFFAVVKTFYYSAHVSEEIMN
OsCPS1ent  759 EEAASMINNTEGSWFIQLASSICDSLHAKML---LSQDTKKNETTINQIDKEIELGMQELAQYLLPRVDDRRINNKTKQTFLSIVKSCYYAANCSPHMLD
OsCPS2ent  691 GRHGSAEQSLKNSADYARLEQIASSMCSKLATKILAQ-NGGSMDNVEGIDQEVDVEMKELIQRVYGSS-SNDVSSVTRQTFLDVVKSFCYVAHCSPETID
OsCPS3syn  659 GRHVLTGQRPD-LWEYSQLEQLTSSICCKLSRRVLAQENGESTEKVEEIDQQVDLEMQELTRRVLQG--CSAINRLTRETFLHVVKSFCYVAYCSPETID
MtHPS      448 ---------------------------------------------------------TSLSAQISRAGGWLSQHCEPPYAPLWIAKTLYCSATVVKAAIL
SaCPS      472 ------------------------------------------------------RPGADEAIRRGRAYLTEGTTAHDPGPPLWHDKDLYRPAMIVRAAVV

TPS40      789 FHIAKVLFERVV--
AtCPS      792 THISKVLFQKV---
TPS41      770 NHISKVLFQKA---
NtCPS2     792 FHISKVLFQQV---
OsCPS1ent  856 QHISEVIFEQVI--
OsCPS2ent  789 GHISKVLFEDVN--
OsCPS3syn  756 SHIDKVIFQDVI--
MtHPS      491 SALRLVDESNQ---
SaCPS      518 AARHLAGAAGPATA
```

Fig. S8 Predictions of subcellular localization for TPS and prenyltransferase proteins made by TargetP 1.1 (a) and ChloroP 1.1 (b).

(a)

targetp v1.1 prediction results
Number of query sequences: 42
Cleavage site predictions not included.
Using PLANT networks.

Name	Len	cTP	mTP	SP	other	Loc	RC
TPS3	607	0.781	0.218	0.044	0.036	C	3
TPS4	590	0.756	0.205	0.040	0.055	C	3
TPS5	609	0.563	0.315	0.087	0.018	C	4
TPS7	592	0.499	0.155	0.233	0.054	C	4
TPS8	597	0.375	0.099	0.136	0.546	_	5
TPS9	548	0.050	0.182	0.180	0.693	_	3
TPS10	556	0.166	0.244	0.083	0.313	_	5
TPS12	548	0.053	0.301	0.139	0.461	_	5
TPS14	568	0.113	0.093	0.089	0.861	_	2
TPS16	553	0.075	0.144	0.059	0.858	_	2
TPS17	554	0.091	0.149	0.063	0.774	_	2
TPS18	773	0.024	0.900	0.009	0.207	M	2
TPS19	778	0.612	0.323	0.014	0.133	C	4
TPS20	778	0.612	0.323	0.014	0.133	C	4
TPS21	784	0.661	0.215	0.022	0.122	C	3
TPS24	820	0.930	0.035	0.075	0.070	C	1
TPS25	547	0.272	0.086	0.089	0.713	_	3
TPS27	554	0.214	0.166	0.081	0.576	_	4
TPS28	554	0.169	0.129	0.125	0.725	_	3
TPS31	555	0.243	0.057	0.145	0.727	_	3
TPS32	548	0.113	0.199	0.078	0.627	_	3
TPS33	552	0.171	0.123	0.193	0.514	_	4
TPS35	556	0.211	0.073	0.142	0.692	_	3
TPS36	591	0.548	0.061	0.144	0.293	C	4
TPS37	560	0.547	0.206	0.055	0.092	C	4
TPS38	546	0.157	0.177	0.046	0.816	_	2
TPS39	563	0.055	0.151	0.252	0.584	_	4
TPS40	800	0.921	0.093	0.038	0.044	C	1
TPS41	780	0.077	0.730	0.022	0.145	M	3
TPS46	821	0.231	0.055	0.112	0.891	_	2
TPS47	562	0.096	0.239	0.083	0.649	_	3
TPS48	560	0.327	0.105	0.160	0.528	_	4
TPS51	550	0.054	0.154	0.074	0.876	_	2
TPS52	551	0.038	0.359	0.059	0.720	_	4
FPPS1	342	0.027	0.053	0.378	0.864	_	3
SSU1	315	0.019	0.278	0.057	0.600	_	4
SSU2	334	0.366	0.190	0.170	0.100	C	5
GGPPS3	360	0.505	0.132	0.010	0.423	C	5
TPT1	372	0.158	0.866	0.013	0.029	M	2
TPT2	373	0.178	0.785	0.009	0.053	M	2
GGPPS2	363	0.322	0.082	0.028	0.170	C	5
GGPPS1	365	0.823	0.085	0.074	0.119	C	2
cutoff		0.000	0.000	0.000	0.000		

(b)

chlorop v1.1 prediction results
Number of query sequences: 42

Name	Length	Score	cTP	CS-score	cTP-length
TPS3	607	0.564	Y	2.430	28
TPS4	590	0.550	Y	-0.472	38
TPS5	609	0.523	Y	-0.094	42
TPS7	592	0.538	Y	5.945	45
TPS8	597	0.443	-	-1.415	8
TPS9	548	0.437	-	1.897	2
TPS10	556	0.486	-	1.526	4
TPS12	548	0.446	-	3.181	38
TPS14	568	0.436	-	7.857	68
TPS16	553	0.428	-	7.547	9
TPS17	554	0.427	-	1.932	9
TPS18	773	0.454	-	0.863	28
TPS19	778	0.545	Y	8.925	36
TPS20	778	0.545	Y	8.925	36
TPS21	784	0.528	Y	5.963	36
TPS24	820	0.548	Y	4.184	53
TPS25	547	0.443	-	-0.394	8
TPS27	554	0.446	-	-0.085	33
TPS28	554	0.433	-	-2.312	16
TPS31	555	0.433	-	4.268	3
TPS32	548	0.438	-	-0.872	12
TPS33	552	0.442	-	6.280	3
TPS35	556	0.436	-	6.941	3
TPS36	591	0.485	-	3.693	41
TPS37	560	0.505	Y	8.499	30
TPS38	546	0.432	-	-2.033	13
TPS39	563	0.444	-	-0.156	86
TPS40	800	0.548	Y	2.548	46
TPS41	780	0.462	-	-0.630	26
TPS46	821	0.446	-	-1.743	13
TPS47	562	0.436	-	5.568	61
TPS48	560	0.469	-	0.624	42
TPS51	550	0.427	-	1.403	29
TPS52	551	0.428	-	2.759	34
FPPS1	342	0.432	-	1.534	12
SSU1	315	0.439	-	7.229	22
SSU2	334	0.512	Y	5.161	17
GGPPS3	360	0.567	Y	5.148	65
TPT1	372	0.506	Y	2.832	13
TPT2	373	0.507	Y	3.003	13
GGPPS2	363	0.528	Y	4.003	63
GGPPS1	365	0.553	Y	3.695	43

Fig. S9 Phylogenetic analysis of tomato TPT homologs and TPTs identified in other plants. The maximum likelihood phylogenetic tree was constructed using MEGA7 with default seetings. The first two letters in each of the homologs are abbreviations for the species: Aa, *Artemisia annua*; Ag, *Abies grandis*; At, *Arabidopsis thaliana*; Am, *Antirrhinum majus*; Cb, *Clarkia breweri*; Cr, *Catharanthus roseus*; Hb, *Hevea brasiliensis*; Hl, *Humulus lupulus*; La, *Lupinus albus*; Mi, *Mangifera indica*; Mp, *Mentha piperita*; Nt, *Nicotiana tobacum*; Os, *Oryza sativa*; Pa, *Picea abies*; Sa, *Sinapis alba*; Vv, *Vitis vinifera*; Zm, *Zea mays*. The GenBank accession numbers of the proteins used in the phylogenetic analysis can be found in Methods S1. The tomato TPT homologs are shown in bold. TPT1, TPT2 and the four Arabidopsis GFPP synthases are shaded in red.

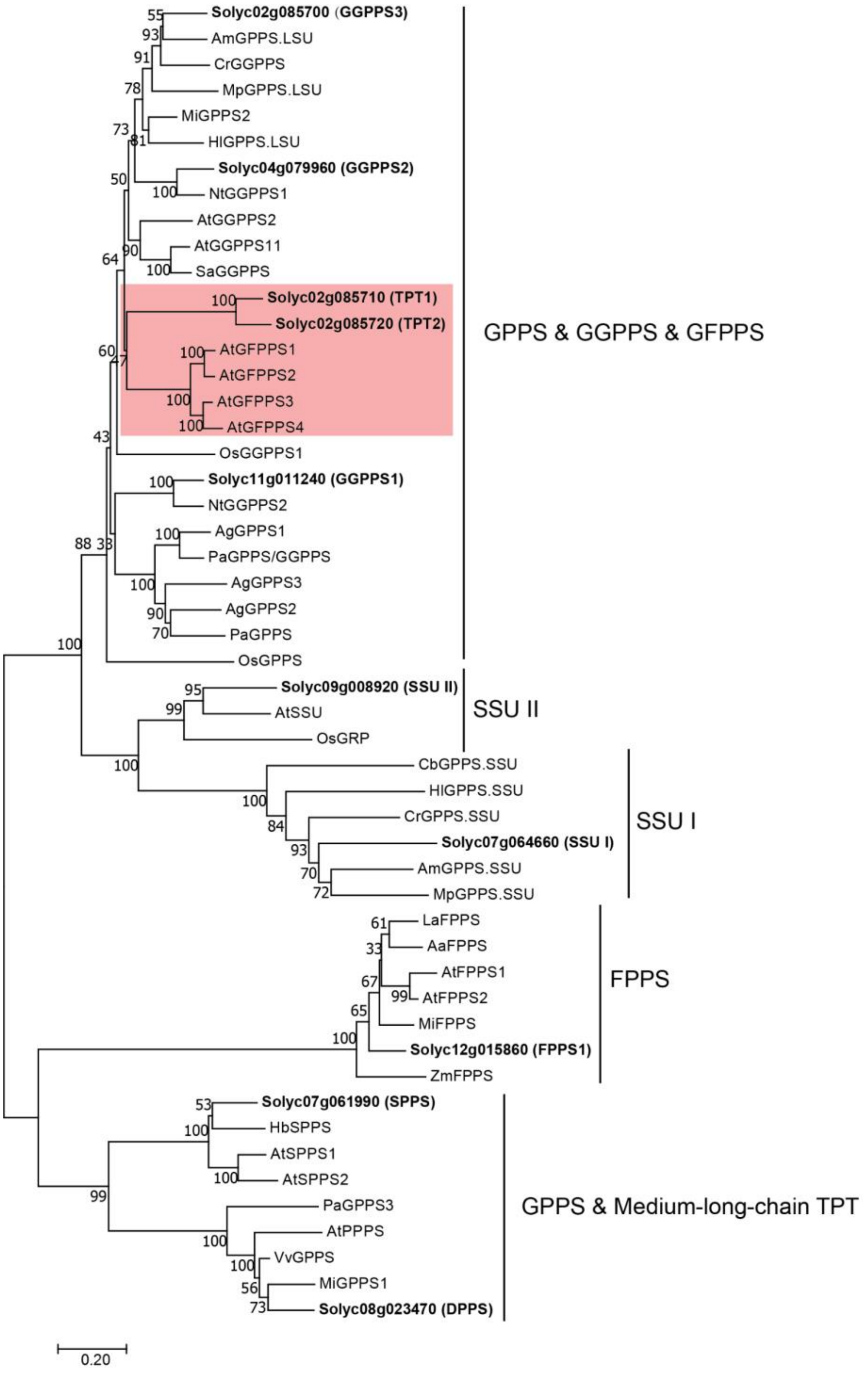

Fig. S10 GC-MS analysis of the products formed in planta by transiently co-expressing TPS with TPT genes in *Nicotiana benthamiana* leaves. Transit peptide truncated versions ("d") of all tested genes were transiently expressed in tobacco cytosol, co-expression of *AtGFPPS2* with *AtTPS19* was used as a positive control to produce the sesterterpene (-)-retigeranin B (Shao *et al*., 2017).

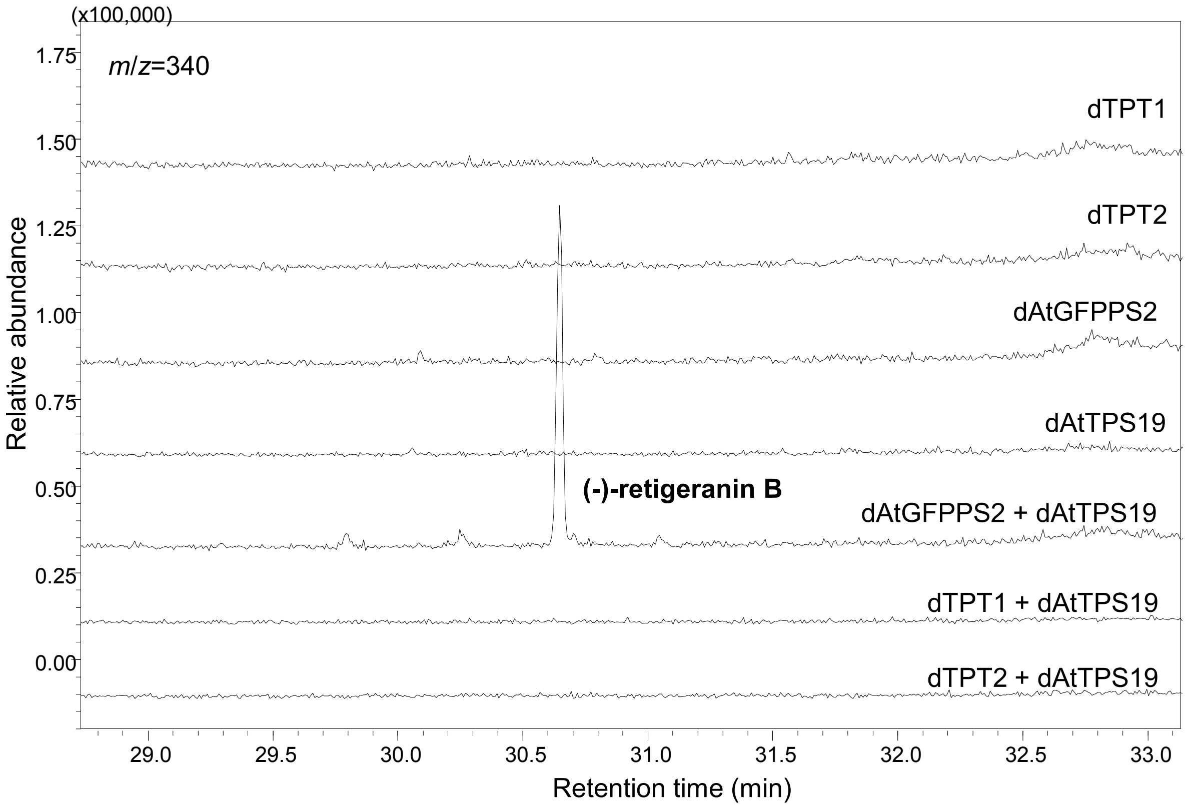

Fig. S11 Sequence alignment of five Arabidopsis sesterterpene synthases and tomato TPSs from TPS-a, TPS-b and TPS-g clades. (a) Key residues determining the substrate specificity of sesterterpene synthases are shown in red, the corresponding residues of tomato TPSs are shown in blue. Black shading indicates identical amino acid residues and grey shading are the conserved or semi-conserved substitutions observed and dashes indicate gaps. (b) Amino acids with large side chain (shaded in red) from tomato TPSs. (c) Amino acids with small side chain (shaded in yellow) from Arabidopsis sesterterpene synthases.

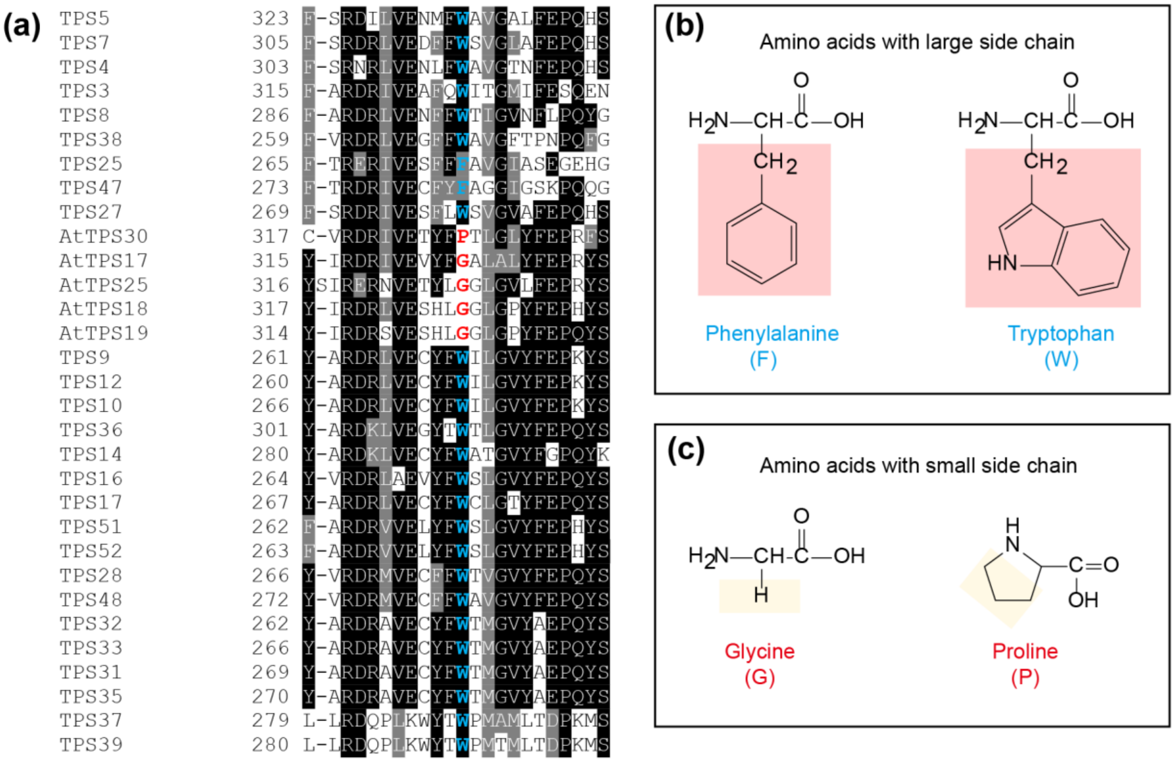

Fig. S12 Sequence alignment of the proteins encoded by the functional tomato TPS-a genes. Black shading indicates identical amino acid residues and grey shading are the conserved or semi-conserved substitutions observed and dashes indicate gaps. The conserved R(R,P,H,L)$(x)_8$W and DDxxD motifs are underlined. The truncation sites for protein expression in *E. coli* are indicated with triangle.

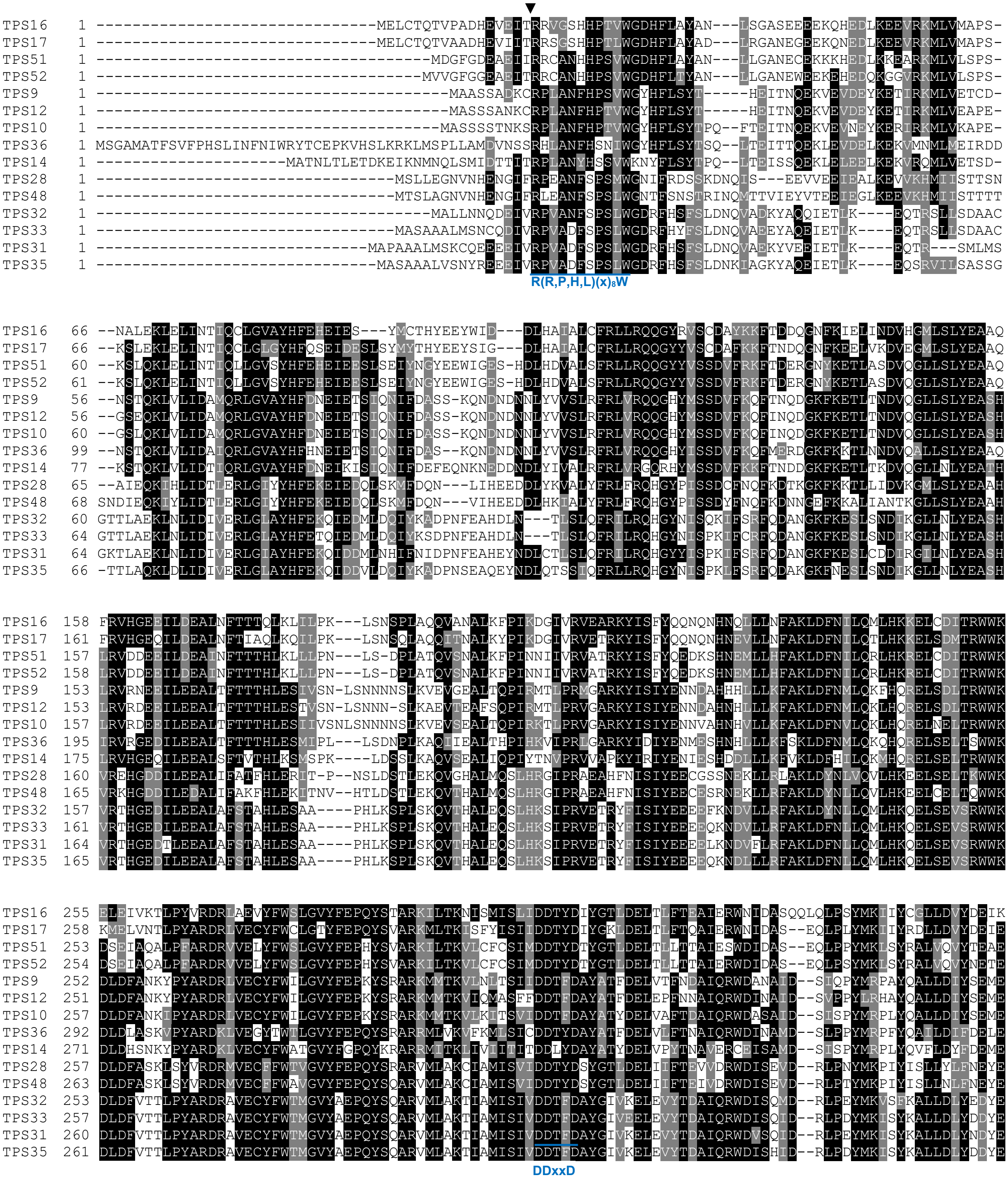

```
TPS16  355 KDLANE--NKSFLINYSIIEMKKMVMAYFQEAKWYY-GKTIPKMEEYMKSGISTSAYVQVATTSWLGMGNVATKDSFDWIVNEPPILVASSIIARLLNDL
TPS17  356 KELANE--NKSFLVNYSINEMKKVVRGYFQEAKWYY-GKKVPTMEQYMKNGISTSAYILLTTTSWLAMGNVATKDAFDWVATEPPIVVASCYIIRLLNDL
TPS51  351 RELENLGNKMTFRVKYSINEMKKLLRAYFQEAKWYH-EKVVPTMEQYIKNGIPSSTYLLLATTSWLGMGDVATKDAFDWISNEPTILVALSIIARLLNDL
TPS52  352 KELENLGNKMTYRVKYSINEMKKLLRAYFQEAKWYH-GKDVPTMEQYIKNGIPSSTYLLLATTSWLGMGDVATKDAFDWISNEPTILVASSIIARLLNDL
TPS9   350 QVLSKEG--KLDRVYYAKNEMKKLVRAYFKETQWLNDCDHIPKYEEQVENAIVSAGYMMISTTCLVGIEEFISHETFEWLMNESVIVRASALIARAMNDI
TPS12  349 QALAKEF--KSDRVYYAKYEMKKLVRAYFKEAQWLNNDNHIPKYEEHMENAMVSAGYMMGATTCLVGVEEFISKETFEWMINEPLIVRASSLIARAMDDI
TPS10  355 QVLSNEG--KLDRVYYGKHEIKKIVRAYFKEAQWLNDANYIPKYEEHMEISLVTAGYMMGATNCLVGVEEFISKDTFEWLKNEPLIVRAASLISRAMDDI
TPS36  390 EELTKEG--KSDRVYYGKFEMKKLARAYFKEAQWLN-AGYIPNCDEYIKNAIVSTTFMALGTTSLIGMEEFITKDIFEWITNEPSILRASSTICRLMDDI
TPS14  369 EELTKDG--KAHYVYYAKVEMNKLIKSYLKEAEWLK-NDIIPKCEEYKRNATITVANQMILITCLIVAGEFISKETFEWMINESLIAPASSLINRLKDDI
TPS28  355 REINE--QDRFNGVNYVKEAMKEIVRSYYIEAEWFI-EGKIPSFEEYLNNALVTGTYYLLAPASLLGMESTS-KRTFDWMMKKPKILVASAIIGRVIDDI
TPS48  361 IEIELE-QDRFNGVHYVKEAMKEIVKSYYIEAEWFL-EGKIPSFKEYLGNALVTGTYYLLGPASLLGMKLAS-KRTFDWMMNKPKILVASAIIGRVIDDI
TPS32  351 KELSK--DGRSDVVQYAKERMKEIVRNYFVEAKWFI-EGYMPPVSEYLSNALATSTYYLLTTTSYLGVKSAT-KEDFEWLAKNPKILEANVTLCRVVDDI
TPS33  355 TELSK--DGRSDVVHYAKERMKEIVRNYFVEAKWFI-EGYMPPVSEYLSNALATSTYYLLTTTSYLGVKSAT-KEDFEWLAKNPKILEANVTLCRVIDDI
TPS31  358 TELSN--DGRSDVVQYAKERMKEIVRNYFVEAKWFI-EGYMPPVSEYLSNALATSTYYLLTTTSYLGMKSAT-KKDFEWLAKNPKILEANVTLCRVIDDI
TPS35  359 TELSK--DGRSDVVHYAKERMKEIVRNYFVEAKWFI-EGYMPPVSEYLCNALATSTYYLLTTTSYLGVKSAN-KEDFEWLAKNPKILEANVTLCRVIDDI

TPS16  452 LSHEEEQKRGDAPSGVECYMKEYGVTKEEAHIKIRNTIENSWKDLYEEYFKVNGTIIPRVLLMCIINLARVIEFIYK-DEDAYTFPKNNLKDVIYRILID
TPS17  453 VSHEEEQKRGNAASAVECYMNEYSVTKEEAHIKIRDIIENYWKDLNEEYFKVDMIIIPRVLLMCIINLTRVAEFIYK-DEDAYTFSKNNLKDVISDILVD
TPS51  450 VTHEIEVERGDVASGIECYMNEYGATKEEAYMETRKIIENNWKVLNRGCLKP--TTVPRVLLMPVLNLTRVAEFFYK-DDDAYTFSTNNLKDIISLVLID
TPS52  451 VTHEIEVERGDVASGIECYMNEYGATKEEAYMEIRKIIENNWKDLNRGCLKP--TTVPRVLLMPVLNLTRVAEFFYK-DEDAYTFSKNNLKDVISMVLID
TPS9   448 VGHEDEQERGHVASLIECYMKDYGASKQETYIKFLKEVTNAWKDINKQFFRP--TEVPMFVLERVLNLTRVADTLYK-EKDTYTNAKGKLKNMINSILIE
TPS12  447 VGHEVEQQREHGASLIECYMKDYGVSKQEAYVKFQKEVTNGWMDINREFFCPD-VEVPKFVLERVLNFTRVINTLYK-EKDEYTNSKGKFKNMIISLLVE
TPS10  453 VGHEDEQKRGHVASIIECYMKEYGASKQEAYAKFKKEVTNVWKDINKEFFRP--TEVPMFVLERALNFARVIDTLYQ-EVDGYTNSKGLLKDLVNSLLIE
TPS36  487 SDHESDQQRGHVASVIECYTKEYGASKQEAYVKFRKEVKDAWKGINKALLRP--IEVPIFVLQRILNLARTMDTFFQDEEDGYTNSNSKCKDIVTLLLVD
TPS14  466 IGHEHEQQREHGASFVECYVKEYRASKQEAYVEARRQIANAWKDINTDYLHA--TQVPTFVLQPALNLSRLVDILQE---DDFTDSQNFLKDTIKLLFVD
TPS28  451 ATYKIEKEKGQLVTGIECYMQENNLSVEKASAQLSEIAESAWKDLNKECIKTTTSNIPNEILMRVVNLTRLIDVVYKNNQDGYSNPKNNVKSVIEALLVN
TPS48  458 ATYKIEKEKGQLVTGIECYMEENNLSVEEASAQFSEIAENAWKDLNKECIKSTDS-MPTEILMRVVNLTRLIDVVYKNNQDGYSDPKNNVKAVIEALLVN
TPS32  447 ATYEVEKGRGQIATGIECYMRDYGVSTQVAMDKFQEMAEIAWKDVNEGILR--PTPVSTEILTRILNLARIIDVTYKHNQDGYTHPEKVLKPHIIALLVD
TPS33  451 ATYEVEKGRGQIATGIECYMRDYGVSTQVAMEKFQEMAEIAWKDVNEGILR--PTPVSTEILTRILNLARIIDVTYKHNQDGYTHPEKVLKPHIIALLVD
TPS31  454 ATYEVEKGRGQIATGIECYMRDYGVSTQVAMDKFQEMAETAWKDVNEGILR--PTPVSAKILTRILNLARIIDVTYKHNQDGYTHPEKVLKPHIIALLVD
TPS35  455 ATYDVEKGRGQIATGIECYMRDYGVSTEEAMEKFEEMAEIAWKDVNEGILR--PTPVSTEILTRILNLARIIDVTYKHNQDGYTHPEKVLKPHIIALLVD

TPS16  551 PII-----
TPS17  552 PII-----
TPS51  547 PITT----
TPS52  548 PIKA----
TPS9   545 SVKI----
TPS12  545 SVEI----
TPS10  550 SVKISIS-
TPS36  585 SVTIGRS-
TPS14  561 SVNSTSCG
TPS28  551 PINM----
TPS48  557 SIKL----
TPS32  545 SIEI----
TPS33  549 SVEI----
TPS31  552 SIEI----
TPS35  553 SIEI----
```

Fig. S13 Sequence alignment of the proteins encoded by the functional tomato TPS-b and TPS-g genes. Black shading indicates identical amino acid residues and grey shading are the conserved or semi-conserved substitutions observed and dashes indicate gaps. The conserved RR(x)$_8$W and DDxxD motifs are underlined. The truncation sites for protein expression in *E. coli* are indicated with triangle.

```
                                                                       ▼
TPS25   1 --------------------------MACINMVSIASTMQTQKLHSTTEK--DSQPERISTYKPNIWKYDHLLSLTNQYSEAKYKIE-AEKLKEEVG---
TPS47   1 --------------------------MACRCILSVIPTMQTQKIHTLSIKNQIQPETTISSYKPNIWKYDDLLSLTTQYSKRKYQIE-AEKLKEEVIS--
TPS27   1 --------------------------MTSEQQSVFCNQIHSTTSFRKSNIDETLIQRRNANYKPNIWKYDILQSLKSQYSECKYKKE-AQKLKEEFLW--
TPS5    1 ---------MVSILSNIGMMVVTFKRPSLFTSLRRRSANN---IIITKHSHPISTTRRSGNYKPTMWDFQFIQSLHNPYEGDKYMKR-LNKLKKEVKK-M
TPS7    1 ---------MVSIFSNAGMMMVTFNRPSFTCFSSLHHYSISARGAINNISTPISATRRSGNYKPTMWDFQFIQSLHNPYEGDKYMKR-LNELKKEVKK-M
TPS4    1 ---------MKAILLNNIGVLSSRPPRATCLFSINGGKPSSLIVVSKASSPNPTTIRRSGNYKPTMWDFQFIQSVNNLYAGDKYMER-FDEVKKEMKKNL
TPS3    1 MSIFSTRYLVTPFSSFSPPKAFVSKACSLSTGQPLNYSPNISTNIISSSNGIINPIRRSGNYEPTMWNYEYIQSTHNHHVGEKYMKR-FNELKAEMKKHL
TPS8    1 -------------------------MYKLEMTMSISKSNLISKLEVPKSCISNVPIRRSGNYQPSIWDYNHIQSLKNHYSDEKFMRR-RNELKMEVKI--
TPS38   1 ---------------------------------------------MLQSCISTMDIRRSGNYKPSIWEDGYVQSRPNLYAEEKYCER-AEKLKEEVRK--
TPS37   1 --------------------------------MANITKAFSPLPLYLCQIGSKRSSIKVSCRSSNRWNFQEDLLKKTSYLQTSYNRDGFNSTKFGLLVKD
TPS39   1 ---------------------------MEMTKVLISPSQYLSMHIISGNIIQNERSIQVSCKSSNKWAVQEDLLRAT----STYNQDGFDSTKFGLLMKD
                                                                   RR(x)8W

TPS25  69 --CMFSNTTSPVAQLQLIDGIDKLGLSAYFEVDTKETLENIILYMKTSSTS--KDLYATALCFRLLREHGYHASQDMLKD-LFDGKGK-----LPLDMK-
TPS47  72 --CTFSKSDTPVVLLELVDEIDKFGLTDYFDVETKAALEKTITYMKCSNTKDNKDIYATALCFRLLRQHGYYASQDMLKE-LFDRKK-------MSDTK-
TPS27  72 --VVAEIEN-PLAKLELIDSINKMALSHLFDKEIMVFLQN---MEKLKDSDNEMDLYSTALYFRIFRQYGYNVTQDVFLS-YMDEMGEKINVDTNMDPK-
TPS5   87 MMTVEGSHDEELEKLELIDNLERLGVSYHFKDEIMQIMRSINININ---IAPPDSLYTTALKFRLLRQHGFHISQDILND--FKDENGNLKQSICKDTKD
TPS7   90 MMTVEGSHDEELEKLELIDNLERLGVSYHFKDEIMQILRSINININ---IAPPDSLYTTSLKFRLLRQHGFHISQDVLKD--FKDENGNLKQSICKDT--
TPS4   91 MMMVEGLIEELDVKLELIDNLERLGVSYHFKNEIMQILKSVHQQIT---CRD-NSLYSTALKFRLLRQHGFHISQDIFND--FKDMNGNVKQSICNDT--
TPS3  100 MMMLH-EESQELEKLELIDNLQRLGVSYHFKDEIIQILRSIHDQSSS-EATSANSLYYTALKFRILRQHGFYISQDILND--FKDEQGHFKQSLCKDT--
TPS8   73 --MLSDRNMKQLEQLEIIDNLQRLGLSYHFEDEIYSILNNLSDKGSK-R----DHLYAKALEFRLLRQHGFNIVSQETFGGFYDNTTGFGEIHHNEDT--
TPS38  53 --MLQKRMTNSLEQLELVDILQRLGIYYHFEEEIDTVLKQIYVNYNK-RDHHNEELYDTALEFRLLRQHGYHLPQEIFCS--FMNEEGKFKTALVEDT--
TPS37  69 VKYALRTQINNNNNLVLVDTLQRMGIEHHFQQEIQSILQKEYEQNTC--FLKYQNHHDISLCFRLLRQEGYHVSADVFKK-LKNNDDGTFGLNLNQDVN-
TPS39  70 VKYALRTQINNNNNLVLVDTLQRMGIEHHFQQEIQSILQKEYEQNTC--FLKYQNHHDISLCFRLLRQEGYHVSADVFKK-LKNNDDGTFGLNLNQDVN-

TPS25 158 ---------------------TSLELFEGSHLSIDGEN---------------LLNDIRLFSTKNLKNLSLDVDRLT-SNPLAWRVRWYDVRKHIITAQN
TPS47 161 ---------------------TLLELLEGSYLGMDGEN---------------LLNDIRLLTTNNLMNTSFNKENLSNYFPLAWRVRWYDVRGHITMFTQ
TPS27 164 ---------------------TMMQLFEASHLALKDEN---------------MLDEARIFCTNNLKNIIP------MEMPLHWKVEWYNTREHISKQAN
TPS5  182 ILNSSKDEHDNLKQSTCNNTKGLLKLYEASFLSIENES--FLRNTTKSTLAHLMRYVDQNRCG-EEDNMIVELVVHALELPRHWMVPRLETRWYISIYER
TPS7  183 --------------------KGMLELYEASFLSTETEN--TLKSATRFTMSHLKNYVDNHSCGNQDDDIIVELVVHALELPRHWMMPKLETEWYIRIYGR
TPS4  183 --------------------KGLLELYEASFLSTECET--TLKNFTEAHLKNYVYIN--HSCGDQYNNIMMELVVHALELPRHWMMPRLETRWYISIYER
TPS3  194 --------------------KGLLQLYEASFLSTKSETSTLLESANTFAMSHLKNYLN---GGDEENNWMVKLVRHALEVPLHCMMLRVETRWYIDIYEN
TPS8  164 --------------------KGMLYLYEASFLAIEGEK---ELELARNLTEEHLREYLADQNKNDVDQNLVELVHHALELPLHWRMLRLETKWFINYYKK
TPS38 146 --------------------KGLLSLYEASYLCMEDEN---IMENAR--------DFATHYLMENVKKKMDEQVSHALEMPVHWRMERLEARWFIEIYHK
TPS37 165 ---------------------GLIGLYEASQLGVEGEY----------ILDEIAKFSGDHLNACLANSDEARIIKETLKYPYHKSLSRWKNKSFINNFKG
TPS39 166 ---------------------GLIGLYEASQLGVEGEY----------ILDEIAKFSGDHLNACLVNSDEARIIKETLKYPYHKSLSRWKAKSFINNFKG

TPS25 221 ----CNDTNPMLLKLAKLNFNIIQATHQKDLKDVIRWWRNVSIIENLEFTRERIVESFFFAVGIASEGEHG-SMRKWLAKVIQLILIIDDVYDIYGTLAD
TPS47 225 QGNNNNNTNQVLLNLAKLNFNIIQATHLKDLKDVIRWWRDLDIVEDLFFTRDRIVECFYFAGGIGSKPQQG-SIRKWVTKVFQLVLIIDDVFDIYGSLAD
TPS27 222 E-KEEGVSKLKLLQLAKLNFNMVQAEHQKDLVHILRWWRNLGLIENVSFSRDRIVESFLWSVGVAFEPQHS-NFRNWLTKAITFIIVIDDVYDIYGTLQN
TPS5  279 ---MSN-ANPLLLELAKLDFNIVQATHQQDLRILSRWWKNTGLAEKLPFSRDILVENMFWAVGALFEPQHS-YFRRLITKVIVFISIIDDIYDVYGTLDE
TPS7  261 ---MPN-ANPLLLELAKLDFNIVQAAHQQDLKILSRWWKSMSLAEKLSFSRDRLVEDFFWSVGLAFEPQHS-LCRRMLAKNVAFIIVIDDIYDVYGSLDE
TPS4  259 ---MPN-ANPLLLELAKLDFNIVQATHQQDLKSLSRWWKNMCLAEKLSFSRNRLVENLFWAVGTNFEPQHS-YFRRLITKIIVFVGIIDDIYDVYGKLDE
TPS3  271 ---IPN-ANPLLIELAKLDFNFVQAMHQQELRNLSRWWKKSMLAEKLPFARDRIVEAFQWITGMIFESQENEFCRIMLTKVTAMATVIDDIYDVYGTLDE
TPS8  241 ---RQDKMIPFLLELATLDFNIVQAAHIEDLKYVARWWKETCLAENLPFARDRLVENFFWTIGVNFLPQYG-YFRRIATKVNALVTTIDDVYDVFGTLDE
TPS38 215 ---KEN-MNPLLLELAKLDYNMVQATYLEELKQMSRWDKNMKLVKKMSFVRDRLVEGFFWAVGFTPNPQFG-YCRKLSTKLSVLLTTIDDIYDVYGTLDE
TPS37 234 ---INGWGKNTLKELANMDYFITKEIHQHELAQVFRWWKSLGLAEELKLLRDQPLKWYTWPMAMLTDPKMS-QERIELAKCISFVYVIDDIFDVYGTIEE
TPS39 235 ---INGWGKSTLQELANMDYSITKEIHQHELIQVSRWWSSLGLAEDLKLLRDQPLKWYTWPMTMLTDPKMS-QQRIELAKCISFVYVVDDIFDVYGTIEE
                                                                                             DDxxD

TPS25 316 VQQFTVAIEKWDPEEVQRLPKSIQICFGALHDTMEDISVEIQRQKG--GPSVLPHLKQVWVNFCKALLVEATWYHKGHIPTLEDYLHNGWTSSSGPLLSL
TPS47 324 AQQFTHAINKWDPNEVKCLPECIQICFRALYDTVEEIYAEIDDQQKGCQHSAFPSLKQGWLNFCKAMLLEAKWYNEGHIPTLEEYLNNGWISSTVPLLSD
TPS27 320 LQLFTDAVVRWDPKVVEQLPSCMQICFWKLYDTTNDVALEIQQQKG-CKFPVLTYLQKVWAEFCKALLVEAKWDSKGYTPTFSEYLENGWKSSGGTVLSL
TPS5  374 LELFTLAIQRWDTKAMEQLPDYMKVCYLALINIINEVAYEVLKNHD---INVLPYLTKSWADLCKSYLQEAKWYHNGYKPNLEEYMDNARISIGVPMVLV
TPS7  356 LEIFTHAVERWDIKAMEQLPDYMKICYLSLFNTTNEMAYHILKQQG---INVLPYLTKQWTDLCKSYLQEAKWYHNGHKPRLEEYMDNAWISIATPLVLL
TPS4  354 LELFTLAVQRWDTKAMEDLPYYMQVCYLALINTTNDVAYEVLRKHN---INVLPYLTKSWTDLCKSYLQEARWYYNGYKPSLEEYMDNGWISIAVPMVLA
TPS3  367 LEIFTHAIQRMEIKAMDELPHYMKLCYLALFNTSSEIAYQVLKEQG---INIMPYLTKSWADLSKSYLQEARWYYSGYTPSLDEYMENAWISVGSLVMVV
TPS8  337 LQIFTHAIERWSIDELDRLPDNMKMCYYALDNFINQLADDAFEEQG---IFISPYLRNSWRDLCKSYLREAKWYHSQYIPSMEEYMDNAWISISAPVILV
TPS38 310 LELFTDIVDRWDINAIEQLPEYMKISFLALFNSMNELAYDILKEQG---FSIISHIRKQWANLCKAYLLEVKWYQRGYTPSLDEFLRNAWITNTGPVLIM
TPS37 330 LTLFTQAVHRWELSAMMDLPEYMRSLYKALYNTINSIGYNIYKIYG---QNPTQNLQNTWAHLCSAFLIEAKWFACGMVPTTDEYLKNGLVSSGVYVALI
TPS39 331 LTLFTQAVNRWELCVMKDLPEYMRATYKALYDTINSIGYNIYKIYG---QNPTQNLRNAWANLCNAFLKEAKWFASGELPTTDEYLKNGLVSSGVHVVLV
```

```
TPS25  414 HVILGLTN-----ENLHLCK-NCQEIIYYTSLIIRLCNDQGTSTVELERGDVASSIICYMH-QENVSEDVAREHIESIILNSWEKTN-YHFNRLSTSHRK
TPS47  424 YVIYGLTNNKITNESLDSSN-NFQEIIYHTSVIFRLCNDQGTSAAELERGDVASSIICYMQ-QENVSEDVAREHIESIILDSWKKIN-YHFNTLSMSHRE
TPS27  419 HVLLGLAQDFS--QVDYFLE-NERDLIYYSSLIIRLGNDLGTSTAELERGDVSSSILCYMR-KENVKEDVARKHIEEMVIETWKKMN-RHCFENSS---P
TPS5   471 HSLFLVTNQITKEALDSLTN--YPDIIRWSATIFRLNDDLGTSSDELKRGDVSKSIQCYMN-EKGASEEEAIEHIEFLIQETWEAMN-TAQSKNSPLSET
TPS7   453 HAFIFLTNPITQEALESLNN--YPDIIRRCAIINRFVDDLGTSSDELKRGDVPKSIQCYMN-DTGASEEEAREHINLLIKEMWEVMNKDQISKQVLFSEE
TPS4   451 HALFLVTDPITKEALESLTN--YPDIIRCSATIFRLNDDLGTSSDELKRGDVPKSIQCYMN-EKGVSEEEAREHIRFLIKETWKFMNTAHHKEKSLFCET
TPS3   464 NAFFLVTNPITKEVLEYLFSNKYPDIIRWPATIIRLTDDLATSSNEMKRGDVPKSIQCYMK-ENGASEEEARKHINLMIKETWKMINTAQHDN-SLFCEK
TPS8   434 HAYFLVANPVNKEALHYLEN-NYHDIIRCSALILRLANDLGTSSDELKRGDVPKSIQCYMN-ETQASEEEARQYIRLLISQTWKKLNEAHWLAADPFPKI
TPS38  407 HAYFCITNPIKEDELQRLN--HYPAIIYSPSLILRLANDLATSPDEIKKGDYLKSIQCYMH-DSKSCEENARNYIKKLIDETWKKMNR-DILRDESLSKD
TPS37  427 HLFYILGLGVS---SMHLQD--ISLMSTSIAKILRLWDDLGSAKDENQEGKDGSYVEYYMKENKDSSMELAREHVIKLIEDEWKQLNKEHFCLMSQSTRS
TPS39  428 HMFYLLGFGLNNQNSIYLED--SSAMASSVATILRLWDDLGSAKDENQEGNDGSYIECYMKGQKNASIELAREYVVKLIEDEWKQLNKKHFNLMNGSLGS

TPS25  506 IMKHVINEARMAHVMYLSGDGFGVQDGETQDQVLINLVQSII------------------------
TPS47  521 IAKHVINIARMGHVMYQFGDGFGVQDGKTRDQILINLMEPIT------------------------
TPS27  511 LIKYIMNIARVTHFIYQNGDGFGVQDRETRQQILSSLVQSLPLN----------------------
TPS5   567 FIEVAKNITKASHFMYLHSD--------VKSSISKILFEPIIISNVAFALK---------------
TPS7   550 FIKIVFNFSRTSHCVYQHGDGHGIQNSHITNRISKLLFEPLII-----------------------
TPS4   548 FVEIAKNIATTAHCMYLKGDSHGIQNTDVKNSISNILFHPIII-----------------------
TPS3   562 FMGCAVNIARTGQTIYQHGDGHGIQNYKIQNRISKLFFEPITISMP--------------------
TPS8   532 FVTCAMNLARMAQCMYQHGDGHGGNNSTTKNHIMALLFESVPLGHKHSSAEKEDHSMVNYREKFMI
TPS38  503 FRRTSMNLARIAQCMYQHGDGFGIPDRETKDRILSLFFQPIPLT----------------------
TPS37  522 FSKASLNSARMVSLMYSYDDKQSLPILQEYIKSMLDGNL---------------------------
TPS39  526 YSKASLNLARMVPLMYNYDDKQSLHVLQEYINTMLYDV----------------------------
```

Fig. S14 Sequence alignment of the proteins encoded by the functional tomato TPS-e/f genes. Black shading indicates identical amino acid residues and grey shading are the conserved or semi-conserved substitutions observed and dashes indicate gaps. The truncation sites for protein expression are indicated with triangle.

```
                                                                                                           ▼
TPS19    1 ------------------MIVGYRSTIITLSHPKLGNGKTISSNAIFQRSCRVRCSHSTTSSMNGFEDARDRIRESFGKLELSPSSYDTAWVAMVPS---
TPS20    1 ------------------MIVGYRSTIITLSHPKLGNGKTISSNAIFQRSCRVRCSHSTTSSMNGFEDARDRIRESFGKLELSPSSYDTAWVAMVPS---
TPS21    1 ------------------MLIGCRSKIIIISHHKLGNGKTISSNAIFQRSCRARCSHSTTSSMNGFEDARDRIRESFGKLELSPSSYDTAWVAMVPS---
TPS18    1 ------------------MIIGYRINFRPLSHDKLRSHVMWQR----------QCSYNTASSMDGFEEAKERIRESFSKVELSPSSYDTAWVAMVPS---
TPS24    1 MSATIIFPAASSSSSYLSVVKHQMIRDITIPSRRLGGGLSFTQHSSSTAACVVDATRGPDFALQCNETTKERIRKLFHKVEFSVSSYDTAWVAMVPS---
TPS46    1 -------------------------------------------------MDSSPLSSIESLVHDIKKDLFSNQENFNTFLTKTTFAYDIAWLAMIPFDNQ

TPS19   80 RHSLNEPCFPQCLDWIIENQREDGSWG-LNPTHPLLLKDSLSSTLACLLALTKWRVGDEQIKRGLGFIETYGWAVDNKDQ--ISPLGFEVIFSSMIKSAE
TPS20   80 RHSLNEPCFPQCLDWIIENQREDGSWG-LNPTHPLLLKDSLSSTLACLLALTKWRVGDEQIKRGLGFIETYGWAVDNKDQ--ISPLGFEVIFSSMIKSAE
TPS21   80 RHSLNEPCFPQCLDWIIENQREDGSWG-LNPTHPLLLKDSLSSTLACLLALTKWRVGDEQIKRGLGFIETYGCAVDNKDQ--ISPLGFEVIFSSMIKFAE
TPS18   70 KYSLNEPCFPQCLDWIIENQREDGSWG-LNPTHPLLLKDSLSSTLACLLALTKWRVGDEQIKRGLGFIETQSWAIDNKDQ--ISPLGFEIIFPSMIKSAE
TPS24   98 PHSAKVPCFPECLHWVLHNQLEDGSWG-LPHHQPLLLKDVLSSTLACVLALKRWGIGEQLISNGLRFIELNFASATDEDQ--YSPIGFDVIFPGMLEYAQ
TPS46   52 KKEANGPMFVSCLNWILNNQNEQGFWGESNGENQIPTINTLPITLACMVALKKWNVGERHIKKGLKFVHANMYTLLKENSQCFPCNWFTIVFPSILQLAK

TPS19  177 KLDLNLPLNLHLVNLVKCKRDSTIKRNVE-----------YMGEGVGELCDWKEMIKLHQRQNGSLFDSPATTAALIYHQHDQKCYQYLNSIFQQHKNW
TPS20  177 KLDLNLPLNLHLVNLVKCKRDSTIKRNVE-----------YMGEGVGELCDWKEMIKLHQRQNGSLFDSPATTAALIYHQHDQKCYQYLNSIFQQHKNW
TPS21  177 KLNLNLPLNLHLVNLVNCKKDSTIKRNDE-----------YMGEGVGELCDWKEIIKLHQRQNGSLFDSPATTAALIYHQRDQKCYEYLNSILQQHKNW
TPS18  167 KLNLNLAMNKIDSTIKRALQN-EFTRNIE-----------YMGEGVGELCDWKEIIKLHQRQNGSLFDSPATTAALIYHQHDQKCYEYINSILQQHKNW
TPS24  195 HLSLKLHLESGVFNELLHKRAIQLTRPYDSSSLELNAYLAYVSEGIGELQDWKMVMKYQ-RKNGSLFNSPSTTAASLI-HLHDSGCLDYLRGALKKFGNA
TPS46  152 AQGLEIIIVNGSN-----------------------------------KLLSDVIMKKKALLESENLNDNIVSEAQAFMSTGNKKCLQYLMSIVQQCPNG

TPS19  266 VPTMYPTKVHS-LLCLVDTLQNLGVHRHFKSEIKKALDEIYRLWQQK-----------------NEQIFSNVTHCAMAFRLLRMSYYDVSSDELAEFVDE
TPS20  266 VPTMYPTKVHS-LLCLVDTLQNLGVHRHFKSEIKKALDEIYRLWQQK-----------------NEQIFSNVTHCAMAFRLLRMSYYDVSSDELAEFVDE
TPS21  266 VPTMYPTMIHS-LLCLVDTLQNLGVHRHFKSEIKKALDEIYRLWQQK-----------------NEEIFSNITHCAMAFRLLRMSNYDVSSDELAEFMDE
TPS18  255 VPTMYPTKIHS-LLCLVDTLQNLGVHRHFKSEIKKALEEIYRLWQQK-----------------NEEIFSNVTHCAMAFRLLRMSYYNVSSDELAEFVDE
TPS24  293 VPTIYPINIHA-SLCMVDDLKKLGICRHFSEEIQNVLDETYRCWLQG-----------------EDEIFTSAGTCSMAFRILRGYGYNVSSDPVAQFLEQ
TPS46  217 VSSRFPVDEELKMLCMVDNIQRMGLAKHFEKEIEQILDQVYNNYQINNLKNNNEYLLSSEEEKTSDDLPDKLLKDSLVFRLLRLQGYKINQGSFCWFLQP

TPS19  348 EHFFATNGKYTSHVEILELHKASQLAIDHEKDDILDKINN----WTRAFMEQKLLNNGFIDRMSKKEVELALRKFYTTSHLAENRRYIKSYEENNFKILK
TPS20  348 EHFFATNGKYKSHVEILELHKASQLAIDHEKDDILDKINN----WTRAFMEQKLLNNGFIDRMSKKEVELALRKFYTTSHLAENRRYIKSYEENNFKILK
TPS21  348 EHFFTTSGKYTSHVEILELHKASQLAIDQEKDDILDKINN----WTRTFMEQKLLNNGFIDRMSKKEVELALRKFYTTYDRAENRRYIKSYEENNFKILK
TPS18  337 EHFFSTSGKFISDVAIIELHKASQLTIN-EKDDILDKINN----WTGIFMQQKLLNNDFLDIKSKKEVELALRMFYVTYDRAENRRYIESYQENNFKMLK
TPS24  375 EQYS---GHLNDIHTMLDLYQALEMIIATDKPVSMKLNSSSLQSLIQRLSDEFYPPNGLTKQIREQVDDVLKFPSHANIKRVANRRNIKHYDVDNTRVLK
TPS46  317 -HLKDKIEKNKEQFTYVMYNVYRATDLMFNGESQMEEARF----FARNFLMNNDNYLSLFPSLQKVIEHELNVPWFARLEHLDHRLWIELNQSIPLSIGK

TPS19  444 AAYRSPDIN-NKDLLAFSIHDYELCQAQHREELQQLKRWFEDYRLDQLGLGERYIHASYLFGVTIIP--EPELSDARLMNAKYAMLLTIVDDHFESFASK
TPS20  444 AAYRSPNIN-NKDLLAFSIHDFELCQAQHREELQQLKRWFEDYRLDQLGLAERYIHASYLFGVTVIP--EPELSDARLMYAKYVMLLTIVDDHFESFASK
TPS21  444 AAYRSPNIN-NKDLLIFSIHDFDLCQTQHREELQQLKRWFQDCRLDQLGLSEQFISTTYLIGIAVVS--EPEFSNARLMYAKYVMLLTIVDDLFDGFASK
TPS18  432 TAYRCGSMN-NIDLLTFSMQEFELGLSQYQEEVEQLKRWYEDYRLEQVGLAQEYIYRTHLISVAVFF--EHELSNARIMYAKYAMFLTLSDDLFEHLASK
TPS24  472 TSYSSSNFG-NKDFLTLAVEDFNLCQSIHRNELKQLERWLTQNRLDKLKFVRERSAYCYFSAAATIF--QPELSDARMSWAKNGVLTTVIDDFFDVGGSM
TPS46  412 SSFYWLSYLQNEKLMQLAVQNYEYRQSIYRKELEDLKRWSKEKGLVDIGFGREKTTYSYFASAASSSSFLPYESFLRLIVAKCSIIITVADDFYDEEASL

TPS19  541 DECLNIIELVERWDDYASVGYKSEKVKIFFSTFYKSIEELATIAEIKQGRSVKNHLINLWLEMMKLMLMEQVEWCSGKTIPSIEEYLYVTSITFCAKLIP
TPS20  541 DECFNIIELVERWDDYASVGYKSEKVKVFFSVFYKSIEELATIAEIKQGRSVKNHLINLWLELMKLMLMERVEWCSGKTIPSIEEYLYVTSITFCAKLIP
TPS21  541 DELLNIIQLVERWDDYASVGYNSERVKVFFSVFYKSIEELATIAEIKQGRSVKNHLINLWLEVMKMMLIERIEWWTSKTIPSIEEYLYVTSITFGSRLIP
TPS18  529 DELLNIIELVQRWDEHTNVGFHSEKVKLFFTALYDTIEEVATNAQIKQGRNVKHHIIELFVEGLNSMLVDRVEWG--TRIPSIEEYLRVSLSTFGGKCMV
TPS24  569 EELNNLILLFKKWDVDVSTDCCSERVGIIFSALHSTISEIGDKASKWQARSVTRHITDIWLNLLNAMLRE-AEWAKDMSVPSLDKYMANGYVSFALGPIF
TPS46  512 SELHILSEAIQRWDAKNLDGPS----KIIFEALDDLVCDVAKMYHLQHKIDITPQLRHMWKETFDAWMME-SLWSRTSNLPSRDKYLQVGMISVGAHILV

TPS19  641 LTTQYFLGIKIS--KDLLESDEICGLWNCSGRVMRILNDLQDSKKEQKEGSITLVTLLMKS--------MSEEEAVMKTKEILEMNRRELLKMVLVQK-K
TPS20  641 LSTQYFLGIKIS--KDLLESDEICGLWNCSGRVMRILNDLQDSKREQKEVSINLVTLLMKS--------MSEEEAIMKIKEILEMNRRELLKMVLVQK-K
TPS21  641 LTTQYFLGIKIS--KDLLESDEIYGLCNCTGIVMRLLNDLQTYKREQGESSMNLVTILMTQSPR--RTNICEEEAIMKIKEILEMNRRELLKMVLVQK-K
TPS18  627 LTSQYVVGIHLC--NYQSD-DEIQDLCYCSGIVMRLLNDLQSFKRERSDSRLVNMVKLVMKQRSGTICEEEEEEAIKHIKETIECNRRKLLRMVLQSKGK
TPS24  668 LPALYFVGPKLP--DDVVQHPEYHSLFELVSTCGRLLNDIRSFERESKDGKLNAVTLSVTHGNG----RISEEAAIEGLSHRVEMQRKELLKLVLQRE--
TPS46  607 LHAASLGCTILPNQVFGPINGQYENITKLLMTTTRLLNDIQSYQKEREVGKMNYTLLHLNENPR-----GQIEDSIDFVKVILGDKKKEFLENVLMDG--

TPS19  730 GSQLPQLCKDIFWRSSKWAHFTYSQTDGYRIPEEMKNHIDEVFYKPLNH---------------------------------------------------
TPS20  730 GSQLPQLCKDIFWRTSKWAHFTYSQTDGYRIAEEMKNHIDEVFYKPLNH---------------------------------------------------
TPS21  736 GSQLPQLCKDIFWRTSKMVYFTYSHGDEYRFPEEMKNHIDEVIYKPLNH---------------------------------------------------
TPS18  724 GSKVPQALKDLFWRTTKAVYFFYSDHDEFRSPNKVKHHINQVIYKPLHNR--------------------------------------------------
TPS24  760 GSVVPNACKDLFWEMSKVLHQFYIKDDGFSSMG-MADTVNAIIHEPITLNYLGDSKLITDYN--------------------------------------
TPS46  700 FNDMPRTMKLLHLSCLNVFHMFFNSCNLFDTKSAILEDIMRAIYIPLGQNEQTKVMAIQEKKIKKKIEIVKVNACVNYEINMKQLQVGVGISYVKRQNPK

TPS19      ----------------------
TPS20      ----------------------
TPS21      ----------------------
TPS18      ----------------------
TPS24      ----------------------
TPS46  800 NYNSFGLSTKGFASHNLRLCFM
```

Fig. S15 Sequence analysis of TPT homologs from tomato and other plants. Black shading indicates identical amino acid residues and grey shading are the conserved or semi-conserved substitutions observed and dashes indicate gaps. The two aspartate-rich motifs (FARM, first aspartate-rich motif; SARM, second aspartate-rich motif) are underlined, the black arrow indicates the site of truncation of the N-termini for protein expression in this study. AtGGPPS11, geranylgeranyl diphosphate synthase 11 from *Arabidopsis thaliana* (At4g36810); AtGFPPS1, geranylfarnesyl diphosphate synthase 1 (At1g49530); OsGGPPS1, geranylgeranyl diphosphate synthase 1 from *Oryza sativa* (Os07g39270); OsGPPS, geranyl diphosphate synthase (Os01g14630); AtSSU, small subunit of geranyl(geranyl) diphosphate synthase (At4g38460); OsGRP, GGPPS recruiting protein (Os02g44780); AmSSU, small subunit of geranyl diphosphate synthase from *Antirrhinum majus* (AAS82859).

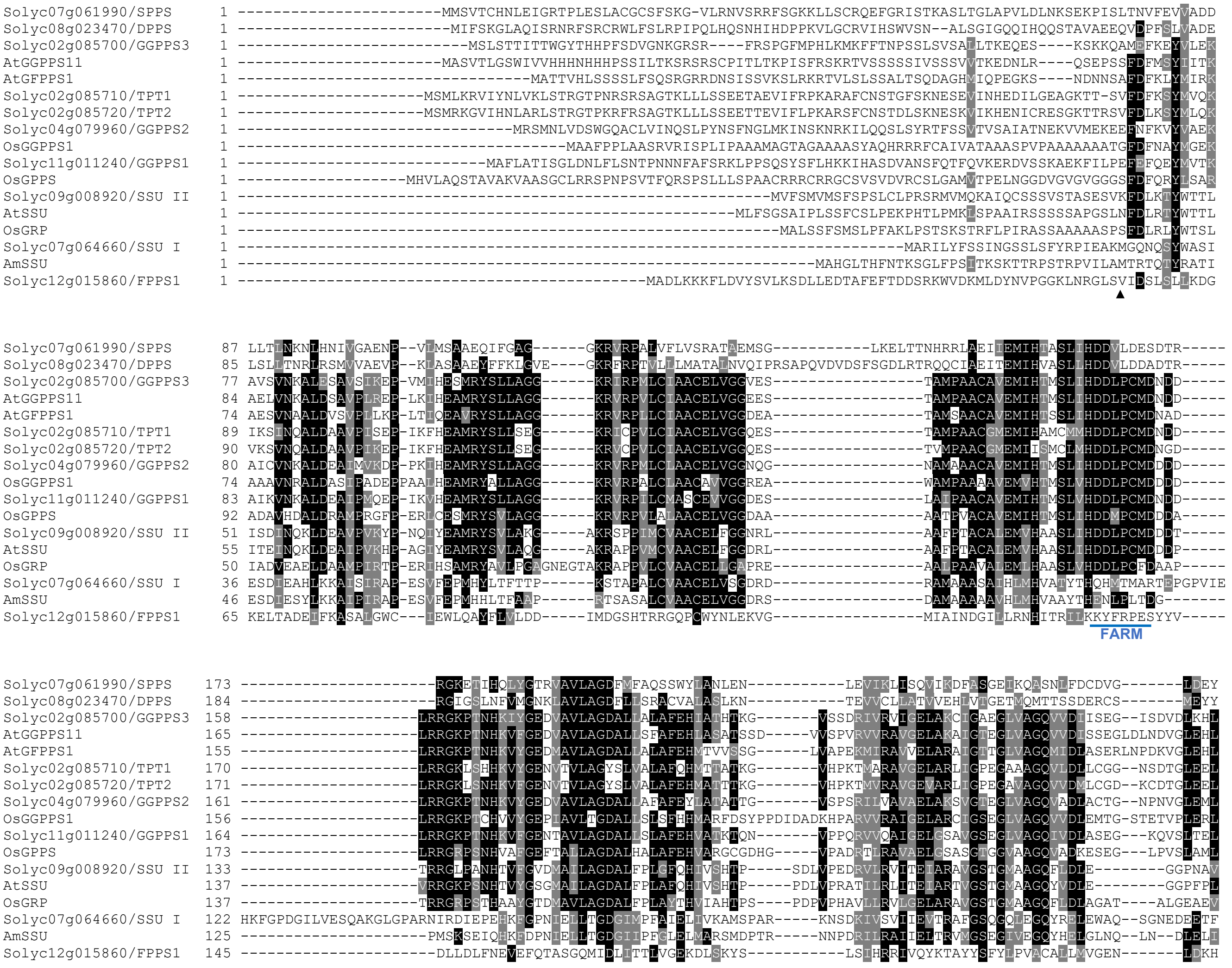

```
Solyc07g061990/SPPS    243 LLKSYYKTASLIAASTKGAAIFSEVGSDISEQMFQYGRNLGLSFQIVDDILDFTQSAAQLGKPAGSDLAKGNLTAPVLFALEKEPNLRNIIESEFHDAGSLEEAINLVKS
Solyc08g023470/DPPS    254 MQKTYYKTASLISNSCKAIALLAGHSAEVSVLAFDYGKNLGLAFQLIDDVLDFTGTSATLGKGSLSDIRHGIVTAPILYAMEEFPQLRTLVDRGFDDPVNVEIALDYLGK
Solyc02g085700/GGPPS3 239 EFIHLHKTAALLEGSVVLGAILGGAPDEDVEKLRKFARCIGLLFQVVDDILDVTKSSQQLGKTAGKDLVADKVTYPKLIGIE----------------------------
AtGGPPS11              250 EFIHLHKTAALLEASAVLGAIVGGGSDDEIERLRKFARCIGLLFQVVDDILDVTKSSKELGKTAGKDLIADKLTYPKIMGLE----------------------------
AtGFPPS1               239 EFIHLHKTAALLEAAAVLGVIMGGGTEQEIEKLRKYARCIGLLFQVVDDILDVTKSTEELGKTAGKDVMAGKLTYPRLIGLE----------------------------
Solyc02g085710/TPT1    251 EYIHRHKTADFAEAAAVVGAMIGGASEKEINRLEKFSKCLGLLFQVVDDILDVTKSSEQLGKTAGKDLLANKLTYPKMIGID----------------------------
Solyc02g085720/TPT2    252 KYIHSHKTADFTEAAAIVGALLGGASEEEINRVRKFSQCFGLMYQVVDDILDVTKSSEQLGKTAGNDLLANKLTYPKMIGID----------------------------
Solyc04g079960/GGPPS2 242 EFIHIHKTAALLEASVVIGAILGGGADEEVDKLRRFAQCIGLLFQVVDDILDVTKSSSELGKTAGKDLAVDKTTYPKLLGLE----------------------------
OsGGPPS1               245 EYIHLHKTAALLEASVVIGAILGGGSDEQIESLRMYARSIGLLFQVVDDILDVTKSSEELGKTAGKDLASDKTTYPKLLGLE----------------------------
Solyc11g011240/GGPPS1 244 EYIHHHKTAKLLEAAVVCGAIMGGGNEVDVERMRSYARCIGLLFQVVDDILDVTKSSDELGKTAGKDLITDKATYPKLMGLE----------------------------
OsGPPS                 255 EYIHVHKTARLLEAAAVSGAIVGGGADAEVERVRRYARCVGLLFQVVDDVLDMTSTSEQLGKTAGKDVEADKATYPKLLGVD----------------------------
Solyc09g008920/SSU II  210 DFVQEKKYGEMGECSAVCGALLAGASDEEIQHMRKYGRAVGVLYRVVDDILEAKKTE-----NKTEGKKKKGKSYVSVYGIE----------------------------
AtSSU                  214 SFVQEKKFGAMGECSAVCGGLLGGATEDELQSLRRYGRAVGMLYQVVDDITEDKKKS-----YDGGAEKG----------------------------------------
OsGRP                  219 MKVLTKKFGEMAECSAACGAMLGGAGPDEEAALRRYGRTIGVLYQLVDDIRSAS----------GNGKMRSNASVLRALGMD----------------------------
Solyc07g064660/SSU I   225 EYVCKKKEGEIHACGATCGAILGDGNDEEIERLRNYGLYVGIIQGIIN----------------------------GRNYNK----------------------------
AmSSU                  205 EYVCKKKEGTLHACGAACGAILGGCDEDKIEKLRRFGLYVGTVQGLLG----------------------------KNRS------------------------------
Solyc12g015860/FPPS1   215 VDVKKILIDMGIYFQVQDDYLDCFADPEVLGKIGTDIQDFKCSWLVVKALELCNEEQKKILFENYGKDNAACIAKIKALYND----------------------------
                                                                            SARM

Solyc07g061990/SPPS    353 CGGIQRAQDLAKEKADLAMQNLKCLPSSP------FQAALEEIVKYNLERIE---------
Solyc08g023470/DPPS    364 SRGIQRTRELARKHASLASAAIDSLPESDDEEVQRSRRALVELTHRVITRTK---------
Solyc02g085700/GGPPS3 321 -----KSREFAEELNKEAKAQLVGFDQEKA-------APLFALANYIAYREN---------
AtGGPPS11              332 -----KSREFAEKLNREARDQLLGFDSDKV-------APLLALANYIAYRQN---------
AtGFPPS1               321 -----GSREVAEKLRREAEEQLLGFDPSKA-------APLVALASYIACRHN---------
Solyc02g085710/TPT1    353 -----KSKEYAQKLNKEAKEQLVGFDPEKS-------APLLAMADFVLHRQK---------
Solyc02g085720/TPT2    334 -----KSKEYAQKLSKEAKEQLVGFAPEKA-------APLLAMTDFLLHRQK---------
Solyc04g079960/GGPPS2 324 -----KAKEFAAELNGEAKQQLAAFDSHKA-------APLIALADYIANRQN---------
OsGGPPS1               327 -----KSREFAEKLLSDAREQLSGFDQETA-------APLLHLANYIAYRQN---------
Solyc11g011240/GGPPS1 326 -----KARQYAGELMAKAMNELSYFDYAKA-------APLYHIASYIANRQN---------
OsGPPS                 337 -----KAREYAADLLAMAEAELDGFDAERA-------APLRHLARFIAYRQH---------
Solyc09g008920/SSU II  287 -----KAVKVAEDLRAQAKRELDGLEKYG-----DKVMPLYSFLDYAADRGFSIDGQV---
AtSSU                  279 ------MMEMAEELKEKAKKELQVFDNKYGGG--DTLVPLYTFVDYAAHRHFLLPL-----
OsGRP                  291 -----RALGIVEELKAQAKMEADRFGDKYG----ERVLPLYSFVDYAVERGFELQDAATTP
Solyc07g064660/SSU I   279 -----KMEKRVNELRILAMKELNTSFKGKK--------LMKQIASLVEES-----------
AmSSU                  257 -----GFEGRIKELKELAVKELESFGGEKI--------ELIRGVFELEHSLAGV-------
Solyc12g015860/FPPS1   297 ----LKLEEVFLEYEKTSYEKLTTSIAAHP--SKAVQAVLLSFLGKIYKRQK---------
```

Fig. S16 GC-MS analysis of terpenes extracted from tomato leaves. No significant differences between terpenes were observed from three extraction methods, data present in Fig. 10 was obtained by using MTBE dipping method as described in Methods S1. Terpene signals are identified by comparison of their mass spectra and retention indices with authentic standards and NIST libraries. Monoterpenes and sesquiterpenes are indicated with red and blue dotted lines, respectively. Non-terpene compounds are indicated with asterisks. Terpenes identified from left to right are as follows: α-thujene, α-pinene, unknown 5, 1,3,5-cycloheptatriene, 3,7,7-trimethyl-, β-pinene, isolimonene, β–myrcene, (+)-2-carene, α-phellandrene, α-terpinene, m-cymene, β-phellandrene, β-ocimene, α-ocimene, γ-terpinene, terpinolene, unknown 6, *trans*-p-mentha-2,8-dienol, α-terpineol, nerol, geraniol, δ-elemene (germacrene C), β-elemene (germacrene A), β-caryophyllene, α-humulene, germacrene D, cis-muurola-3,5-diene, α-farnesene, E-nerolidol. Mass spectra and retention indices of the terpene compounds can be found in Fig. S3 and Table S2, respectively.

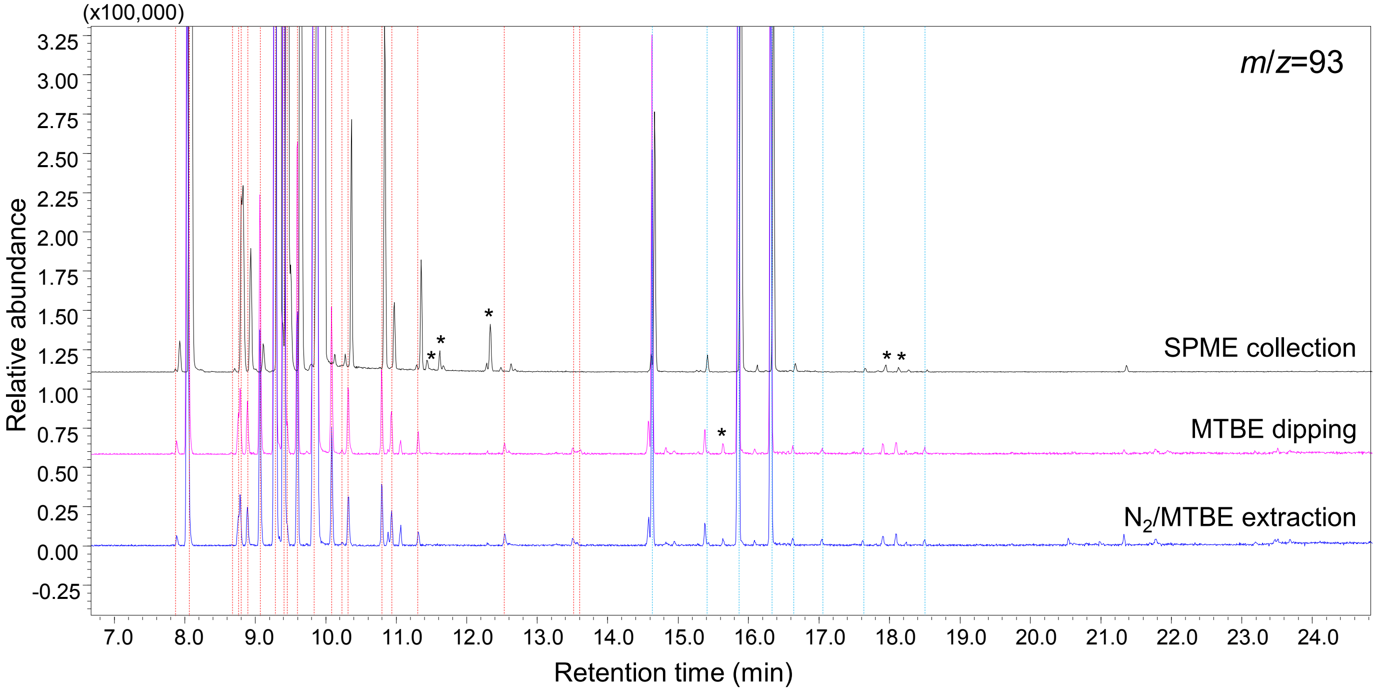

Fig. S17 Correlation analysis of terpene biosynthesis genes and volatile terpenes from tomato. The Spearman correlation coefficients were calculated using gene expression data and terpene profiling data as indicated in Fig. 9 and Fig. 10, respectively. Genes characterized in this work are shown in bold. The hierarchical clustering was performed using average linkage method by Cluster 3.0.

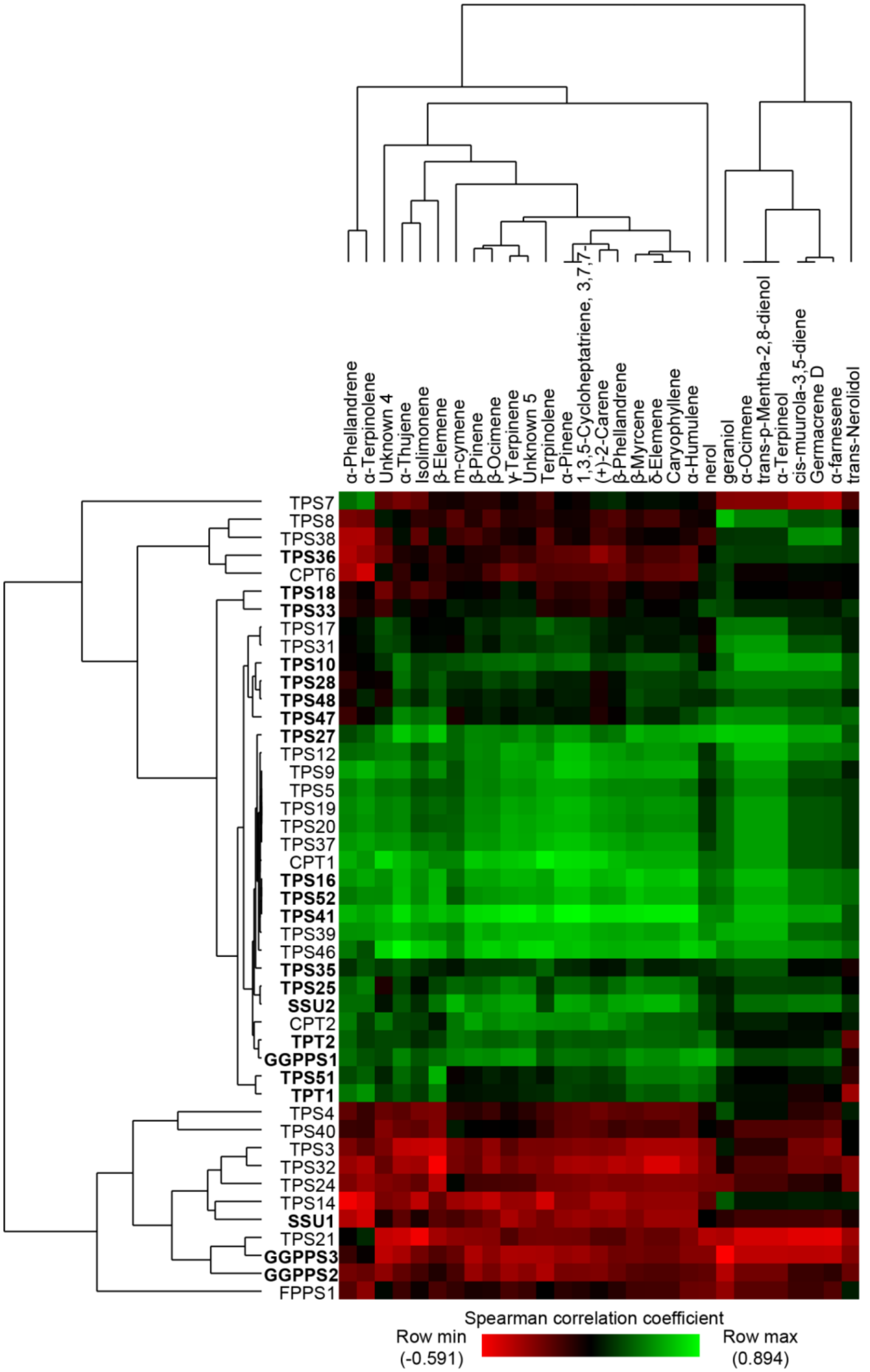

Fig. S18 Terpene biosynthesis gene clusters identified from tomato genome by plantiSMASH. The functional TPS genes are shown in bold. The eight clusters (7, 10, 17, 18, 26, 36, 42 and 43; numbered in the analysis of plant secondary metabolite clusters for *Solanum lycopersicum*) are obtained from plantiSMASH website (http://plantismash.secondarymetabolites.org/precalc/precalc/Solanum_lycopersicum/).

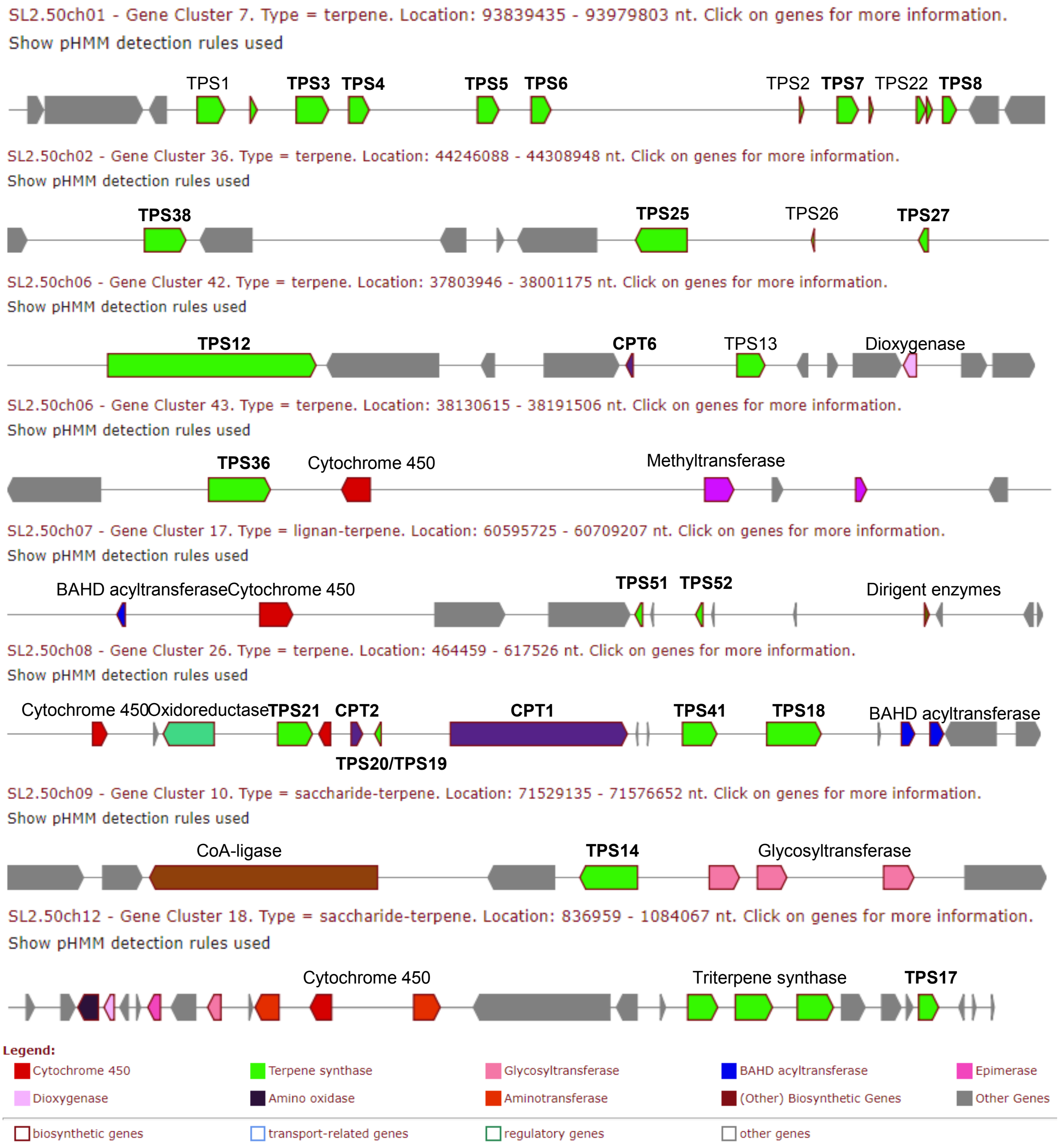

Table S1 Primers used in this work.

Primer name[1]	Sequence (5'-3')[2]
For cloning of genomic DNA and sequencing	
TPS47-F	CACACTTGATGTACCTTCCAATGTAC
TPS47-R	TTATGTAATAGGCTCCATCAAGTTGATTAGG
TPS48-F	GTATTATCACAATATAGCAACAATGAC
TPS48-R	TTACAGCTTGATGGAGTTGACGAGTAAAGC
TPS49-F	ATGGAATACTTAAACAAATATCC
TPS49-R	TAATTGAGACAATATGCATTGAG
TPS50-F	ATGGAGATTAACAATATGAATGTTC
TPS50-R	GTCACATTCATACATAAATACATAATG
TPS51-F	TGTTGGTTCTTTTCTTTCACC
TPS51-R	TTCATCATTCAGTAATAATTCTTTG
TPS52-F	ATGGTCGTTGGATTTGGTGGTGAAGC
TPS52-R	CAAGCTTTTAATCTTGACTATATTTTC
TPS53-F	CTACTTGGGTTCAATTCTAAGTAATTATTACAC
TPS53-R	CTCAAACTCTTAATCATTACTCTACCTC
For cloning in pET32b and creating His-tagged recombinant protein	
TPS10-opti-NdeIF	GCTAATCCATATGGCATCCTCCAGTAGTACG
TPS10-opti-XhoIR−	CCGCTCGAGACTGATGGAAATTTTTACGCTC
TPS16-opti-NdeIF	GCTAATCCATATGCGCCGCGTGGGGTCCCACCAC
TPS16-opti-XhoIR−	CCGCTCGAGGATAATTGGGTCGATAAGGATACG
TPS25-NdeIF	GCTAATCCATATGGAAAGGATTTCTACCTATAAACCAAAC
TPS25-NotIR−	TTCACGCGGCCGCTATAATAGACTGTACCAAGTTTATAAG
TPS27-NdeIF	GCTAATCCATATGCGACGAAATGCTAATTACAAGCC
TPS27-XhoIR−	CCGCTCGAGATTGAGAGGAAGTGATTGAACC
TPS47-opti-NdeIF	GCTAATCCATATGACTACGATTTCATCCTATAAGCCC
TPS47-opti-XhoIR−	CCGCTCGAGAGTAATTGGCTCCATAAGATTAATTAAAATC
TPS51-opti-NdeIF	GCTAATCCATATGGACGGCTTCGGGGATGAGG
TPS51-opti-XhoIR−	CCGCTCGAGGGTTGTGATAGGATCGATTAAG
TPS52-opti-NdeIF	GCTAATCCATATGGTGGTAGGATTTGGCGGCG
TPS52-opti-XhoIR−	CCGCTCGAGTGCCTTGATCGGGTCGATAAGTACC
dGGPPS1-NdeIF	GCTAATCCATATGCCTGAGTTTGAGTTTCAAGAATAC
GGPPS1-XhoIR−	CCGCTCGAGATTCTGTCGATTTGCAATATAAC
dGGPPS2-NdeIF	GCTAATCCATATGGAAAAAGAAGAATTTAATTTC
GGPPS2-XhoIR−	CCGCTCGAGATTTTGACGATTAGCAATGTAATC
dGGPPS3-NdeIF	GCTAATCCATATGGAGTTTAAAGAATACGTTCTTG
GGPPS3-XhoIR−	CCGCTCGAGATTCTCTCTGTAAGCAATATAATTTGC
dTPT1-XbaIF	GCTCTAGATGAGTGTCTTTGATTTCAAAAGTTAC
TPT1-EcoRIR−	CGGAATTCCATTTTTGCCGATGAAGAACG

dTPT2-XbaIF	GCTCTAGATGAGTGTCTTTGATTTGAAAAGTTAC
TPT2-EcoRIR−	CGGAATTCCATTTTTGCCGATGAAGAAG
To create non-tagged protein in pET32b and co-express with pET28a-SSU I/SSU II	
GGPPS1-XhoIR+ (with dGGPPS1-NdeIF)	CCGCTCGAGTCAATTCTGTCGATTTGCAATATAAC
GGPPS2-XhoIR+ (with dGGPPS2-NdeIF)	CCGCTCGAGTTAATTTTGACGATTAGCAATGTAATC
GGPPS3-XhoIR+ (with dGGPPS3-NdeIF)	CCGCTCGAGTTAATTCTCTCTGTAAGCAATATAATTTGC
For cloning in pET28a and creating His-tagged recombinant protein	
TPS28-NcoIF	CATGCCATGGTTCGCCCAGAGGCTAATTTCTCTC
TPS28-XhoIR−	CCGCTCGAGCATATTGATAGGATTCACGAGTAAAG
TPS33-NcoIF	CATGCCATGGTTCGCCCTGTTGCTGATTTCTCTCC
TPS33-XhoIR−	CCGCTCGAGAATCTCAACAGAATCTACCAACAAGGC
TPS35-NcoIF	CATGCCATGGGCCCTGTTGCTGACTTCTCTCC
TPS35-XhoIR−	CCGCTCGAGAATTTCAATAGAGTCCACCAACAACGC
TPS36-NheIF	TTCACGCTAGCATGGATGTTAACTCTTCACGTC
TPS36-NotIR−	TTCACGCGGCCGCTTATGATCGTCCTATAGTAAC
TPS48-NcoIF	CATGCCATGGACCGTCTAGAGGCTAATTTTTCTCCTAG
TPS48-XhoIR−	CCGCTCGAGCAGCTTGATGGAGTTGACGAGTAAAG
dSSUI-NdeIF	GCTAATCCATATGGGCCAAAATCAATCTTATTG
SSUI-EcoRIR−	CGGAATTCAGACTCTTCAACAAGACTAGC
dSSUII-NdeIF	GCTAATCCATATGACAGCATCTGAGTCCGTCAAG
SSUII-EcoRIR−	CGGAATTCGACTTGGCCATCAATGCTAAAAC
For cloning in pEZS-NL and subcellular localization analysis	
TPS3-XhoIF	CCGCTCGAGATGTCAATATTCTCCACAAGATATTTAG
TPS3-BamHIR−	CGGGATCCCGACGCCTAATAGGATTAATTATTCCATTTG
TPS4-EcoRIF	CGGAATTCATGAAGGCAATATTATTGAACAACATAGG
TPS4-BamHIR−	CGGGATCCCGCATAGTAGGTTTGTAATTTCCTGAACG
TPS5-XhoIF	CCGCTCGAGATGGTTTCAATATTGAGTAACATAGGG
TPS5-BamHIR−	CGGGATCCCGAGGCTTGTAATTCCCTGAACGCCTTG
TPS7-EcoRIF	CGGAATTCATGGTTTCAATATTCAGTAACGCAGGG
TPS7-BamHIR−	CGGGATCCCGAGGCTTGTAATTCCCCGAACGCCTTG
TPS8-XhoIF	CCGCTCGAGATGTATAAACTGGAAATGACAATGTC
TPS8-BamHIR−	CGGGATCCCG TTTCTCGTCTGAATAATGATTCTTCAGTG
TPS9-XhoIF	CCGCTCGAGATGGCTGCTTCTTCTGCTGATAAGTGTC
TPS9-BamHIR−	CGGGATCCCGCAA AAA TGT TTT GAA TGG ATG TTT C
TPS10-EcoRIF	CGGAATTCATGGCTTCTTCTTCTTCTACTAATAAG
TPS10-BamHIR−	CGGGATCCCGCGCGTCGATCAACACAAGCTTTTG
TPS12-XhoIF	CCGCTCGAGATGGCTTCTTCTTCTGCTAATAAGTGTCG

TPS12-BamHIR−	CGGGATCCCGCAAAAATGTTTTGAATGGATGTTTC
TPS16-EcoRIF	CGGAATTCATGGAGCTGTGCACACAAACCGTTC
TPS16-BamHIR−	CGGGATCCCGGTAACTCTCAATCTCATGTTCAAAATG
TPS17-XhoIF	CCGCTCGAGATGGAGTTGTGCACACAAACCGTTGC
TPS17-BamHIR−	CGGGATCCCGGATTCATCAATCTCACTTTGAAAATG
TPS18-EcoRIF	CGGAATTCATGATAATTGGCTATAGAATCAATTTC
TPS18-BamHIR−	CGGGATCCAAGGTAGGGTTTAGTCCCCAAGATC
TPS19-XhoIF	CCGCTCGAGATGATAGTTGGCTATAGAAGCACAATC
TPS19-BamHIR−	CGGGATCCCGAGAAAGTGAGTCCTTTAGAAGCAATGG
TPS20-XhoIF	CCGCTCGAGATGATAGTTGGCTATAGAAGCACAATC
TPS20-BamHIR−	CGGGATCCCGAGAAAGTGAGTCCTTTAGAAGCAATGG
TPS21-XhoIF	CCGCTCGAGATGCTAATTGGCTGTAGAAGCAAAATC
TPS21-BamHIR−	CGGGATCCCGAGAAAGTGAGTCCTTTAGAAGCAATGG
TPS24-XhoIF	CCGCTCGAGATGTCTGCTACCATTATTTTTCCTG
TPS24-BamHIR−	CGGGATCCCGATCTTCAAGTTGATTATGCAACACCC
TPS25-EcoRIF	CGGAATTCATGGCTTGCATAAACATGGTGTC
TPS25-BamHIR−	CGGGATCCTTTGTATCAACCTCAAAGTAAGCAC
TPS27-EcoRIF	CGGAATTCATGACAAGTGAGCAGCAATCTG
TPS27-BamHIR−	CGGGATCCCGTAGAAACACCATGATTTCCTTGTC
TPS28-EcoRIF	CGGAATTCATGAGTTTATTGGAAGGAAATGTAAACC
TPS28-BamHIR−	CGGGATCCCGCTTGCTTAGTTGATCTTCAATCTCC
TPS31-XhoIF	CCGCTCGAGATGGCCCCAGCTGCTGCATTGATGAG
TPS31-BamHIR−	CGGGATCCCGTCAACATGTCGTCTATTTGTTTTTC
TPS32-XhoIF	CCGCTCGAGATGGCCCTACTTAACAACCAAGATGAG
TPS32-BamHIR−	CGGGATCCCGCCAACATGTCCTCTATTTGTTTTTC
TPS33-EcoRIF	CGGAATTCATGGCCTCAGCTGCTGCATTGATG
TPS33-BamHIR−	CGGGATCCCGGTCCTCTATTTGTGTCTCAAAATG
TPS35-EcoRIF	CGGAATTCATGGCCTCAGCTGCTGCATTGGTG
TPS35-BamHIR−	CGGGATCCCGTTTGTAAATTTGATCCAACACATCG
TPS37-XhoIF	CCGCTCGAGATGGCAAATATTACTAAAGCTTTCTCCC
TPS37-BamHIR−	CGGGATCCCG GCGATTATAGCTCGTTTGAAGATAAGAGG
TPS38-XhoIF	CCGCTCGAGATGCTTCAAAGTTGCATATCAACTATG
TPS38-BamHIR−	CGGGATCCCGAATTCTAAAGCTGTATCATACAACTC
TPS39-XhoIF	CCGCTCGAGATGGAAATGACTAAAGTCTTGATTTCCC
TPS39-BamHIR−	CGGGATCCCGGTACGTGGAGGTTGCTCTGAGAAGATC
TPS40-XhoIF	CCGCTCGAGATGTCGATCTCAGCTTCTTTTTTAAG
TPS40-BamHIR−	CGGGATCCCGCGATCGAATAGCATCGATATGGTC
TPS41-SalIF	ACGCGTCGACATGCTGGTAATTACATCCAATTAC
TPS41-BamHIR−	CGGGATCCAACTGAATATCCTTTTCTTCATACTTG
TPS46-XhoIF	CCGCTCGAGATGGATTCCTCACCATTGTCTTCTATTG

TPS46-BamHIR−	CGGGATCCCGTACCATGCATGCAAGAGTAATAGGGAG
TPS47-XhoIF	CCGCTCGAGATGGCTTGCAGATGCATATTATCC
TPS47-BamHIR−	CGGGATCCCGTGGAGTATCACTCTTTGAAAACGTG
TPS48-XhoIF	CCGCTCGAGATGACTTCACTGGCTGGAAATG
TPS48-BamHIR−	CGGGATCCCGCGTCATCTGATTAATCCGAGTAG
TPS51-XhoIF	CCGCTCGAGATGGATGGATTTGGTGATGAGGCAGAG
TPS51-BamHIR−	CGGGATCCCGTTATAGATTTCACTCAATGATTCCTC
TPS52-XhoIF	CCGCTCGAGATGGTCGTTGGATTTGGTGGTGAAGCGGAG
TPS52-BamHIR−	CGGGATCCCGATTATAGATTTCACTCAATGATTCCTC
FPPS1-XhoIF	CCGCTCGAGATGGCTGATCTGAAGAAGAAATTTTTG
FPPS1-BamHIR−	CGGGATCCCGCTTCTGCCTCTTATAAATCTTTCCCAAG
GGPPS1-EcoRIF	CGGAATTCATGGCATTTTTAGCTACCATTTCTGG
GGPPS1-BamHIR−	CGGGATCCCAATTCTGTCGATTTGCAATATAACTAG
GGPPS2-XhoIF	CCGCTCGAGATGAGATCTATGAACCTTGTTGATTC
GGPPS2-SalIR−	ACGCGTCGACTGATTTTGACGATTAGCAATGTAATCTGC
GGPPS3-EcoRIF	CGGAATTCATGAGTCTTTCAACAACAATTACAAC
GGPPS3-BamHIR−	CGGGATCCCGATTCTCTCTGTAAGCAATATAATTTGC
SSU I-EcoRIF	CGGAATTCATGGCAAGAATTTTATATTTTTCCTCC
SSU I-BamHIR−	CGGGATCCCGAGACTCTTCAACAAGACTAGCAATTTG
SSU II-EcoRIF	CGGAATTCATGGTTTTCTCCATGGTGATGAGC
SSU II-BamHIR−	CGGGATCCCGGACTTGGCCATCAATGCTAAAACC
TPT1-EcoRIF	CGGAATTCATGGCCCATACTAAGTCAAATAGG
TPT1-BamHIR−	CGGGATCCCATTTTTGCCGATGAAGAACGAAATC
TPT2-EcoRIF	CGGAATTCATGTCTATGCGAAAAGGTGTAATC
TPT2-BamHIR−	CGGGATCCCATTTTTGCCGATGAAGAAGAAAATC
For cloning in pEAQHT and tobacco transient expression	
TPS10-NruIF	CTAGTCGCGAATGGCTTCTTCTTCTTCTACTAATAAG
TPS10-XmaIR	TCCCCCCGGGTATTGATATTTTGACAGATTCAATCAG
TPS16-NruIF	CTAGTCGCGAATGGAGCTGTGCACACAAACCG
TPS16-XhoIR	CCGCTCGAGCTATATAATAGGATCAATAAGTATCC
TPS18-NruIF	CTAGTCGCGATGATAATTGGCTATAGAATCAATTTC
dTPS18-NruIF	CTAGTCGCGATGGTCCCTTCAAAATATTCACTAAATG
TPS18-XmaIR	TCCCCCCGGGTCATCGATTATGGAGTGGTTTGTAAATGAC
TPS41-NruIF	CTAGTCGCGAATGCTGGTAATTACATCCAATTAC
dTPS41-NruIF	CTAGTCGCGAATGAGGAATTTGAGAGAAGAGATA
TPS41-XmaIR	TCCCCCCGGGGGCTTTCTGAAAAAGAACTTTGG
CPT1-NruIF	CTAGTCGCGAATGAGTTCTTTGGTTCTTCAATGTTG
CPT1-XmaIR	TCCCCCCGGGATATGTGTGTCCACCAAAACGTCTATG
CPT2-NruIF	CTAGTCGCGATGAACTCTTCAATAGTGTCTCAAC
dCPT2-NruIF	CTAGTCGCGATGCAAATTCTAAAGCTGCCTCTAACAATGTC

CPT2-XmaIR	TCCCCCCGGGTCAATATGTGTGTCCACCAAAACGTC
CPT6-NruIF	CTAGTCGCGATGAACTCTTTGTTTGTAGGGAGGCC
CPT6-XmaIR	TCCCCCCGGGTCAATATGTGTGTCCACCAAAACGTCTATG
CPS-NruIF	CTAGTCGCGAATGTCGATCTCAGCTTCTTTTTTAAG
dCPS-NruIF	CTAGTCGCGATGGGTTCGTTGCCGGTTATCAAGTGG
CPS-XmaIR	TCCCCCCGGGAACTACTCTTTCAAATAGCACTTTGGC
KS-NruIF	CTAGTCGCGAATGTCTGCTACCATTATTTTTCCTGCC
dKS-NruIF	CTAGTCGCGATGCGAGGACCGGATTTTGCTCTTCAATG
KS-XmaIR	TCCCCCCGGGATTGTAATCAGTAATTAGTTTTGAATCCC
GGPS1-NruIF	CTAGTCGCGAATGAGTCTTTCAACAACAATTACAAC
dGGPS1-NruIF	CTAGTCGCGATGGAGTTTAAAGAATACGTTCTTGAAAAG
GGPS1-XmaIR	TCCCCCCGGGATTCTCTCTGTAAGCAATATAATTTG
dTPT1-AgeIF	CCGACCGGTATGACAGGTTTTTCCAAGAATGAAAG
TPT1-XmaIR	TCCCCCCGGGTTTTTGCCGATGAAGAACGAAATC
dTPT2-AgeIF	CCGACCGGTATGACAGATCTTTCCAAGAATGAATC
TPT2-XmaIR	TCCCCCCGGGTTTTTGCCGATGAAGAAGAAAATC
dAtTPS19-NruIF	CTAGTCGCGATGCGTCCTTCGACGTACTTTTC
AtTPS19-XmaIR	TCCCCCCGGGTGAAAGGGGTATTGGGTGGACGTAC
dAtGFPS2-NruIF	CTAGTCGCGAATGATTCCACCAGAGGGAAAATGCAATG
AtGFPPS2-XmaIR	TCCCCCCGGGGTTGTGTCTGCAAGCGATGTAG
For qRT-PCR	
TPS3-QF	GTTCATGGGTTGTGCAGTAAAT
TPS3-QR	TAGGGCATGGAAATAGTGATGG
TPS4-QF	TGCTGTTCAAAGATGGGATACA
TPS4-QR	GTAGGGTAGGACGTTGATGTTG
TPS5-QF	GAGGTGACGTTTCCAAATCAATAC
TPS5-QR	GCTTCCCATGTCTCCTGTATC
TPS7-QF	AGCAGCACACCAACAAGA
TPS7-QR	GCTAATCCCACTGACCAGAAA
TPS8-QF	ACCTAGCAAGAATGGCACAA
TPS8-QR	CCTAGAGGAACGGATTCAAAGAG
TPS9-QF	ATCCAGAGATGGGATGCTAATG
TPS9-QR	CACGGTCCAGTTTACCTTCTT
TPS10-QF	GGTGGGAGCAAGGAAATACATATC
TPS10-QR	AAGTAACACTCAACCAGTCTGTCC
TPS12-QF	GAGACAGGTTGGTTGAGTGTTA
TPS12-QR	CATCAAAGAAGGAGGCCATTTG
TPS14-QF	GATTGTTGCGGGCGAATTTAT
TPS14-QR	GCTCTCTTTGTTGTTCGTGTTC
TPS16-QF	CAACGACGTGCATGGAATGTTAAG

TPS16-QR	TTCCTTGCTTCTACCCTCACAATAC
TPS17-QF	CCACCAATAGTTGTTGCTTCTTG
TPS17-QR	GCTTCTTCCTTTGTAACGCTATATTC
TPS18-QF	GTAATAGAAGAAAGTTGCTAAGGATGG
TPS18-QR	ATGACTTGATTGATATGATGCTTGAC
TPS19-QF	TCTTGAACTCCACAAAGCATCAC
TPS19-QR	CAAAGCAAGTTCCACCTCCTCT
TPS20-QF	TTCCCACTATGTATCCAACAAAGG
TPS20-QR	TGTTGCCATAGCCTGTATATTTCATC
TPS21-QF	AACTCAAGAGGTGGTTTCAAGATTG
TPS21-QR	CTCAGGTTCAGACACAACAGCTATG
TPS24-QF	AGTTTCACCCAGCATTCCAGTTC
TPS24-QR	CAACCCAGGCAGTATCGTATGAAG
TPS25-QF	ATGTTTCAGAGGATGTAGCTCG
TPS25-QR	GACCCCAAATCCATCTCCAG
TPS27-QF	CATGTTCTTCTTGGTTTGGCTCAAG
TPS27-QR	GATGAAGACACGTCACCTCTTTCC
TPS28-QF	CCATGAAGAAGATGATCTATACAAGG
TPS28-QR	CTAACATGTGCTGCTTCATACAAAC
TPS31-QF	GAAGTATCAAGGTGGTGGAAAGA
TPS31-QR	GCCTGAGAGTATTGAGGTTCAG
TPS32-QF	TTTGGACGATGGGAGTGTATG
TPS32-QR	CGGTGTAAACCTCCAGTTCTT
TPS33-QF	TAGGGATAGAGCAGTGGAGTGTTAC
TPS33-QR	ACCTCTGTATGGCATCGGTATAAAC
TPS35-QF	TCATGGAGAAGATATTTTGGAAGAGG
TPS35-QR	TCGTAGATAGAGATGAAGTATCGTGTC
TPS36-QF	GGTAAATCAGACCGCGTCTAC
TPS36-QR	GTTGTTCCAAGCGCCATAAAGGTAG
TPS37-QF	GGGCTTGTTAGTTCTGGAGTTT
TPS37-QR	GCTATGGAAGTGGACATGAGTG
TPS38-QF	GCTTCCTAGCTCTCTTCAACTC
TPS38-QR	GTTAGCCCACTGCTTCCTTAT
TPS39-QF	CTGGAGTTCATGTGGTCTTAGTT
TPS39-QR	ATCATCCCAAAGGCGAAGG
TPS40-QF	GGTAGGAGAAGGAGAAGGAGAAGG
TPS40-QR	GGAGTCGGTGAGAAACTCTATTGG
TPS41-QF	CACTTTGGATATGTGATTGTTGATTG
TPS41-QR	GCATGTGACAGACTTTATTGGTGTAC
TPS46-QF	AAGGTGGGATGCAAAGAATC

TPS46-QR	CATTTTGGCAACATCACCA
TPS47-QF	TTGGCCTCACTGATTACTTCGACG
TPS47-QR	CCATGCCCAAATATGACCCTTCC
TPS48-QF	ATGGAAGGATTTGAACAAAGAGTGC
TPS48-QR	AAAGCTTCGATTACAGCTTTCACG
TPS51-QF	ATGGATGGATTTGGTGATGAGGCAGAG
TPS51-QR	GATTTGGAAGGAGAAAGCACTAGC
TPS52-QF	ATGGTCGTTGGATTTGGTGGTG
TPS52-QR	GATTTGGAAGGAGAAAGCACTAGC
FPPS1-QF	CGC CGG ATT GTC CAG TAT AAA AC
FPPS1-QR	GGT CAG CAA AGC AGT CCA GAT AAT C
GGPPS1-QF	CAGCGGGTAAGGACCTAATAAC
GGPPS1-QR	TGCCTTTGCATAGTCGAAGTAG
GGPPS2-QF	CGCTACTTGCTTTCGCATTC
GGPPS2-QR	CCAGCTACTAACCCTTCAGTTC
GGPPS3-QF	GGGATGCACTTCTTGCATTAG
GGPPS3-QR	CTACAACCTGACCAGCTACAA
SSU I-QF	GGACAGCTAGAAGGCCAATATC
SSU I-QR	GCTCCACATGCATGAATTTCC
SSU II-QF	TCGAGTCCTCCGGGTTATTA
SSU II-QR	CACTCACCCATTTCACCATATTTC
TPT1-QF	GCGTCCGAGAAGGAGATTAATAG
TPT1-QR	GCTCAGAGGATTTAGTCACATCA
TPT2-QF	TTCGTGCTGTTGGAGAAGTAG
TPT2-QR	GCTCTTCCAATCCAGTATCACA
CPT1-QF	CAAAGGGCAACAAAGGACTTCACC
CPT1-QR	CAGGATTTGGACACTTGCTTTCAAG
CPT2-QF	GAACAAGAACTTGAAAGCAAGTGTC
CPT2-QR	CGTCTATGCCTTTGTTGAAAGTTAAG
CPT6-QF	TACCACCAAGTGTGCTAAATTCCC
CPT6-QR	ATGAGAGCTTCTTCTCCAAAGTCAG
Ubi-QF	CAGCTCGCTTTCTCAGTTCC
Ubi-QR	AGCTCGACAAACAGACTGGA

[1]+, has a stop codon in the sequence; −, does not contain a stop codon in the sequence. d, for truncating the N-terminal transit peptide. opti, codon optimized version.

[2]Restriction sites are underlined.

Table S2 A list of terpene compounds identified from Figs 3-5 and 10.

Identified terpenes[1]	MS similarity to NIST library	Identification method	Retention indices[2]	Reference retention indices[3]	Source of reference
**29. Isoprene**	99%	b, c	n.c.	n.r.	Standard
α-Thujene	97%	a, b, c	929.7	929.7	Mandarin oil
α-Pinene	98%	a, b, c	937.7	937.7	Standard
Unknown 5	81% to 3-carene	-	971.1	3-carene RI=1011.0	Standard
1,3,5-Cycloheptatriene, 3,7,7-trimethyl	97%	a, b	977.0	972.6	NIST 2014
β-Pinene	97%	a, b, c	981.6	981.6	Standard
Isolimonene	95%	a, b, c	986.1	986.1	Standard
**25. β-Myrcene**	93%	a, b, c	990.5	990.5	Standard
(+)-2-Carene	96%	a, b, c	1002.9	1002.9	Standard
α-Phellandrene	97%	a, b, c	1010.5	1010.5	Standard
α-Terpinene	96%	a, b, c	1020.6	1020.6	Standard
m-cymene	97%	a, b, c	1028.1	1028.1	Standard
β-Phellandrene	95%	a, b, c	1037.4	1037.4	SlPHS1 (Schilmiller *et al.*, 2009)
**26. β-*cis*-Ocimene**	94%	a, b, c	1038.8	1038.8	Standard

**27. β-*trans*-Ocimene**	99%	a, b, c	1049.3	1049.3	Standard
α-Ocimene	90%	a, b	1057.8	1056	Jordán *et al*. (2013)
γ-Terpinene	98%	a, b, c	1062.8	1062.8	Standard
Terpinolene	95%	a, b, c	1088.8	1088.8	Standard
Unknown 6	87% to phellandral	-	1096.1	Phellandral RI=1273	Babushok *et al*. (2011)
**28. Linalool**	93%	a, b, c	1099.6	1099.6	Standard
trans-p-Mentha-2,8-dienol	95%	a, b	1126.7	1127.5	Babushok *et al*. (2011)
α-Terpineol	93%	a, b, c	1199.5	1199.5	Standard
nerol	95%	a, b, c	1230.4	1230.4	Standard
geraniol	90%	a, b, c	1257.0	1257.0	Standard
δ-Elemene	95%	a, b	1332.9	1333.8	Zizovic *et al*. (2007)
**12. α-Cedrene**	92%	a, b, c	1394.0	1394.0	Standard
**4. β-Elemene**	96%	a, b, c	1395.5	1395.5	Standard
**13. *cis*-α-Bergamotene**	90%	a, b	1419.6	1419.5	NIST 2014
**20. (*Z*)-β-Farnesene**	94%	a, b, c	1429.6	1429.6	Standard
β-Caryophyllene	95%	a, b, c	1432.0	1432.0	Standard
**6. (*E*)-β-Famesene**	96%	a, b, c	1454.8	1454.8	Standard
α-Humulene	95%	a, b, c	1468.6	1468.6	Standard

**14. β-Acoradiene**	91%	a, b	1469.8	1466	Babushok *et al*. (2011)
**15. Unknown 2**	89% to β-acoradiene	-	1478.2	β-Acoradiene RI=1466	Babushok *et al*. (2011)
**16. Unknown 3**	88% to trans-Sesquisabinene hydrate	-	1483.5	trans-Sesquisabinene hydrate RI=1581	Babushok *et al*. (2011)
Germacrene D	90%	a, b, c	1493.0	1493.0	Ginger oil
**18. *cis*-muurola-3,5-diene**	93% to *cis*-muurola-3,5-diene<br>92% to α-cubebene	a, b	1494.0	n.r.<br>α-cubebene RI=1351	Babushok *et al*. (2011)
**1. (*E*,*E*)-α-Farnesene**	97%	a, b, c	1505.3	1505.3	Standard
**7. β-Bisabolene**	94%	a, b, c	1513.3	1513.3	Ginger oil
**3. Guaia-1(10),11-diene**	94%	a, b	1515.3	1515	Novak *et al*. (2010)
**17. β-Curcumene**	93%	a, b	1518.6	1516	Kahriman *et al*. (2011)
**22. δ-Cadinene**	92%	a, b	1523.1	1524	Novak *et al*. (2010)
**19. *Z*-Nerolidol**	94%	a, b, c	1533.1	1533.1	Standard
**8. Unknown 1**	84% to α-Bergamotene	-	1533.8	α-Bergamotene RI=1434	Babushok *et al*. (2011)
**9. α-Bisabolene**	90%	a, b	1545.5	1541	Babushok *et al*. (2011)
**2. Elemol**	96%	a, b	1557.7	1557	Pinheiro *et al*. (2013)
**5. *E*-Nerolidol**	95%	a, b, c	1563.4	1563.4	Standard

**10. α-Eudesmol**	90%	a, b	1663.2	1664	Watanabe *et al*. (2005)
**11. (*Z*,*Z*)-Farnesol**	95%	a, b, c	1670.6	1670.6	SlCPT6 (Akhtar *et al*., 2013)
**21. α-Bisabolol**	93%	a, b, c	1694.8	1694.8	Standard
**23. Unknown 4**	85% to α-springene	-	2059.7	α-springene RI=2019	Setzer *et al*. (2006)
**24. *ent*-Kaurene**	95%	a, b, c	2079.5	2079.5	SlTPS40 (Falara *et al*., 2011)

[1]Compounds listed in order of elution from the Rxi-5Sil MS column, the enzymatic terpene products are shown in bold. [2]Retention indices relative to C_7-C_{40} n-alkanes on the Rxi-5Sil MS column. [3]Reference retention indices were obtained using the similar nonpolar column.

a, identification based on retention index; b, identification based on comparison with mass spectral data; c, identification based on comparison with authentic compounds; n.c. not calculated; n.r. no reference available from the similar column.

Akhtar TA, Matsuba Y, Schauvinhold I, Yu G, Lees HA, Klein SE, Pichersky E. 2013. The tomato *cis*-prenyltransferase gene family. *Plant J* **73**(4): 640-652.

Babushok VI, Linstrom PJ, Zenkevich IG. 2011. Retention indices for frequently reported compounds of plant essential oils. *J Phys Chem Ref Data* **40**(4).

Falara V, Akhtar TA, Nguyen TT, Spyropoulou EA, Bleeker PM, Schauvinhold I, Matsuba Y, Bonini ME, Schilmiller AL, Last RL, et al. 2011. The tomato terpene synthase gene family. *Plant Physiol* **157**(2): 770-789.

Jordan MJ, Margaria CA, Shaw PE, Goodner KL. 2003. Volatile components and aroma active compounds in aqueous essence and

fresh pink guava fruit puree (*Psidium guajava* L.) by GC-MS and multidimensional GC/GC-O. *J Agric Food Chem* **51**(5): 1421-1426.

Kahriman N, Tosun G, Terzioglu S, Karaoglu SA, Yayli N. 2011. Chemical composition and antimicrobial activity of the essential oils from the flower, leaf, and stem of *Senecio pandurifolius*. *Rec Nat Prod* **5**(2): 82-91.

Novak J, Novak S, Bitsch C, Franz CM. 2000. Essential oil composition of underground parts of *Valeriana celtica* ssp from Austria and Italy. *Flavour Frag J* **15**(1): 40-42.

Pinheiro PF, de Queiroz VT, Rondelli VM, Costa AV, Marcelino TD, Pratissoli D. 2013. Insecticidal activity of citronella grass essential oil on *Frankliniella schultzei* and *Myzus persicae*. *Cienc Agrotec* **37**(2): 138-144.

Schilmiller AL, Schauvinhold I, Larson M, Xu R, Charbonneau AL, Schmidt A, Wilkerson C, Last RL, Pichersky E. 2009. Monoterpenes in the glandular trichomes of tomato are synthesized from a neryl diphosphate precursor rather than geranyl diphosphate. *Proc Natl Acad Sci U S A* **106**(26): 10865-10870.

Setzer WN, Noletto JA, Lawton RO. 2006. Chemical composition of the floral essential oil of *Randia matudae* from Monteverde, Costa Rica. *Flavour Frag J* **21**(2): 244-246.

Watanabe Y, Mihara R, Mitsunaga T, Yoshimura T. 2005. Termite repellent sesquiterpenoids from *Callitris glaucophylla* heartwood. *J Wood Sci* **51**(5): 514-519.

Zizovic I, Stamenic M, Ivanovic J, Orlovic A, Ristic M, Djordjevic S, Petrovic SD, Skala D. 2007. Supercritical carbon dioxide extraction of sesquiterpenes from *Valerian* root. *J Supercrit Fluid* **43**(2): 249-258.

Table S3 A list of tomato terpene synthase genes, their characteristics, and the enzymes they encode.

Chromosome	Locus	TPS	TPS-clade	No. of Exons	Full-length cDNA obtained	Protein size (aa)	Subcellular localization	Substrate	Main product
1	Solyc01g101170	31	a	6	Yes	555	Cytosol[c]	*E*,*E*-FPP	Viridiflorene[a]
	Solyc01g101180	32	a	6	Yes	548	Cytosol[c]	*E*,*E*-FPP<br>*Z*,*Z*-FPP	Viridiflorene[b]<br>Unidentified[b]
	**Solyc01g101190**	**33**	**a**	**6**	**Yes**	**552**	**Cytosol**[c]	**_E_,_E_-FPP**<br>**_Z_,_Z_-FPP**	**Guaia-1(10),11-diene**[c]<br>**β-Acoradiene**[c]
	**Solyc01g101210**	**35**	**a**	**6**	**Yes**	**556**	**Cytosol**[c]	**_E_,_E_-FPP**<br>**_Z_,_Z_-FPP**	**Guaia-1(10),11-diene**[c]<br>**(_Z_,_Z_)-Farnesol** [c]
	Solyc01g105870	3	b	7	Yes	607	Plastid[c]	GPP	Camphene, tricyclene[b]
	Solyc01g105880	4 (MTS2)	b	7	Yes	590	Plastid[c]	GPP	β-Phellandrene[d]
	Solyc01g105890	5 (MTS1)	b	8	Yes	609	Plastid[c]	GPP	Linalool[d]
	Solyc01g105920	7	b	7	Yes	592	Plastid[c]	GPP	β-Myrcene, limonene[b]
	Solyc01g105960	8	b	7	Yes	597	Cytosol[c]	GPP<br>NPP	1,8-Cineole[b]<br>1,8-Cineole[b]
2	Solyc02g079840	38	b	7	Yes	546	Cytosol[c]	*E*,*E*-FPP	α-Bergamotene[b]
	**Solyc02g079890**	**25**	**b**	**7**	**Yes**	**547**	**Cytosol**[c]	**GPP**	**β-Ocimene**[c]
	**Solyc02g079910**<br>**Solyc00g154480**	**27**	**b**	**7**	**Yes**	**554**	**Cytosol**[c]	**_E_,_E_-FPP**	**α-Farnesene**[c]
3	Solyc03g006550	46	e/f	11	Yes	821	Cytosol[c]	GGPP	Geranyllinalool[e]
	**Solyc03g007730**	**47**	**b**	**7**	**Yes**	**562**	**Cytosol**[c]	**DMAPP**	**Isoprene**[c]
4	**Solyc04g051620**	**48**	**a**	**8**	**Yes**	**560**	**Cytosol**[c]	**_E_,_E_-FPP**<br>**_Z_,_Z_-FPP**	**Hedycaryol (Elemol)**[c]<br>**Unidentified**[c]
	**Solyc04g054380**	**28**	**a**	**7**	**Yes**	**554**	**Cytosol**[c]	**_E_,_E_-FPP**<br>**_Z_,_Z_-FPP**	**Hedycaryol (Elemol)**[c]<br>**Unidentified**[c]
6	Solyc06g059885	9 (Sst1)	a	7	Yes	548	Cytosol[c]	*E*,*E*-FPP	Germacrene[f]

	**Solyc06g059910**<br>**Solyc06g059920**	**10**	**a**	**7**	**Yes**	**556**	**Cytosol[c]**	***Z*,*Z*-FPP**	**α-Bisabolol[c]**
	Solyc06g059930	12 (CAHS)	a	7	Yes	548	Cytosol[c]	*E*,*E*-FPP	β-Caryophyllene, α-humulene[g]
	**Solyc06g060180**	**36**	**a**	**7**	**Yes**	**591**	**Mitochondria[b]**	***Z*,*Z*-FPP**	***cis*-Muurola-3,5-diene[c]**
	Solyc06g084240	40 (CPS1)	c	15	Yes	800	Plastid[c]	GGPP	Copalyl diphosphate[h]
7	**Solyc07g008680**<br>**Solyc07g008690**	**16**	**a**	**7**	**Yes**	**553**	**Cytosol[c]**	***E*,*E*-FPP**	**δ-Cadinene[c]**
	**Solyc07g052120**<br>**Solyc07g052130**	**51**	**a**	**7**	**Yes**	**550**	**Cytosol[c]**	***E*,*E*-FPP**<br>***Z*,*Z*-FPP**	***E*-Nerolidol[c]**<br>**α-Bisabolol[c]**
	**Solyc07g052140**<br>**Solyc07g052150**	**52**	**a**	**7**	**Yes**	**551**	**Cytosol[c]**	***E*,*E*-FPP**<br>***Z*,*Z*-FPP**	***E*-Nerolidol[c]**<br>**α-Bisabolol[c]**
	Solyc07g066670<br>Solyc07g066675	24 (KS)	e/f	15	Yes	820	Plastid[c]	CPP	*ent*-Kaurene[b]
8	Solyc08g005640	21	e/f	14	Yes	784	Plastid[c]	NNPP	Lycosantalene[i]
	Solyc08g005665	20 (PHS1)	e/f	14	Yes	778	Plastid[c]	NPP	β-Phellandrene[j]
	Solyc08g005670	19	e/f	14	Yes	778	Plastid[c]	NPP	β-Myrcene, β-ocimene[i]
	**Solyc08g005710**	**41**	**c**	**15**	**Yes**	**780**	**Mitochondria[c]**	**GGPP**	**Copalyl diphosphate[c]**
	**Solyc08g005720**	**18**	**e/f**	**14**	**Yes**	**773**	**Mitochondria[c]**	**NNPP**	**Unidentified[c]**
9	Solyc09g092470	14	a	7	Yes	568	Cytosol[b]	*E*,*E*-FPP<br>*Z*,*Z*-FPP	β-Bisabolene[b]<br>α-Bisabolene[b]
10	Solyc10g005390	39	g	7	Yes	563	Cytosol[c]	GPP<br>*E*,*E*-FPP	Linalool[b]<br>*E*-Nerolidol[b]
	Solyc10g005410	37	g	7	Yes	560	Cytosol[c]	GPP<br>*E*,*E*-FPP	Linalool[b]<br>*E*-Nerolidol[b]
12	Solyc12g006570	17	a	7	Yes	554	Cytosol[c]	*E*,*E*-FPP	Valencene[a]

[a]Bleeker *et al*. (2011). [b]Falara *et al*. (2011). [c]This work. [d]van Schie *et al*. (2007). [e]Falare *et al*. (2014). [f]Colby *et al*. (1998). [g]Schilmiller *et al*. (2010). [h]Bensen and Zeevaart, (1990). [i]Matsuba *et al*. (2013). [j]Schilmiller *et al*. (2009).

TPS gene numbers are adopted from Falara *et al*. (2011), terpene synthases characterized in this work are shown in bold.

Bensen RJ, Zeevaart JAD. 1990. Comparison of *ent*-kaurene synthetase A-activity and B-activity in cell-free-extracts from young tomato fruits of wild-type and *gib-1*, *gib-2*, and *gib-3* tomato plants. *Journal of Plant Growth and Regulation* **9**(4): 237-242.

Bleeker PM, Spyropoulou EA, Diergaarde PJ, Volpin H, De Both MT, Zerbe P, Bohlmann J, Falara V, Matsuba Y, Pichersky E, et al. 2011. RNA-seq discovery, functional characterization, and comparison of sesquiterpene synthases from *Solanum lycopersicum* and *Solanum habrochaites* trichomes. *Plant Molecular Biology* **77**(4-5): 323-336.

Colby SM, Crock J, Dowdle-Rizzo B, Lemaux PG, Croteau R. 1998. Germacrene C synthase from *Lycopersicon esculentum* cv. VFNT cherry tomato: cDNA isolation, characterization, and bacterial expression of the multiple product sesquiterpene cyclase. *Proceedings of the National Academy of Sciences, USA* **95**(5): 2216-2221.

Falara V, Akhtar TA, Nguyen TT, Spyropoulou EA, Bleeker PM, Schauvinhold I, Matsuba Y, Bonini ME, Schilmiller AL, Last RL, et al. 2011. The tomato terpene synthase gene family. *Plant Physiology* **157**(2): 770-789.

Falara V, Alba JM, Kant MR, Schuurink RC, Pichersky E. 2014. Geranyllinalool synthases in solanaceae and other angiosperms constitute an ancient branch of diterpene synthases involved in the synthesis of defensive compounds. *Plant Physiology* **166**(1): 428-441.

Matsuba Y, Nguyen TT, Wiegert K, Falara V, Gonzales-Vigil E, Leong B, Schafer P, Kudrna D, Wing RA, Bolger AM, et al. 2013. Evolution of a complex locus for terpene biosynthesis in *Solanum*. *Plant Cell* **25**(6): 2022-2036.

Schilmiller AL, Miner DP, Larson M, McDowell E, Gang DR, Wilkerson C, Last RL. 2010. Studies of a biochemical factory: tomato trichome deep expressed sequence tag sequencing and proteomics. *Plant Physiology* **153**(3): 1212-1223.

Schilmiller AL, Schauvinhold I, Larson M, Xu R, Charbonneau AL, Schmidt A, Wilkerson C, Last RL, Pichersky E. 2009. Monoterpenes in the glandular trichomes of tomato are synthesized from a neryl diphosphate precursor rather than geranyl diphosphate. *Proceedings of the National Academy of Sciences, USA* **106**(26): 10865-10870.

van Schie CC, Haring MA, Schuurink RC. 2007. Tomato linalool synthase is induced in trichomes by jasmonic acid. *Plant Molecular Biology* **64**(3): 251-263.

Table S4 A list of tomato terpene synthase pseudogenes.

Chromosome	Locus	TPS	No. of Exons	Type of mutation	Putative translated protein (aa)
1	Between Solyc01g101190 and Solyc01g101210	34	(-)3	Deletion of exons	No in frame start codon was found
	Solyc01g101220	30	(-)3(-)1	Deletion of exons	No in frame start codon was found
	Solyc01g105850 Solyc01g105860	1	7	Aberrant splicing, incorrect 3' splice sequence at the end of intron 4, cloned cDNAs skip exon 5 and part of exon 6	400(-)
	Solyc01g105900	6	7	Deletion of about 80nt in exon 3 to cause frame shift	165(-)
	Solyc01g105910	2	3(-)	Deletion of exons and stop codons in frame	131(-)
	Solyc01g105930 Solyc01g105940 Solyc01g105950	22	7	Large insertion in exon 2 and numerous stop codons in frame	52(-)
2	Solyc02g079900 Solyc02g079895	26	(-)4	Deletion of exons and mutation of 3' splice site	No in frame start codon was found
**3**	**Solyc03g112175**	**49**	**(-)2**	**Only the last two exons were found.**	**(-)92**
**4**	**Between Solyc04g005100 and Solyc04g005120**	**50**	**2(-)2**	**Deletion of exons**	**51(-)**
5	Solyc05g026590 Solyc05g026600	43	7	Insertion of an extra T in first exon to cause frame shift	30(-)
6	Between Solyc06g059885 and Solyc06g059880	23	(-)1	Deletion of exons	No in frame start codon was found
	Part of Solyc06g059930	11	2(-)2(-)	Deletion of exons	50(-)
	Solyc06g060010 Solyc06g060013	13	7	Insertion in exon 2 to cause frame shift	156(-)
7	Solyc07g051940	15	7	The end of the first exon has a deletion including	29(-).

				the splice site, so the intron is not spliced correctly, cloned cDNA has stop codon in frame	
9	Solyc09g065230	44	(-)3(-)	Deletion of exons	No in frame start codon was found
10	Solyc10g005420	42	(-)4(-)	Deletion of exons to cause frame shift	15(-)
	**Solyc10g052745**	**53**	**(-)2**	**Deletion of exons**	**No in frame start codon was found**
12	Solyc12g019240	29	(-)1(g)2(g)	Deletion of exons	No in frame start codon was found

TPS gene numbers and type of mutation are adopted from Falara *et al*. (2011), the new terpene synthase pseudogenes identified in this work are shown in bold.

Table S5 A list of the *trans*-prenyltransferase genes in tomato.

TPT	Locus	No. of exons	Predicted product[1]	Main Product	Subcellular Localization
FPPS1	Solyc12g015860	12	C15	FPP[a]	Cytosol[b]
**GGPPS1**	Solyc11g011240	1	C20	GGPP[b, c]	Plastid[b]
**GGPPS2**	Solyc04g079960	1	C20	GGPP[b, c]	Plastid[b]
SPPS	Solyc07g061990	6	C20	SPP[d]	Plastid[d]
DPPS	Solyc08g023470	12	≥C30	DPP[d]	Cytosol[d]
**GGPPS3**	Solyc02g085700	1	C20	GGPP[b]	Plastid[b]
**TPT1**	Solyc02g085710	1	C20	n.d.[b]	Mitochondria[b]
**TPT2**	Solyc02g085720	1	C20	n.d.[b]	Mitochondria[b]
**SSU I**	Solyc07g064660	1	C20	n.d.[b]	Plastid[b]
**SSU II**	Solyc09g008920	2	C20	n.d.[b]	Plastid[b]

[1]Prediction of *trans*-prenyltransferase activity was made by the well-established "three floors" model of TPT product chain-length determination mechanism (Wang *et al*., 2016). [a]Gaffe *et al*. (2000). [b]This work. [c]Ament *et al*. (2006). [d]Jones *et al*. (2013). n.d., not detected. Enzymes characterized in this work are shown in bold.

Ament K, Van Schie CC, Bouwmeester HJ, Haring MA, Schuurink RC. 2006. Induction of a leaf specific geranylgeranyl pyrophosphate synthase and emission of (*E*,*E*)-4,8,12-trimethyltrideca-1,3,7,11-tetraene in tomato are dependent on both jasmonic acid and salicylic acid signaling pathways. *Planta* **224**(5): 1197-1208.

Gaffe J, Bru JP, Causse M, Vidal A, Stamitti-Bert L, Carde JP, Gallusci P. 2000. *LeFPS1*, a tomato farnesyl pyrophosphate gene highly expressed during early fruit development. *Plant Physiology* **123**(4): 1351-1362.

Jones MO, Perez-Fons L, Robertson FP, Bramley PM, Fraser PD. 2013. Functional characterization of long-chain prenyl diphosphate synthases from tomato. *Biochemical Journal* **449**(3): 729-740.

Wang CY, Chen QW, Fan DJ, Li JX, Wang GD, Zhang P. 2016. Structural analyses of short-chain prenyltransferases identify an evolutionarily conserved GFPPS clade in Brassicaceae plants. *Molecular Plant* **9**(2): 195-204.

Table S6 A list of terpene compounds produced by all the functional tomato terpene synthases.

TPS	Substrate	Products	Reference
3	**GPP**	**Camphene**, tricyclene, β-Myrcene, limonene, α-pinene, sabinene, β-pinene, unidentified	Falara *et al*. (2011)
4	**GPP**	**β-Phellandrene**, β-myrcene, sabinene	Van Schie *et al*. (2007)
5	**GPP**<br>*E*,*E*-FPP	**Linalool**<br>***E*-Nerolidol**	Van Schie *et al*. (2007)
7	**GPP**	**β-Myrcene**, limonene, α-pinene, β-pinene, linalool, sabinene	Falara *et al*. (2011)
8	**GPP**<br>**NPP**	**1,8-Cineole**, α-pinene<br>**1,8-Cineole**, α-pinene	Falara *et al*. (2011)
9	GPP<br>***E*,*E*-FPP**<br>*Z*,*Z*-FPP	**β-Myrcene**, limonene, α-pinene, (+)-2-carene, α-terpinene, terpinolene<br>**Germacrene C**, germacrene A, germacrene B, germacrene D<br>**Germacrene C**, α-humulene, β-bisabolene, germacrene A, germacrene B	Bleeker *et al*. (2011)<br>Colby *et al*. (1998)<br>Bleeker *et al*. (2011)
**10**	***Z*,*Z*-FPP**	**α-Bisabolol**	This work
12	GPP<br>***E*,*E*-FPP**<br>*Z*,*Z*-FPP	**β-Myrcene**, limonene, terpinolene, *E*-β-ocimene<br>**β-Caryophyllene, α-humulene**<br>**γ-Curcumene**, **β-bisabolene**, Z-γ-bisabolene	Bleeker *et al*. (2011)<br>Schilmiller *et al*. (2010)
14	***E*,*E*-FPP**<br>*Z*,*Z*-FPP	**β-Bisabolene**, *E*-nerolidol, *E*-γ-bisabolene, *Z*-γ-bisabolene, *E*-α-bisabolene<br>***E*-α-Bisabolene**, β-bisabolene, *E*-γ-bisabolene, *Z*-γ-bisabolene,	Falara *et al*. (2011)
**16**	***E*,*E*-FPP**	**δ-Cadinene**	This work
17	GPP<br>***E*,*E*-FPP**<br>*Z*,*Z*-FPP	**β-Myrcene**, (+)-2-carene, limonene, terpinolene, *E*-β-ocimene, *Z*-β-ocimene, γ-terpinene, linalool<br>**Valencene**, γ-gurjunene, *E*-β-farnesene, α-farnesene<br>**Z-γ-bisabolene**, *E*-α-bergamotene, *Z*-α-bergamotene, *E*-γ-bisabolene, β -bisabolene	Bleeker *et al*. (2011)
**18**	**NNPP**	**Unidentified**	This work
19	**NPP**	**β-Ocimene**, β-myrcene	Matsuba *et al*. (2013)
20	**NPP**	**β-Phellandrene**, (+)-2-carene, limonene, α-phellandrene, α-terpinene	Schilmiller *et al*. (2009)
21	**NNPP**	**Lycosantalene**	Matsuba *et al*. (2013)
24	**CPP**	***Ent*-Kaurene**	Falara *et al*. (2011)
**25**	**GPP**	***E*-β-ocimene**, *Z*-β-ocimene, linalool, β-Myrcene	This work
**27**	***E*,*E*-FPP**	**α-Farnesene**	This work
**28**	***E*,*E*-FPP**<br>*Z*,*Z*-FPP	**Elemol (hedycaryol)**<br>**Unidentified**, β-bisabolene, *E*-α-bisabolene, α-eudesmol, *Z*,*Z*-farnesol	This work
31	***E*,*E*-FPP**	**Viridiflorene**	Bleeker *et al*. (2011)
32	***E*,*E*-FPP**<br>*Z*,*Z*-FPP	**Viridiflorene**<br>**Unidentified**	Falara *et al*. (2011)
**33**	***E*,*E*-FPP**<br>*Z*,*Z*-FPP	**Guaia-1(10),11-diene**<br>**β-Acoradiene**, β-curcumene, α-cedrene, *Z*-α-bergamotene, unidentified	This work
**35**	***E*,*E*-FPP**<br>*Z*,*Z*-FPP	**Guaia-1(10),11-diene**, β-elemene (germacrene A)<br>***Z*,*Z*-Farnesol**, β-acoradiene	This work
**36**	***Z*,*Z*-FPP**	***cis*-Muurola-3,5-diene**, β-acoradiene	This work

37	GPP<br>***E*,*E*-FPP**	**Linalool**<br>***E*-Nerolidol**	Falara *et al*. (2011)
38	***E*,*E*-FPP**	***E*-α-Bergamotene**	Falara *et al*. (2011)
39	GPP<br>***E*,*E*-FPP**	**Linalool**<br>***E*-Nerolidol**	Falara *et al*. (2011)
40	**GGPP**	**Copalyl diphosphate**	Bensen and Zeevaart, (1990)
**41**	**GGPP**	**Copalyl diphosphate**	This work
46	**GGPP**	**Geranyllinalool**	Falara *et al*. (2014)
**47**	**DMAPP**	**Isoprene**	This work
**48**	***E*,*E*-FPP**<br>*Z*,*Z*-FPP	**Elemol (hedycaryol)**<br>**Unidentified**	This work
**51**	***E*,*E*-FPP**<br>*Z*,*Z*-FPP	***E*-Nerolidol**, *E*-β-farnesene<br>**α-Bisabolol**, *Z*-nerolidol, *E*-α-bisabolene, β-bisabolene, *Z*-β-farnesene	This work
**52**	***E*,*E*-FPP**<br>*Z*,*Z*-FPP	***E*-Nerolidol**, *E*-β-farnesene<br>**α-Bisabolol**, *Z*-nerolidol, *E*-α-bisabolene, β-bisabolene, *Z*-β-farnesene	This work

The main product of each TPS enzyme using the corresponding substrate is shown in bold. The preferred substrates are shown in bold, considering their subcellular localizations. Structures of terpene compounds can be found in Fig. 11. TPS enzymes characterized in this work are shown in bold. Elemol is the thermal breakdown product of (+)-hedycaryol (Hattan *et al*., 2016); β-elemene and δ-elemene are the thermal rearrangement of germacrene A and germacrene C, respectively (Colby *et al*., 1998). References can be found in Table S3.

Table S7 A list of Arabidopsis terpene synthase genes, their characteristics, and the enzymes they encode.

Chromosome	AGI No.	TPS	TPS-clade	No. of Exons	Protein size (aa)	Subcellular localization	Substrate	Main product	Reference
1	At1g31950	29	a	7	607	M/P	Unknown	-	-
	At1g33750	22	a	6	603	M/P	*E*,*E*-FPP	(*E*)-β-Farnesene, α-farnesene	Wang *et al*. (2016)
	At1g48800	28	a	7	603	M/P	Unknown	-	-
	At1g61120	4	e/f	12	877	C	GGPP	Geranyllinalool	Herde *et al*. (2008)
	At1g61680	14	g	7	569	P	GPP	Linalool	Chen *et al*. (2003)
	At1g66020	26	a	7	598	M/P	GGPP	Unknown diterpenes	Wang *et al*. (2016)
	At1g70080	6	a	7	611	M/P	GGPP	Dolabelladienol	Wang *et al*. (2016)
	At1g79460-KS1	32	e/f	14	785	P	*Ent*-CPP	*Ent*-kaurene	Yamaguchi *et al*. (1998); Helliwell *et al*. (2001)
2	At2g23230	5	a	7	606	M/P	Unknown	-	-
	At2g24210	10	b	7	591	P	GPP	(E)-β-ocimene, myrcene	Bohlmann *et al*. (2000)
3	At3g14490	17	a	7	601	P	GFPP	Sesquiterpene blend	Huang *et al*. (2017)
	At3g14520	18	a	7	605	P	GFPP	(+)-thalianatriene	Shao *et al*. (2017); Chen *et al*. (2019)
	At3g14540	19	a	7	602	P	GFPP	(−)-retigeranin B	Shao *et al*. (2017); Chen *et al*. (2019)
	At3g25810	24	b	7	598	P	GPP	Monoterpene blend	Chen *et al*. (2003)
	At3g25820	27	b	7	600	P	GPP	1,8-Cineole	Chen *et al*. (2004); Roos *et al*. (2015)
	At3g25830	23	b	7	600	P	GPP	1,8-Cineole	Chen *et al*. (2004)
	At3g29110	16	a	7	595	P	Unknown	-	-
	At3g29190	15	a	7	601	P	Unknown	-	-
	At3g29410	25	a	7	603	P	GFPP	(-)-ent-quiannulatene, (-)-variculatriene A	Huang *et al*. (2017); Chen *et al*. (2019)
	At3g32030	30	a	7	604	P	GFPP	(+)-Astallatene	Huang *et al*. (2017); Chen *et al*. (2019)
4	At4g02780-CPS	31	c	15	802	P	GGPP	*Ent*-CPP	Sun and Kamlya, 1997; Helliwell *et al*. (2001)

	At4g13280	12	a	8	524	C	*E*,*E*-FPP	(Z)-γ-bisabolene	Ro *et al*. (2006)
	At4g13300	13	a	7	554	C	*E*,*E*-FPP	(Z)-γ-bisabolene	Ro *et al*. (2006)
	At4g15870	1	a	7	598	P	Unknown	-	-
	At4g16730-Ws	2	b	7	589	P	GPP<br>*E*,*E*-FPP	(E)-β-ocimene, myrcene<br>(E,E)-α-farnesene	Huang *et al*. (2010)
	At4g16740	3	b	7	565	P	GPP<br>*E*,*E*-FPP	(E)-β-ocimene, myrcene<br>(E,E)-α-farnesene	Fäldt *et al*. (2003)
	At4g20210	8	a	7	600	P	GGPP	Rhizathalene A	Vaughan *et al*. (2013)
	At4g20230	9	a	7	607	M/P	GGPP	Unidentified diterpene	Wang *et al*. (2016)
	At4g20200	7	a	7	603	P	Unknown	-	-
5	At5g23960	21	a	7	547	C	*E*,*E*-FPP	(E)-β-caryophyllene	Chen *et al*. (2003)
	At5g44630	11	a	7	557	C	*E*,*E*-FPP	Several sesquiterpenes	Tholl *et al*. (2005)
	At5g48110-Cvi	20	a	8	592	P	GGPP	Dolathaliatriene, dolabelladienol	Wang *et al*. (2016)

TPS gene numbers are adopted from Aubourg *et al*. (2002). Underlined letters indicate the experimentally proved subcellular localizations and the other localizations were predicted by the TargetP and ChloroP programs. Ws, *Arabidopsis thaliana* Wassilewskija ecotype; Cvi, *A. thaliana* Cape Verde Islands ecotype. P, plastid; M, mitochondria; C, cytosol.

Aubourg S, Lecharny A, Bohlmann J. 2002. Genomic analysis of the terpenoid synthase (AtTPS) gene family of *Arabidopsis thaliana*. *Molecular Genetics and Genomics* **267**(6): 730-745.

Bensen RJ, Zeevaart JAD. 1990. Comparison of *ent*-kaurene synthetase A-activity and B-activity in cell-free-extracts from young tomato fruits of wild-type and *gib-1*, *gib-2*, and *gib-3* tomato plants. *Journal of Plant Growth Regulation* **9**(4): 237-242.

Bohlmann J, Martin D, Oldham NJ, Gershenzon J. 2000. Terpenoid secondary metabolism in *Arabidopsis thaliana*: cDNA cloning, characterization, and functional expression of a myrcene/(*E*)-beta-ocimene synthase. *Archives of Biochemistry and Biophysics* **375**(2): 261-269.

Chen F, Ro DK, Petri J, Gershenzon J, Bohlmann J, Pichersky E, Tholl D. 2004. Characterization of a root-specific Arabidopsis

terpene synthase responsible for the formation of the volatile monoterpene 1,8-cineole. *Plant Physiology* **135**(4): 1956-1966.

Chen F, Tholl D, D'Auria JC, Farooq A, Pichersky E, Gershenzon J. 2003. Biosynthesis and emission of terpenoid volatiles from Arabidopsis flowers. *Plant Cell* **15**(2): 481-494.

Chen QW, Jiang T, Liu YX, Liu HL, Zhao T, Liu ZX, Gan XC, Hallab A, Wang XM, He J, *et al.* 2019. Recently duplicated sesterterpene (C25) gene clusters in *Arabidopsis thaliana* modulate root microbiota. *Science China-Life Sciences* **62**(7): 947-958.

Faldt J, Arimura G, Gershenzon J, Takabayashi J, Bohlmann J. 2003. Functional identification of AtTPS03 as (*E*)-beta-ocimene synthase: a monoterpene synthase catalyzing jasmonate- and wound-induced volatile formation in *Arabidopsis thaliana*. *Planta* **216**(5): 745-751.

Helliwell CA, Sullivan JA, Mould RM, Gray JC, Peacock WJ, Dennis ES. 2001. A plastid envelope location of Arabidopsis *ent*-kaurene oxidase links the plastid and endoplasmic reticulum steps of the gibberellin biosynthesis pathway. *Plant Journal* **28**(2): 201-208.

Herde M, Gartner K, Kollner TG, Fode B, Boland W, Gershenzon J, Gatz C, Tholl D. 2008. Identification and regulation of TPS04/GES, an Arabidopsis geranyllinalool synthase catalyzing the first step in the formation of the insect-induced volatile C(16)-homoterpene TMTT. *Plant Cell* **20**(4): 1152-1168.

Huang AC, Kautsar SA, Hong YJ, Medema MH, Bond AD, Tantillo DJ, Osbourn A. 2017. Unearthing a sesterterpene biosynthetic repertoire in the Brassicaceae through genome mining reveals convergent evolution. *Proceedings of the National Academy of Sciences, USA* **114**(29): E6005-E6014.

Huang MS, Abel C, Sohrabi R, Petri J, Haupt I, Cosimano J, Gershenzon J, Tholl D. 2010. Variation of herbivore-induced volatile terpenes among Arabidopsis ecotypes depends on allelic differences and subcellular targeting of two terpene synthases, TPS02 and TPS03. *Plant Physiology* **153**(3): 1293-1310.

Ro DK, Ehlting J, Keeling CI, Lin R, Mattheus N, Bohlmann J. 2006. Microarray expression profiling and functional characterization of AtTPS genes: Duplicated *Arabidopsis thaliana* sesquiterpene synthase genes At4g13280 and At4g13300 encode root-specific and wound-inducible (*Z*)-gamma-bisabolene synthases. *Archives of Biochemistry and Biophysics* **448**(1-2): 104-116.

Roos J, Bejai S, Mozuraitis R, Dixelius C. 2015. Susceptibility to *Verticillium longisporum* is linked to monoterpene production by TPS23/27 in Arabidopsis. *Plant Journal* **81**(4): 572-585.

Shao J, Chen QW, Lv HJ, He J, Liu ZF, Lu YN, Liu HL, Wang GD, Wang Y. 2017. (+)-Thalianatriene and (-)-retigeranin B catalyzed by sesterterpene synthases from *Arabidopsis thaliana*. *Organic Letters* **19**(7): 1816-1819.

Tholl D, Chen F, Petri J, Gershenzon J, Pichersky E. 2005. Two sesquiterpene synthases are responsible for the complex mixture of sesquiterpenes emitted from Arabidopsis flowers. *Plant Journal* **42**(5): 757-771.

Vaughan MM, Wang Q, Webster FX, Kiemle D, Hong YJ, Tantillo DJ, Coates RM, Wray AT, Askew W, O'Donnell C, *et al*. 2013. Formation of the unusual semivolatile diterpene rhizathalene by the Arabidopsis class I terpene synthase TPS08 in the root stele is involved in defense against belowground herbivory. *Plant Cell* **25**(3): 1108-1125.

Wang Q, Jia MR, Huh JH, Muchlinski A, Peters RJ, Tholl D. 2016. Identification of a dolabellane type diterpene synthase and other root-expressed diterpene synthases in Arabidopsis. *Frontiers in Plant Science* **7**.

Yamaguchi S, Sun TP, Kawaide H, Kamiya Y. 1998. The *GA2* locus of *Arabidopsis thaliana* encodes *ent*-kaurene synthase of gibberellin biosynthesis. *Plant Physiology* **116**(4): 1271-1278.

Methods S1 Details on experimental procedures.

Plant growth conditions

Tomato seeds were germinated on moistened filter paper. After germination, the seedlings were transplanted and grown in soil in a controlled growth room maintained under 16 h of light (300 μE $m^{-2}S^{-1}$; Valoya LED grow lights, Helsinki, Finland) at 28°C and 8 h of dark at 20°C. *Nicotiana benthamiana* and *Arabidopsis thaliana* (Col-0 ecotype) seeds were sown in potting soil, after germination the seedlings were transferred and grown in soil in a growth chamber under controlled conditions of light intensity of 200 μE $m^{-2}S^{-1}$ (mixed cool-white and incandescent bulbs) for 16 h light period, and day/night temperature of 25/20°C.

RNA isolation, cDNA synthesis and quantitative real-time PCR

Total RNA was isolated using the E.Z.N.A Plant RNA Kit (Omega Bio-tek, Norcross, GA, USA) and incubated with DNase I (Promega, Madison, WI, USA) to avoid genomic DNA contamination according to the manufacture's protocol. One microgram of total RNA was used to synthesize cDNA by using the High Capacity cDNA Reverse Transcription Kit (Thermo Fisher, Waltham, MA, USA). For quantitative real-time PCR (RT-qPCR), 1 μL of the 10-fold diluted cDNA and gene-specific primers were applied to 10 μL SYBR Green reactions (PowerUp SYBR Green Master Mix, Thermo Fisher), which were run on a Real-Time PCR System (StepOnePlus, Thermo Fisher) with the following cycles: 95°C for 10 min, followed by 40 cycles of 95°C for 15 s and 60°C for 1 min, and a melting curve analysis. Gene expression values were calculated according to the previously described comparative C_T method (Schmittgen & Livak, 2008). For normalizing gene expressions, *Ubiquitin* (*Solyc11g071260*) was used as a control. Three biological replicates were used for each point with three or more technical replicates. Primers used are listed in Table S1.

Protein expression in *Escherichia coli* and purification

For enzymatic assays, TPS genes without their fragments corresponding to putative transit peptides were cloned upstream and in-frame of the $(His)_6$-tag sequences in pET32b to express TPS-His recombinant proteins. Restriction sites and primers used are detailed in Table S1. The resulting recombinant plasmids were sequence verified and transformed into *E. coli* Rosetta2(DE3)pLysS cells (Novagen, Burlington, MA, USA). Single positive colonies were used to inoculate 4 mL LB medium containing 34 ng μL^{-1} chloramphenicol and 100 ng μL^{-1} ampicillin. One mL of the overnight culture was transferred to 100 mL of the same medium and continued to grow at 37°C to reach an OD_{600} of approximately 0.3. Protein expression was induced by adding isopropyl β-thiogalactopyranoside (IPTG) to a final concentration of 1 mM. After 16 h incubation at 16°C, cells were harvested by centrifugation and stored at -80°C until use. The recombinant His-tagged proteins were affinity purified using nickel-nitrilotriacetic acid (Ni-NTA) agarose (Qiagen, Hilden, Germany) according to the manufacturer's manuals. Protein concentration was determined using the Braford method (Braford, 1976) with BSA as a standard.

GC-MS analysis of terpenes

The analysis of terpene products (C_{10}-C_{25}) was performed as described previously (Falara *et al*., 2011). One microliter of sample was auto-injected onto a Shimadzu (Kyoto, Japan) QP-2010 SE GC-MS system equipped with the Rxi-5Sil column (30 m length, 0.25 mm i.d., and 0.25 µm film thickness; Restek, Bellefonte, PA, USA). The injection port was set to 240°C in splitless mode, and the flow rate of the He carrier gas was set at 1.4 mL min^{-1}. The GC oven temperature was programmed as follows: 45°C for 2 min, 10°C min^{-1} up to 250°C and hold for 3 min, 20°C min^{-1} up to 320°C and hold for 5 min. The

analysis of prenyltransferase assay products was performed using an EC-WAX column (30 m length, 0.32 mm i.d., and 0.25 μm film thickness; Grace, Columbia, MD, USA). The injection port was set to 220°C in splitless mode, and the flow rate of the He carrier gas was set at 1.5 mL min^{-1}. The GC oven temperature was programmed as follows: 45°C for 2 min, 10°C min^{-1} up to 260°C and hold for 5 min. Interface temperature was 280°C and ion source temperature was set to 260°C. The detector was activated after a 4.5-min solvent delay. Mass spectra were obtained using the scan mode (total ion count, 45-450 mz^{-1}). The Kováts retention indices (Babushok *et al.*, 2011) of each terpene compounds were determined based on a comparison with (C_7-C_{40}) n-alkanes. Products were identified using the mass spectra database NIST11 and their mass spectra and retention indices were compared with authentic compounds or the literature (Table S2).

Analysis of isoprene was performed by directly injecting the SPME fiber into the Shimadzu QP-2010 SE GC-MS system equipped with the Rxi-5Sil column (30 m length, 0.25 mm i.d., and 0.25 μm film thickness; Restek). The injection port was set to 220°C in splitless mode, and the flow rate of the He carrier gas was set at 1.0 mL min^{-1}. The GC oven temperature was programmed as follows: 40°C for 3 min, 10°C min^{-1} up to 100°C and hold for 3 min, 20°C min^{-1} up to 260°C and hold for 3 min. Isoprene was identified by comparison with the authentic standard.

Correlation analysis of tomato terpene synthase genes and terpene volatiles

The Spearman correlation coefficients were calculated by GraphPad Prism 6 using gene expression data and terpene profiling data as indicated in Fig. 9 and Fig. 10, respectively. Based on the Spearman correlation coefficient, the hierarchical clustering was performed using average linkage method by Cluster 3.0 and a heatmap was built using TreeView 3.0.

Accession numbers

The GenBank accession numbers of the proteins used in the phylogenetic analysis (Fig. S9) are as follows: AgGPPS1, AF513111; AgGPPS2, AF513112; AgGPPS3, AF513113; PaGPPS, EU432047; PaGPPS/GGPPS, GQ369788; PaGPPS3, EU432048; MiGPPS1, JN035297; MiGPPS2, JN035298; MiFPPS, JN035296; AmGPPS.LSU, AAS82860; AmGPPS.SSU, AAS82859; CbGPPS.SSU, AY534745; CrGGPPS, JX417183; CrGPPS.SSU, JX417184; HlGPPS.LSU, FJ455407; HlGPPS.SSU, ACQ90681; MpGPPS.LSU, AF182828; MpGPPS.SSU, AF182827; VvGPPS, AAR08151; LaFPPS, U15777; AaFPPS, U36376; ZmFPPS, L39789; NtGGPPS1, GQ911583; NtGGPPS2, GQ911584; SaGGPPS, CAA67330; HbSPPS, DQ437520; OsGGPPS1, Os07g39270; OsGPPS, Os01g14630; OsGRP; Os02g44780. AGI numbers for *A. thaliana* TPTs are as follows: AtPPPS, At2g434630; AtFPPS1, At5g47770; AtFPPS2, At4g17190; AtSPPS1, At1g78510; AtSPPS2, At1g17050; AtGGPPS11, At4g36810; AtGGPPS2, At2g18620; AtSSU, At4g38460. Tomato TPT homologs are listed in Table S5.

References

Babushok VI, Linstrom PJ, Zenkevich IG. 2011. Retention indices for frequently reported compounds of plant essential oils. *Journal of Physical and Chemical Reference Data* **40**(4).

Bradford MM. 1976. A rapid and sensitive method for the quantitation of microgram quantities of protein utilizing the principle of protein-dye binding. *Analytical Biochemistry* **72**: 248-254.

Falara V, Akhtar TA, Nguyen TT, Spyropoulou EA, Bleeker PM, Schauvinhold I, Matsuba Y, Bonini ME, Schilmiller AL, Last RL, et al. 2011. The tomato terpene synthase gene family. *Plant Physiology* **157**(2): 770-789.

Schmittgen TD, Livak KJ. 2008. Analyzing real-time PCR data by the comparative C($_T$) method. *Nature Protocol* **3**(6): 1101-1108.
